# Supplementary material for: K2CO3-Promoted Formal [3+3]-Cycloaddition of N-Unsubstituted Isatin N,N′-Cyclic Azomethine Imine 1,3-Dipoles with Knoevenagel Adducts
Source: Molecules. 2023 Jan 19;28(3):1034. doi: 10.3390/molecules28031034 (PMC9921867; doi:10.3390/molecules28031034)
Supplement: Supplementary file 1 [file molecules-28-01034-s001.zip › molecules-2155705-supplementary.pdf]

Molecules

Supporting Information

K_2CO_3-promoted formal [3+3]-cycloaddition of N-unsubstituted isatin N,N′-cyclic azomethine imine 1,3-dipoles with Knoevenagel adducts

Guosheng Yang [1,‡], Sicheng Li [2,‡], Qiumi Wang [1,‡], Huabao Chen [3], Chunping Yang [3], Zhongqiong Yin [4], Xu Song [4], Li Zhang [1], Cuifen Lu [5] and Guizhou Yue [1,*]

[1]*College of Science, Sichuan Agricultural University, Ya'an, Sichuan, 625014, China.*
[2]*The Yingjing County Emergency Management Agency, Ya'an, Sichuan, 625200, China*
[3]*College of Agronomy, Sichuan Agricultural University, Chengdu, Sichuan, 611130, China.*
[4]*College of Veterinary Medicine, Sichuan Agricultural University, Chengdu, Sichuan, 611130, China.*
[5]*Hubei Collaborative Innovation Center for Advanced Organochemical Materials & Ministry-of-Education Key Laboratory for the Synthesis and Application of Organic Functional Molecules, Hubei University, Wuhan 430062, China*

^{1}H and ^{13}C NMR Spectra for Compound **3a**

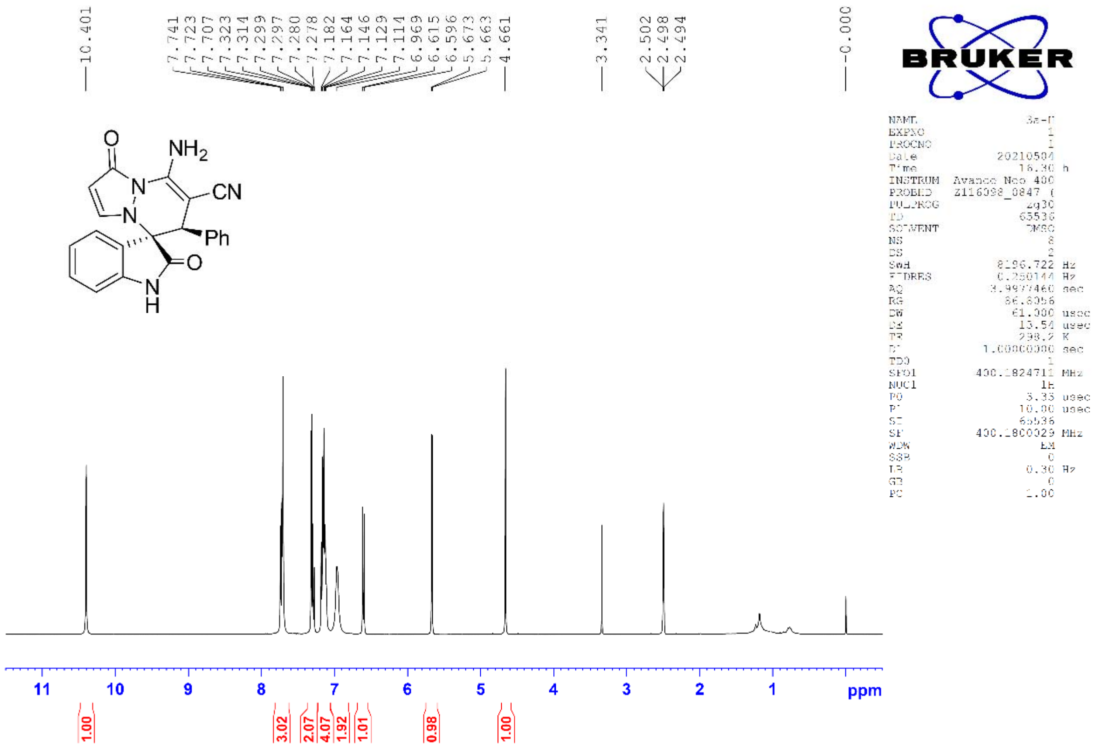

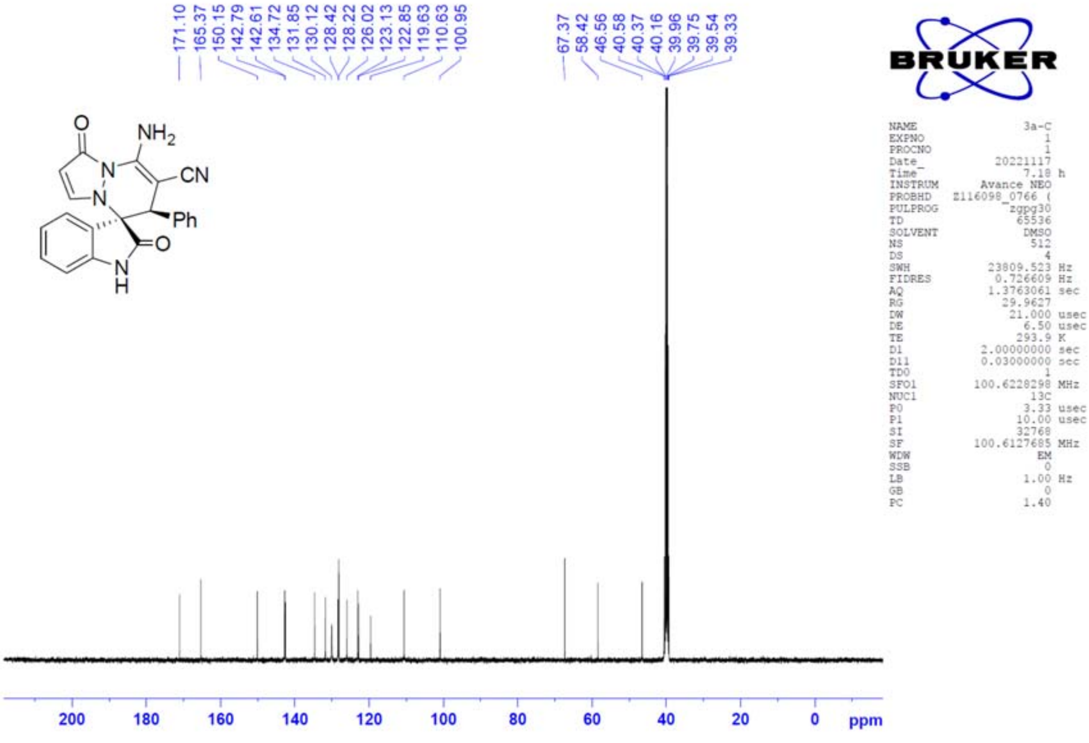

^{1}H and ^{13}C NMR Spectra for Compound **3’b** (containing a few of **3b**)

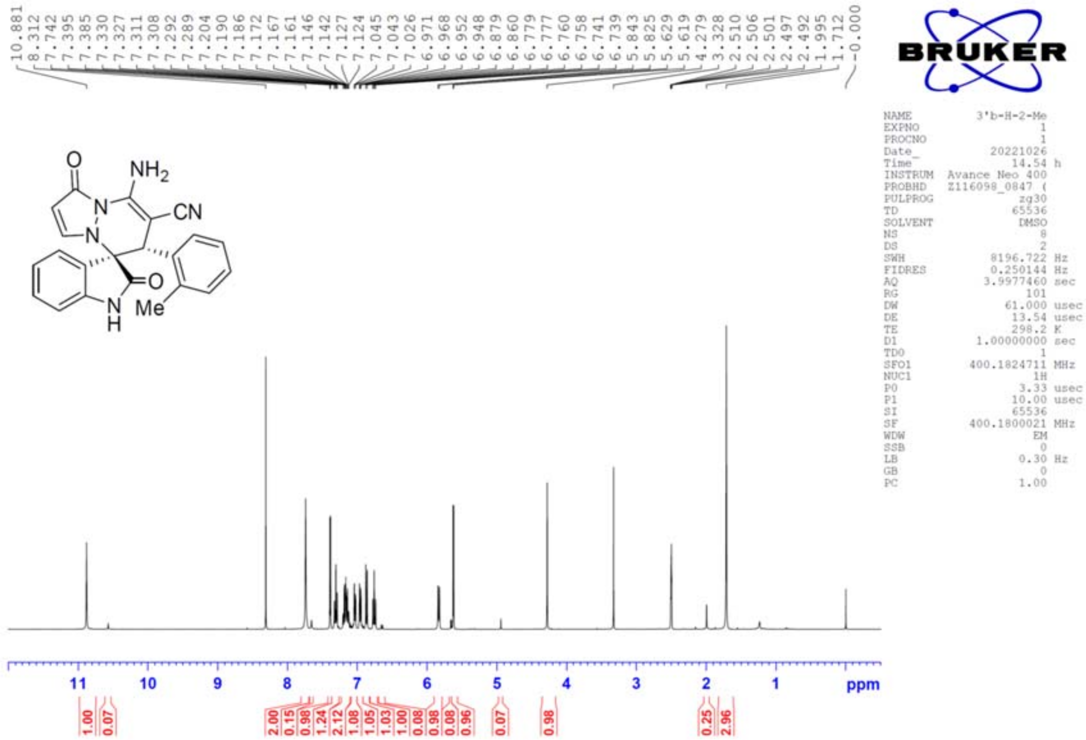

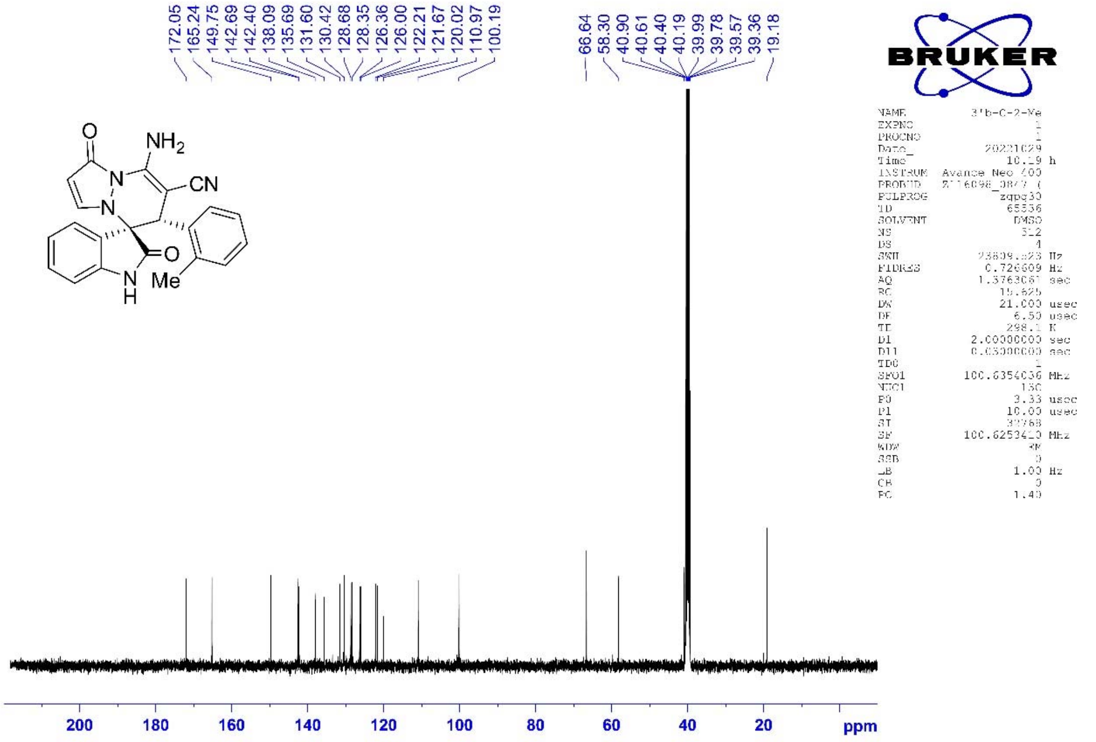

^{1}H and ^{13}C NMR Spectra for Compound **3’c**

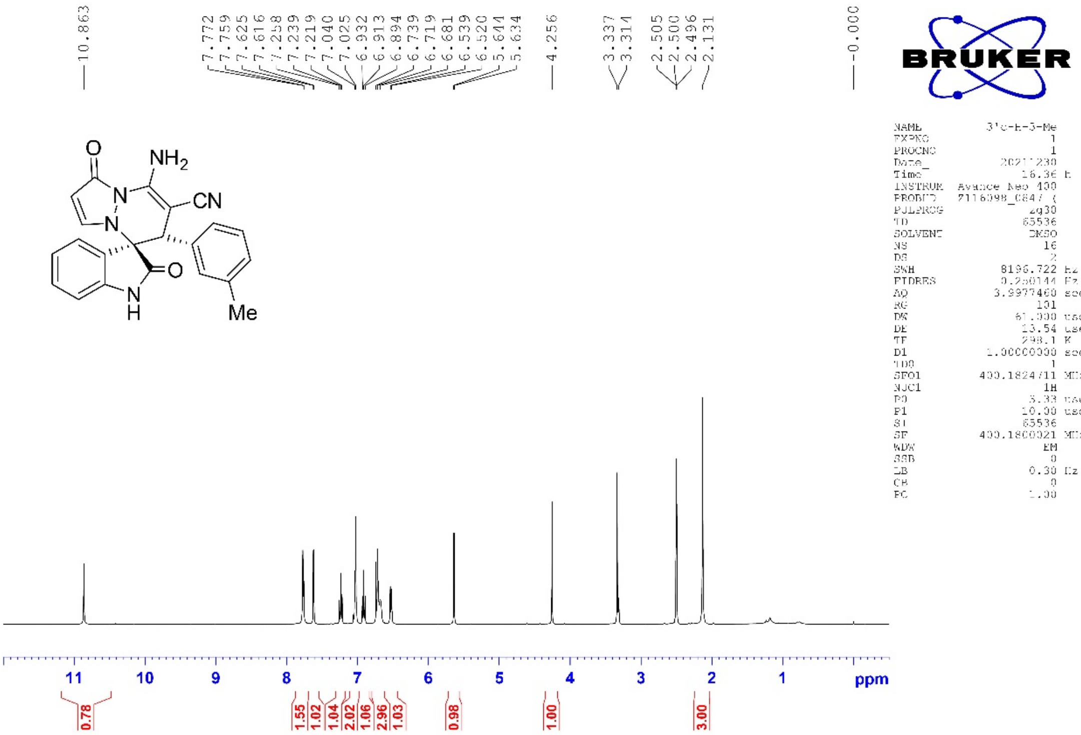

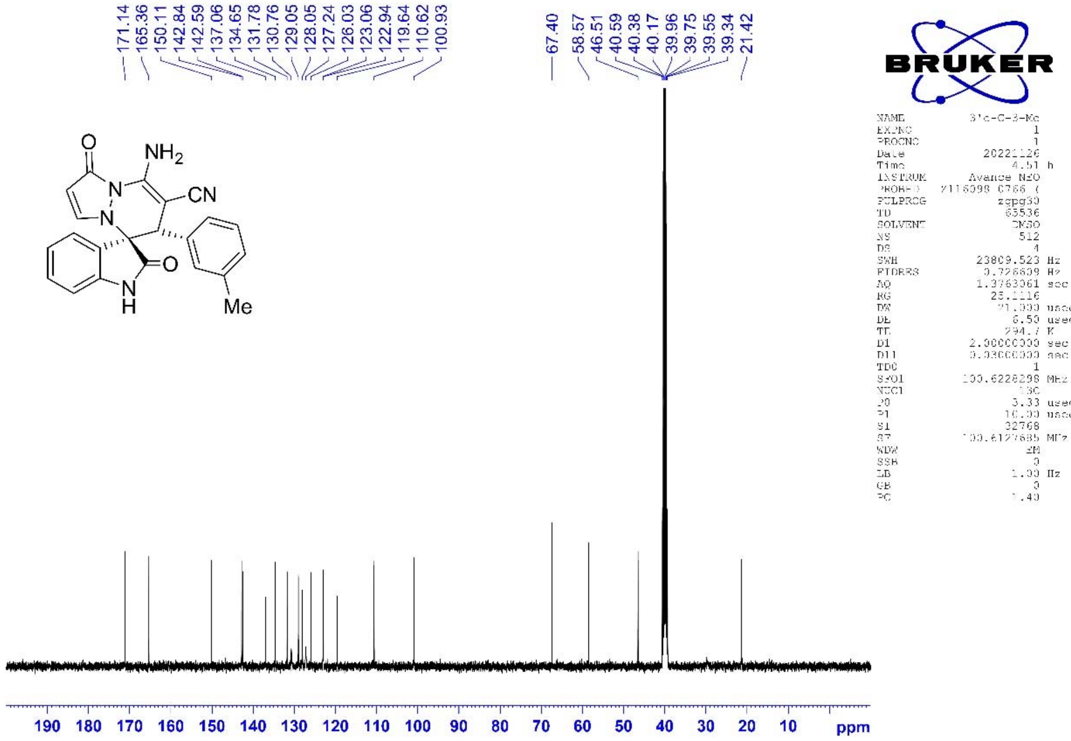

^{1}H and ^{13}C NMR Spectra for Compound **3d**

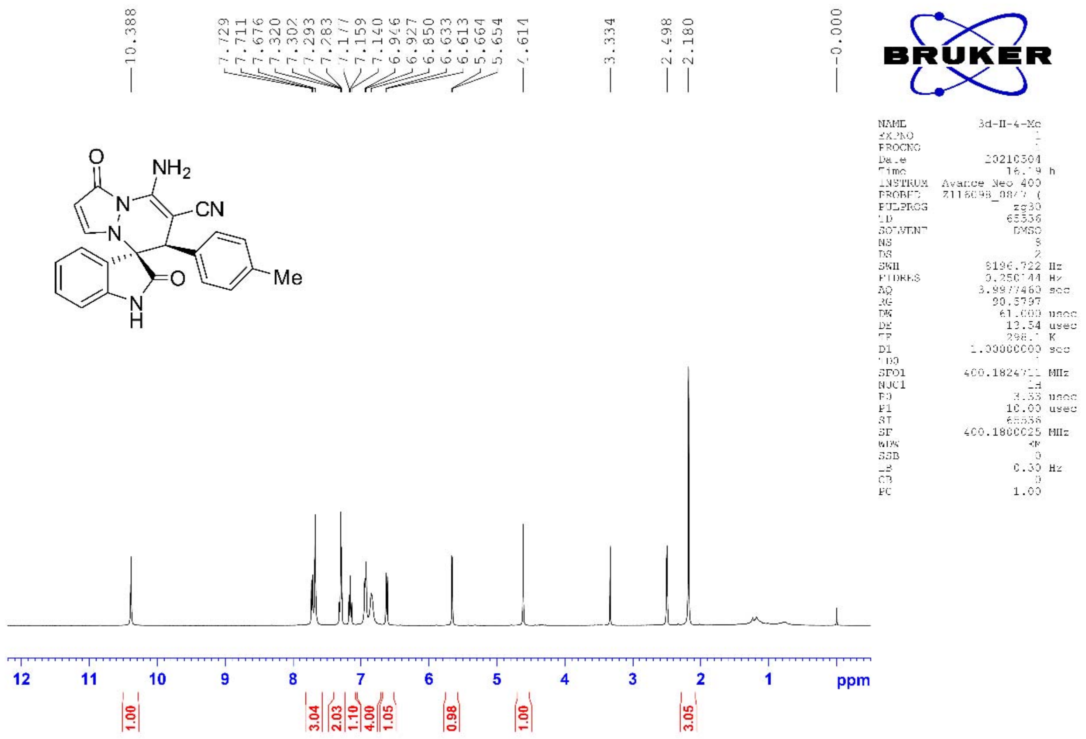

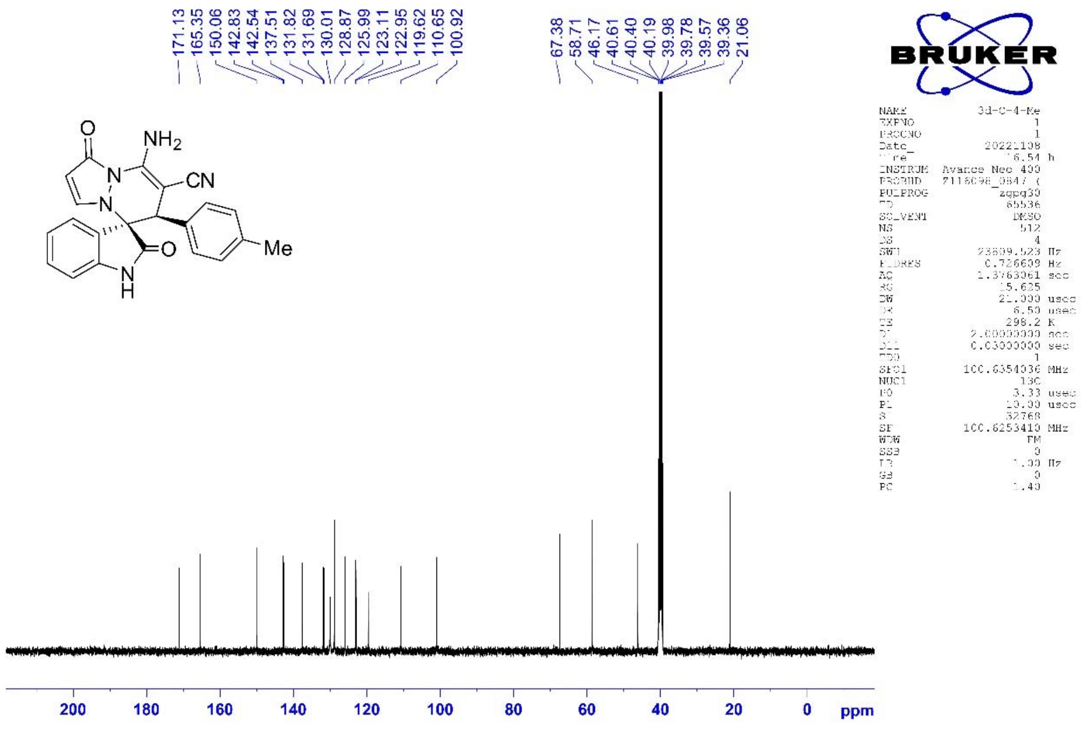

^{1}H and ^{13}C NMR Spectra for Compound **3e**

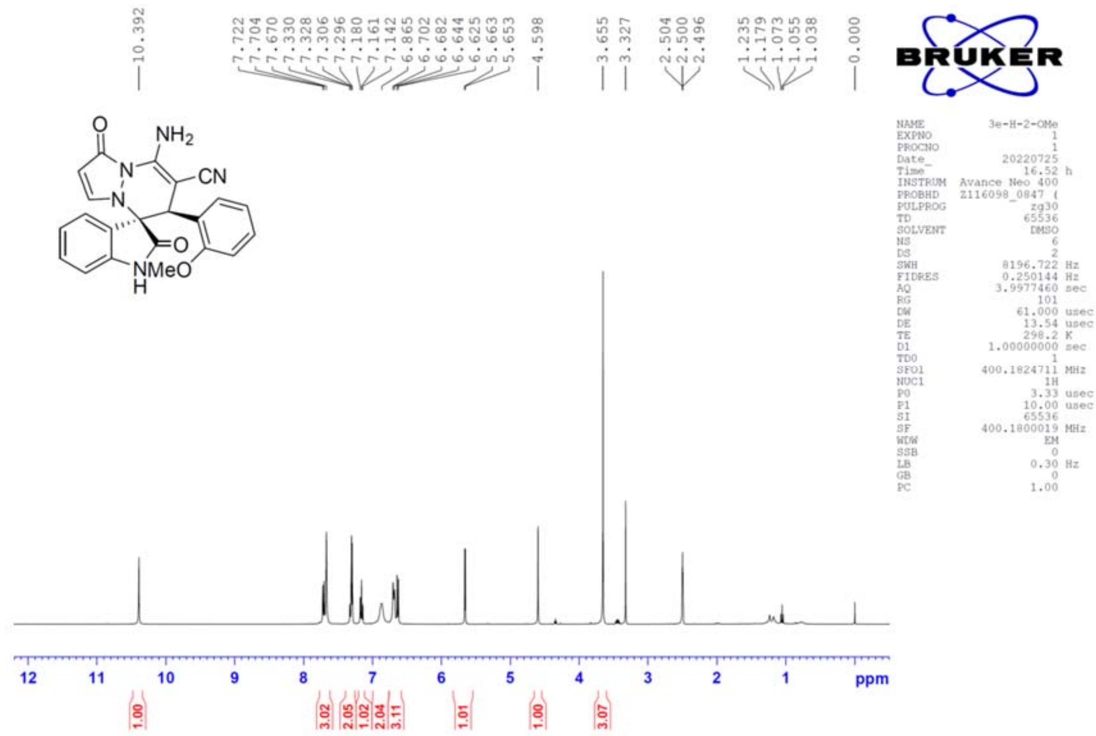

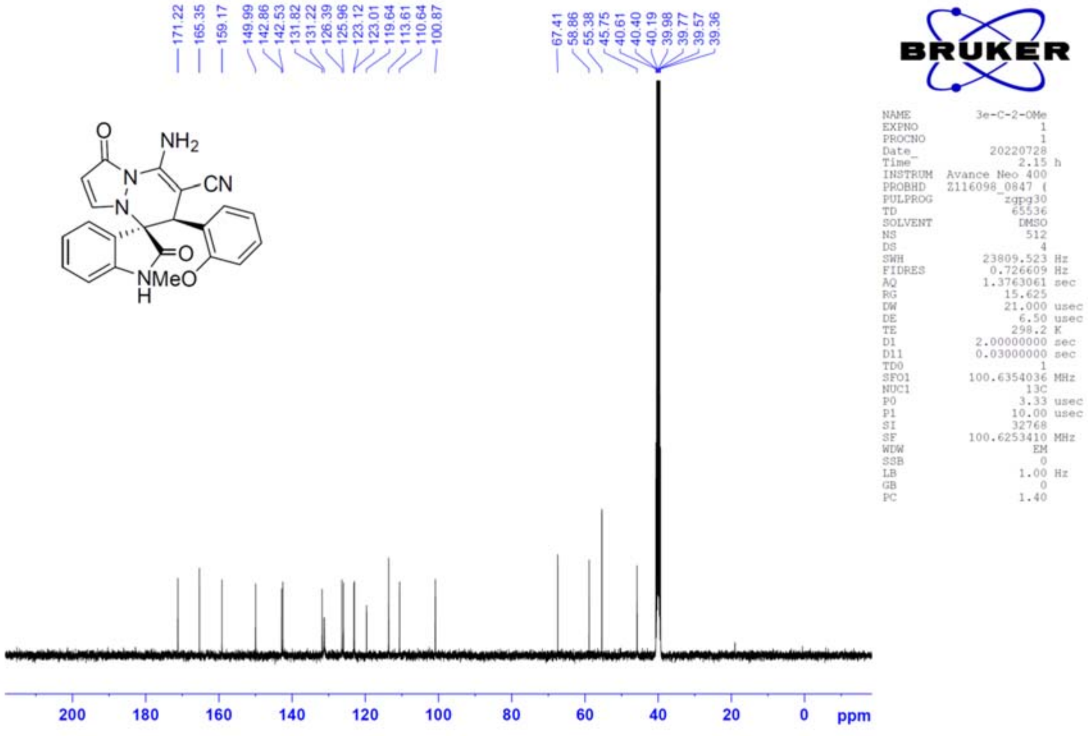

^{1}H and ^{13}C NMR Spectra for Compound **3f**

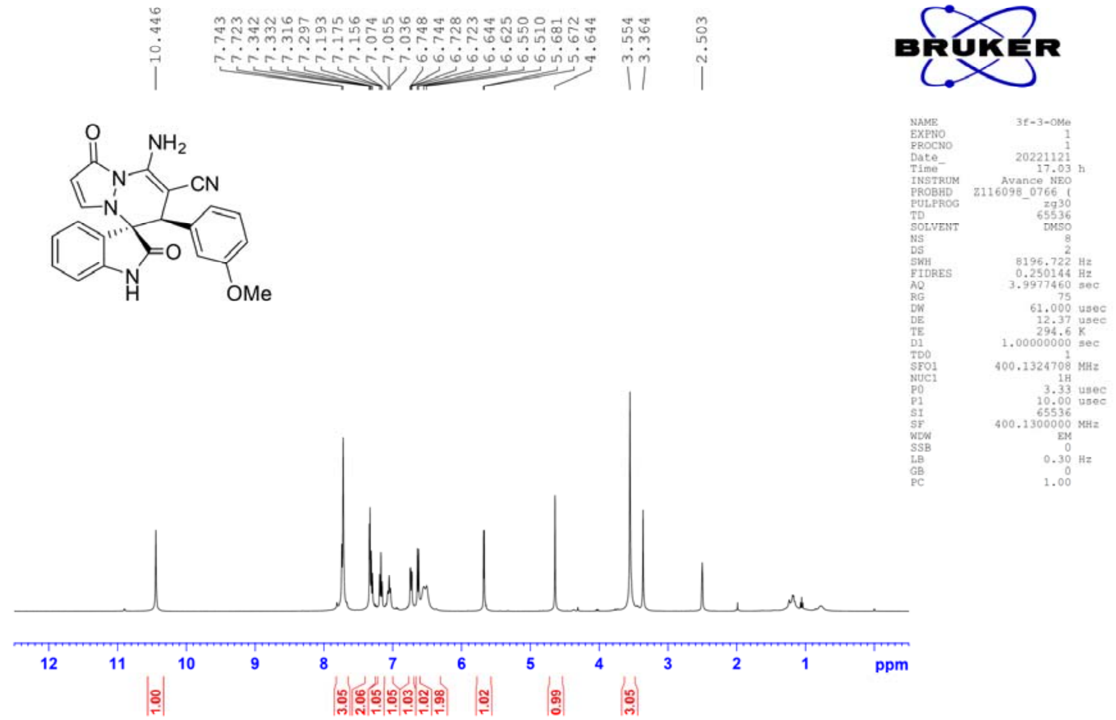

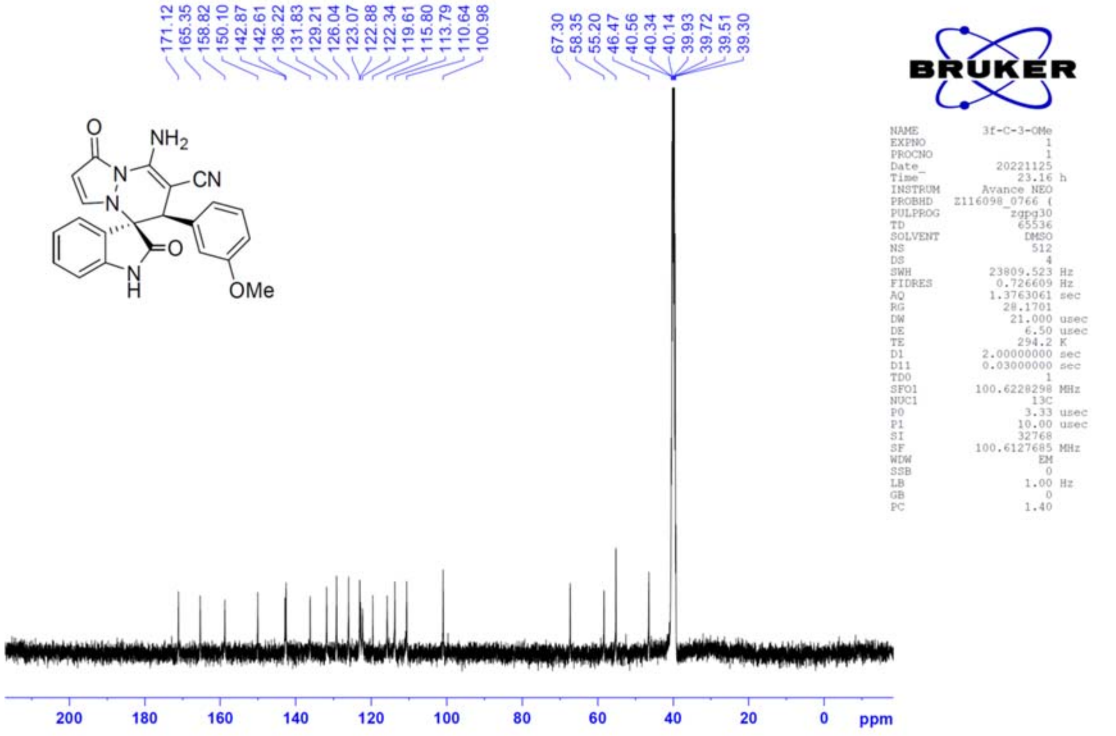

^{1}H and ^{13}C NMR Spectra for Compound **3g**

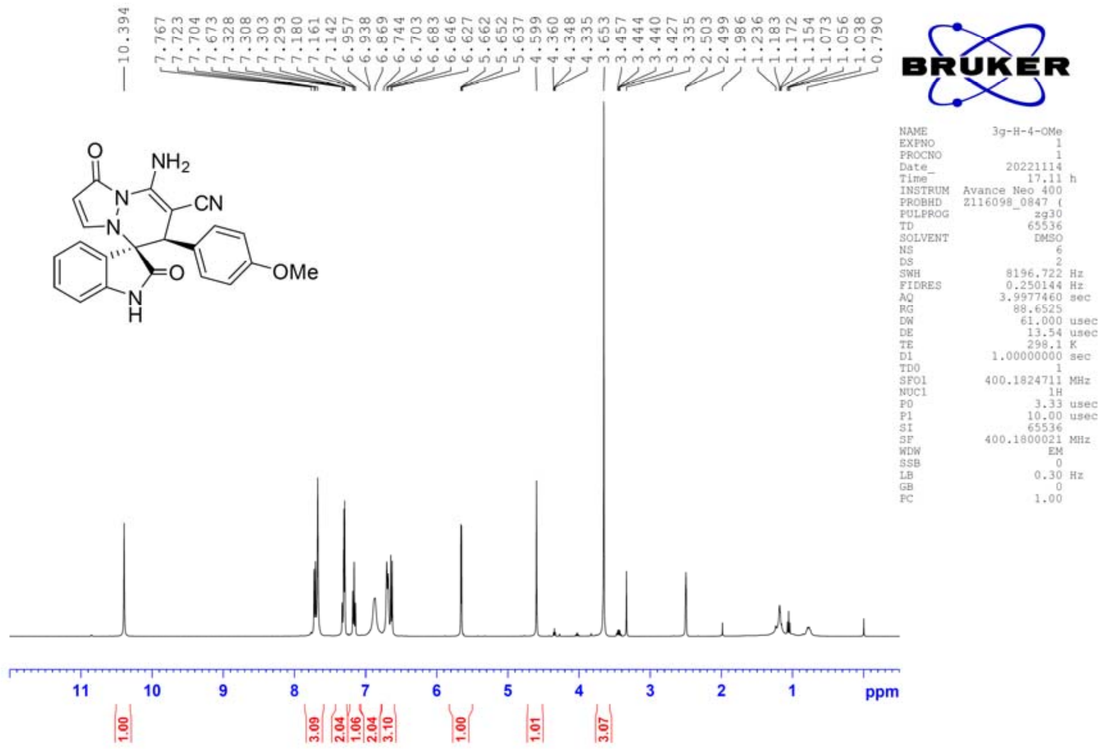

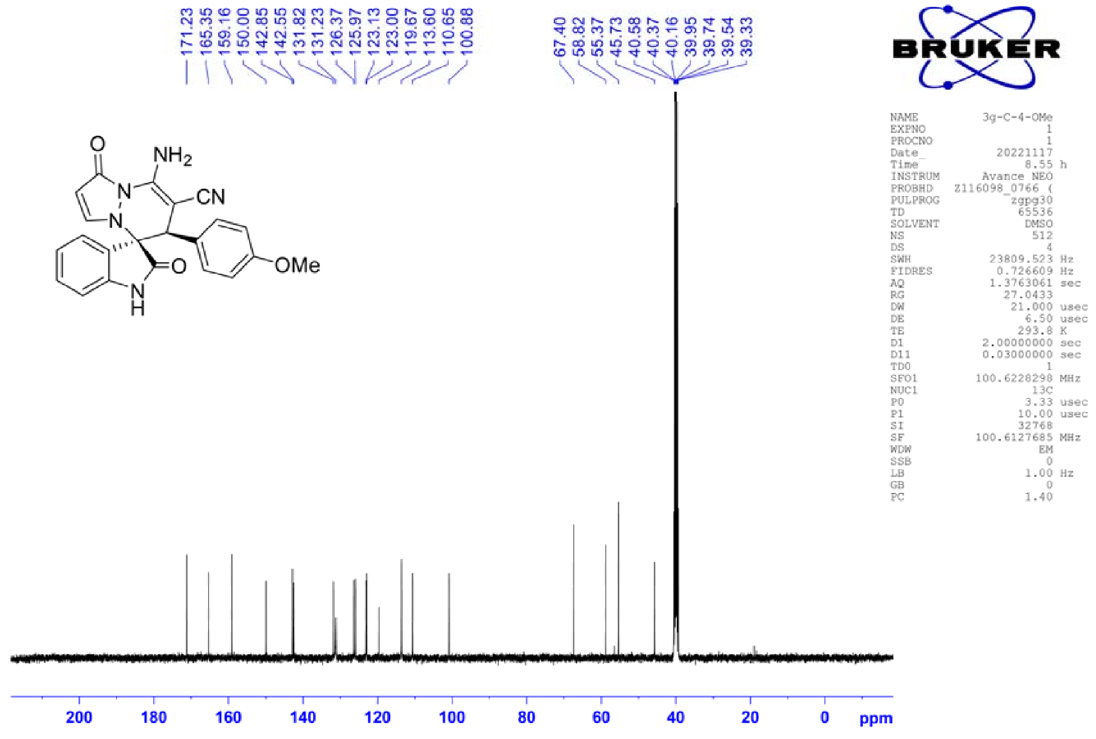

^{1}H, ^{13}C and ^{19}F NMR Spectra for Compound **3h**

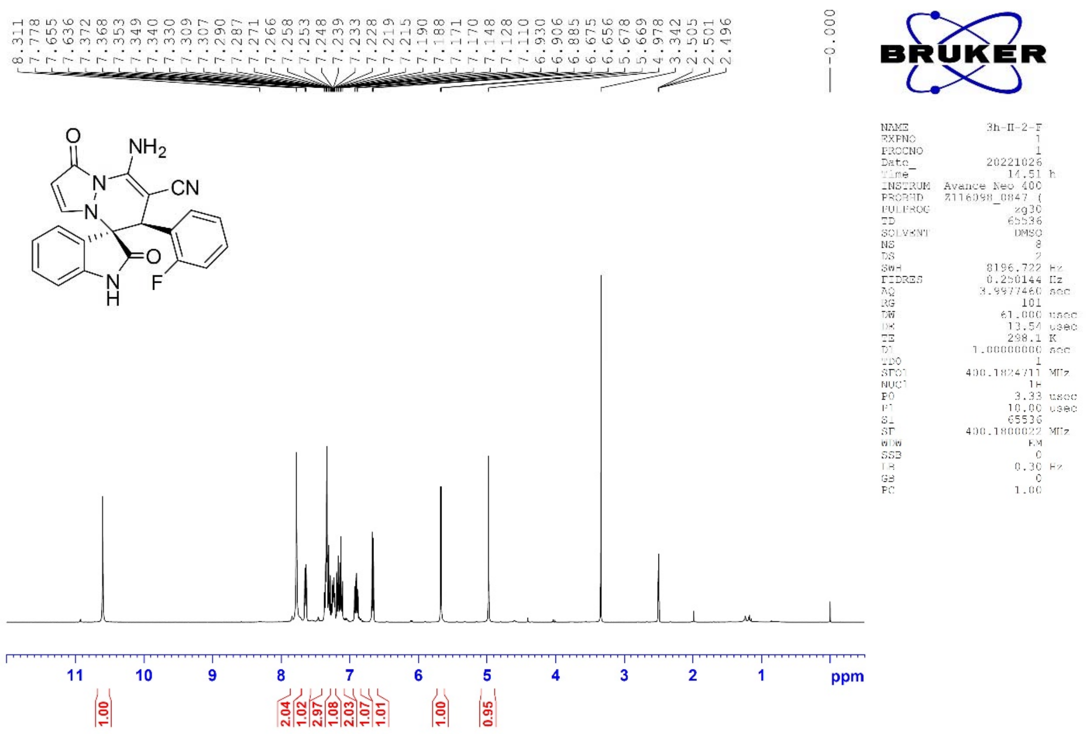

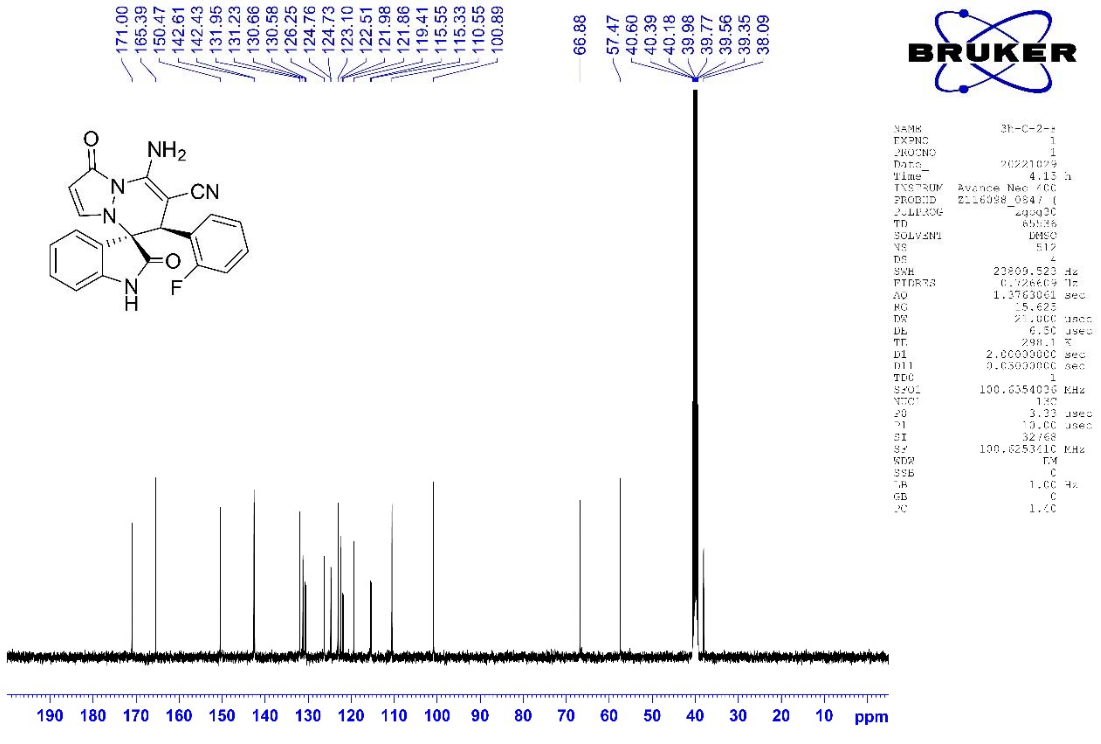

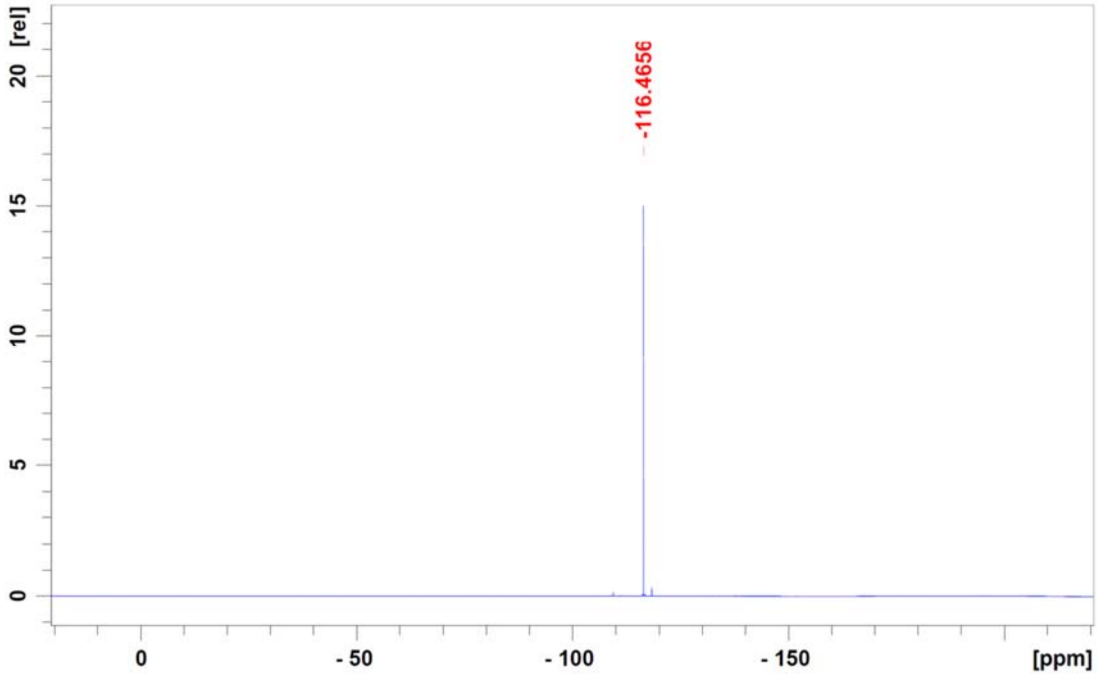

^{1}H, ^{13}C and ^{19}F NMR Spectra for Compound **3i**

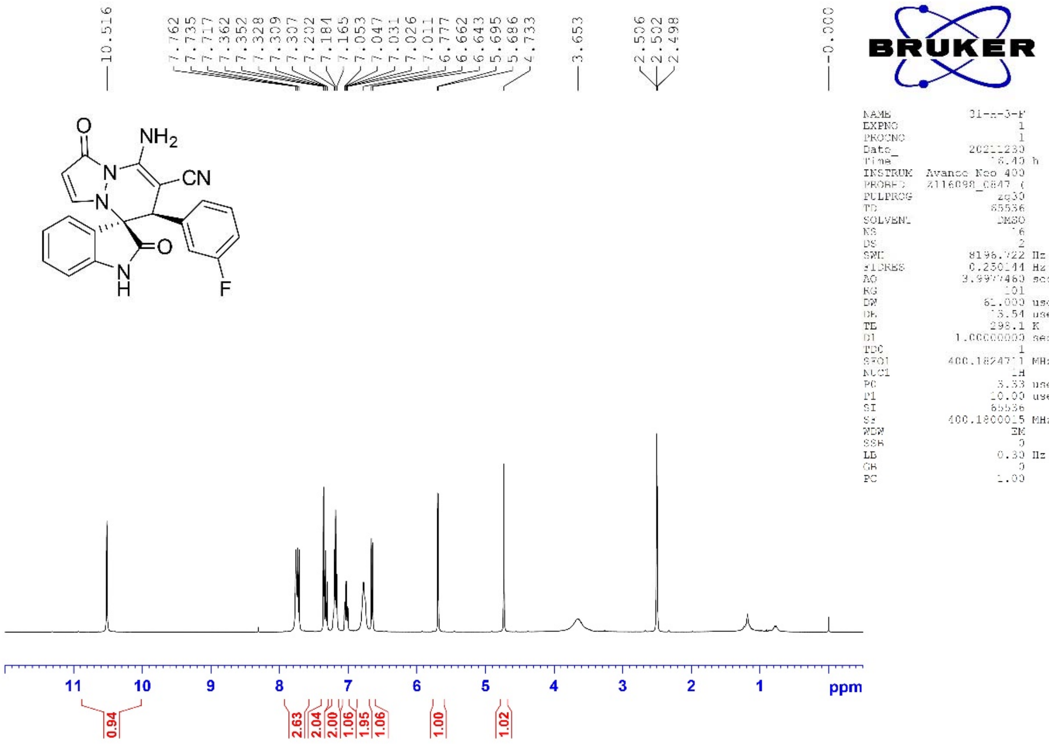

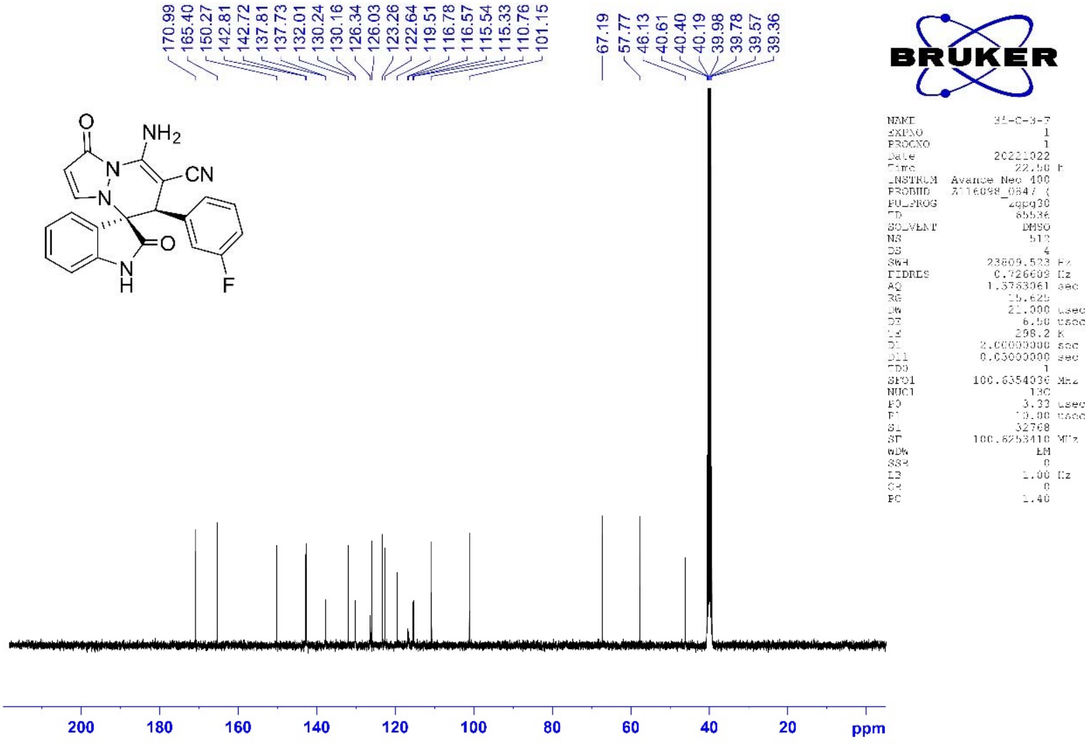
170.99
165.40
150.27
142.81
142.72
137.81
137.73
132.01
130.24
130.16
126.34
126.03
123.26
122.64
119.51
116.78
116.57
115.54
115.33
110.76
101.15
67.19
57.77
46.13
40.61
40.40
40.19
39.98
39.78
39.57
39.36
BRUKER
O
NH2
N
CN
N
O
N
H
F
200
180
160
140
120
100
80
60
40
20
ppm

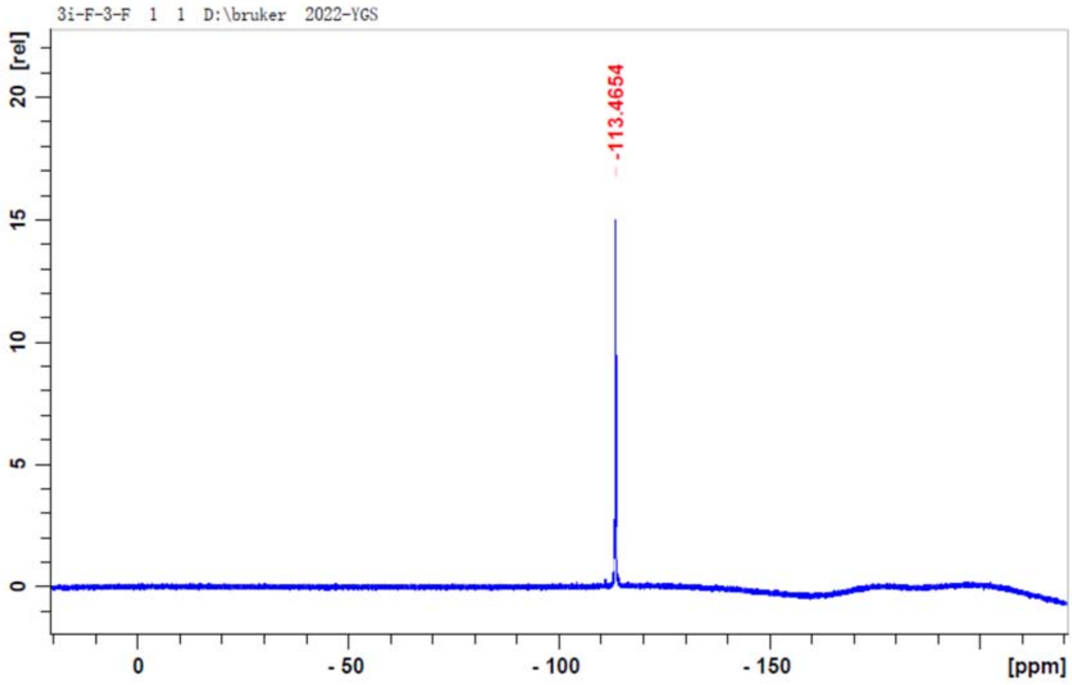
3i-F-3-F 1 1 D:\bruker 2022-YGS
-113.4654
[rel]
20
15
10
5
0
0
- 50
- 100
- 150
[ppm]

^{1}H, ^{13}C and ^{19}F NMR Spectra for Compound **3j**

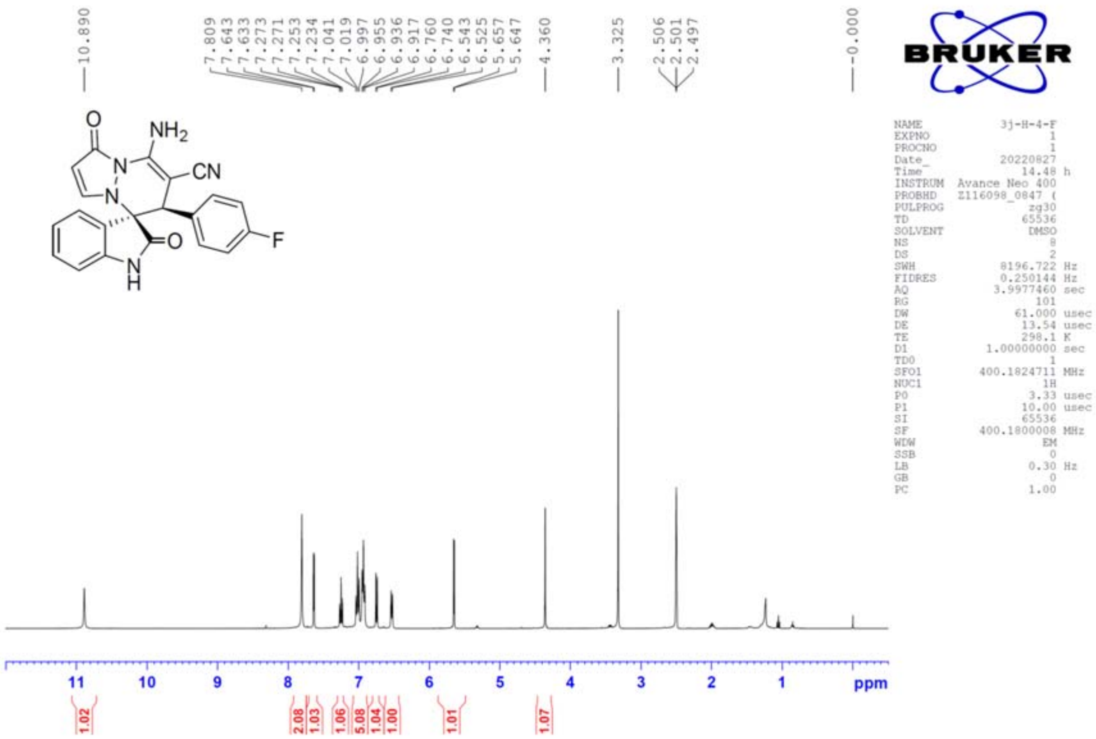

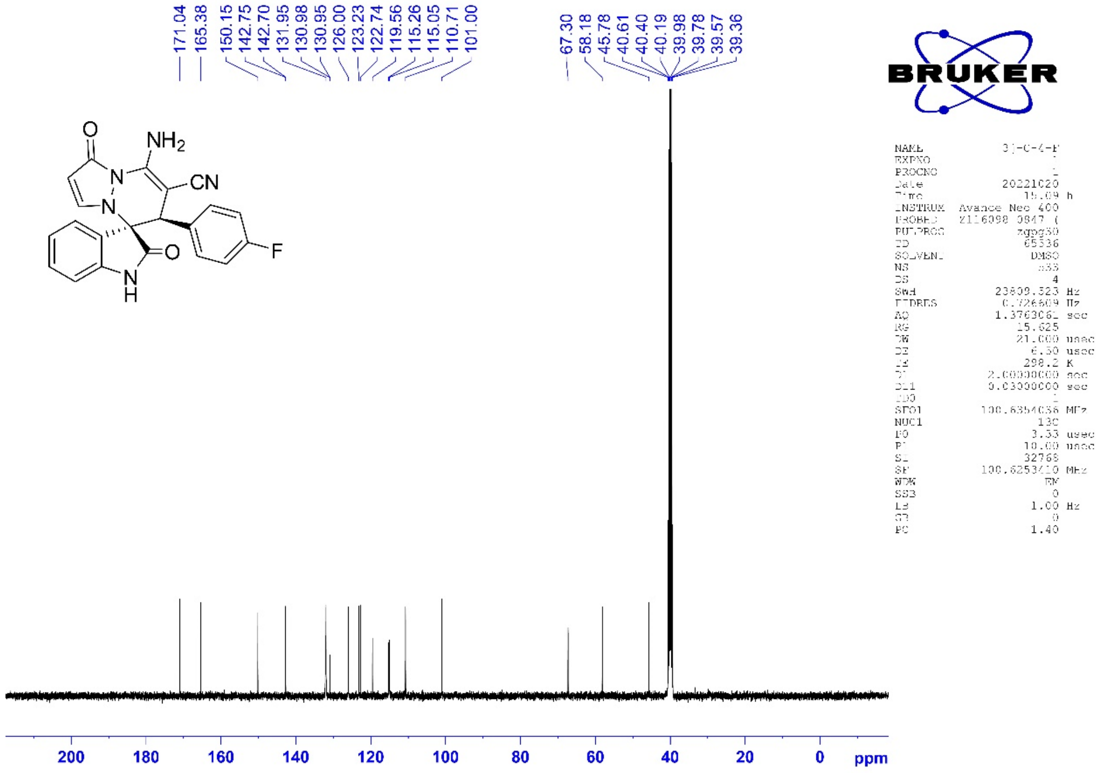

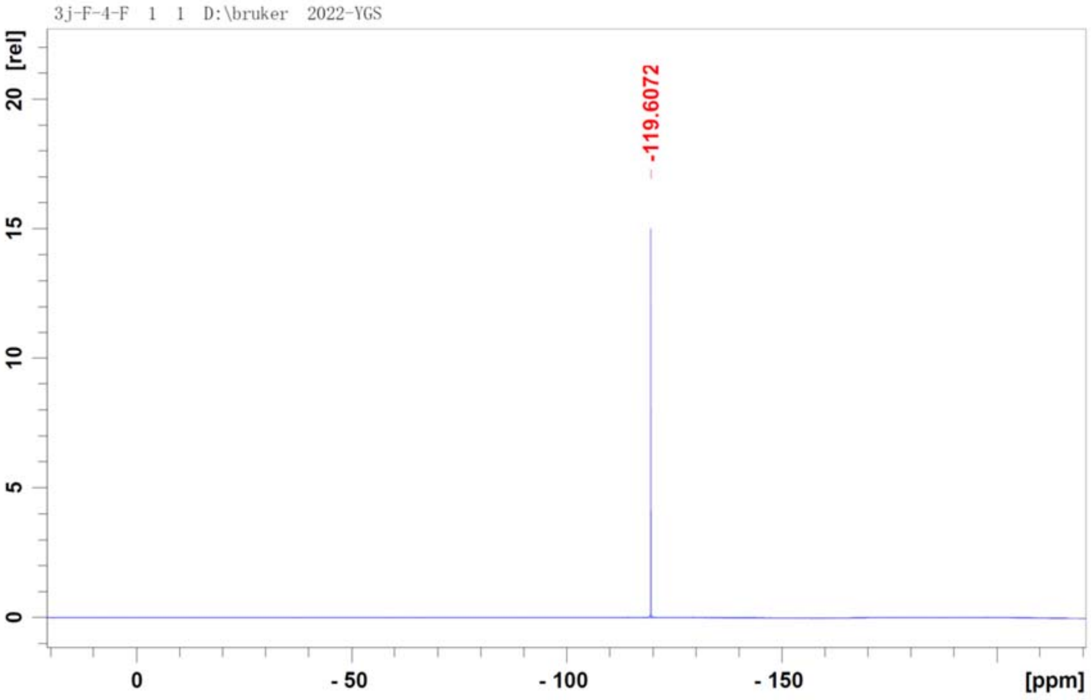

^{1}H and ^{13}C NMR Spectra for Compound **3'k**

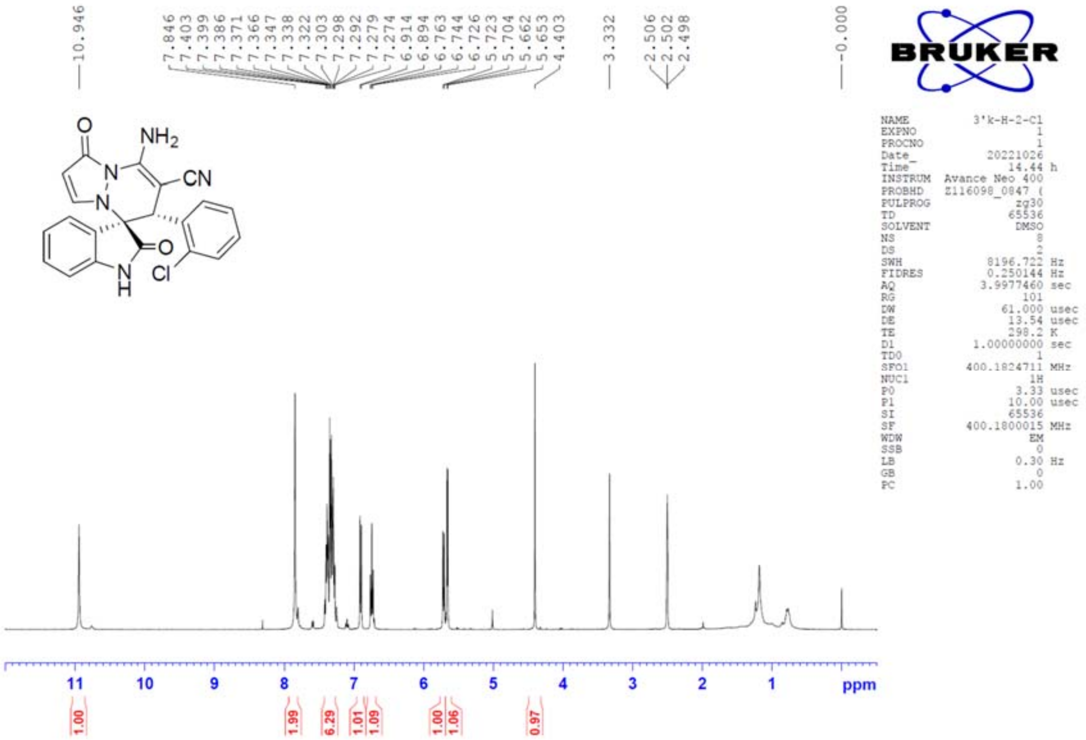

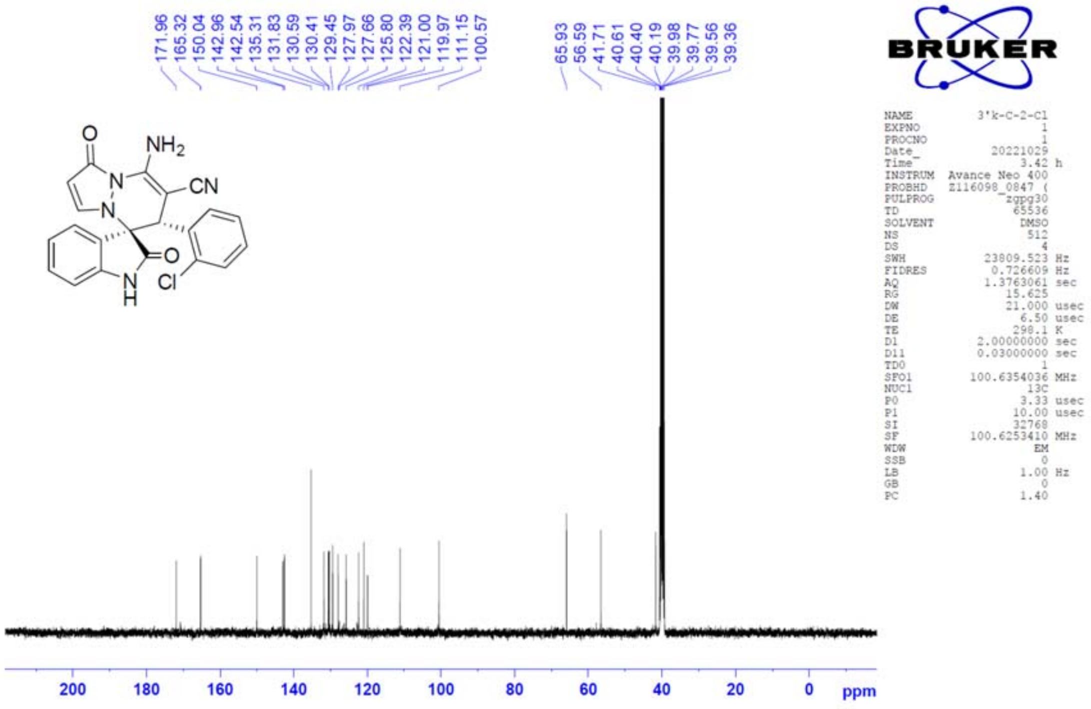

^{1}H and ^{13}C NMR Spectra for Compound **3l**

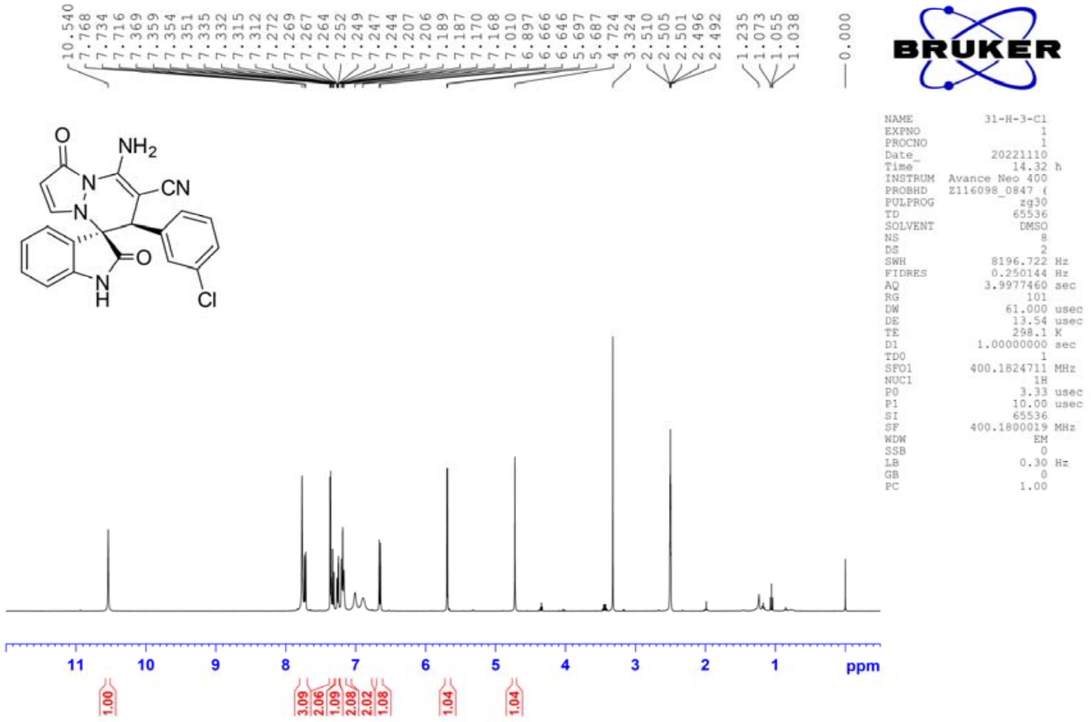

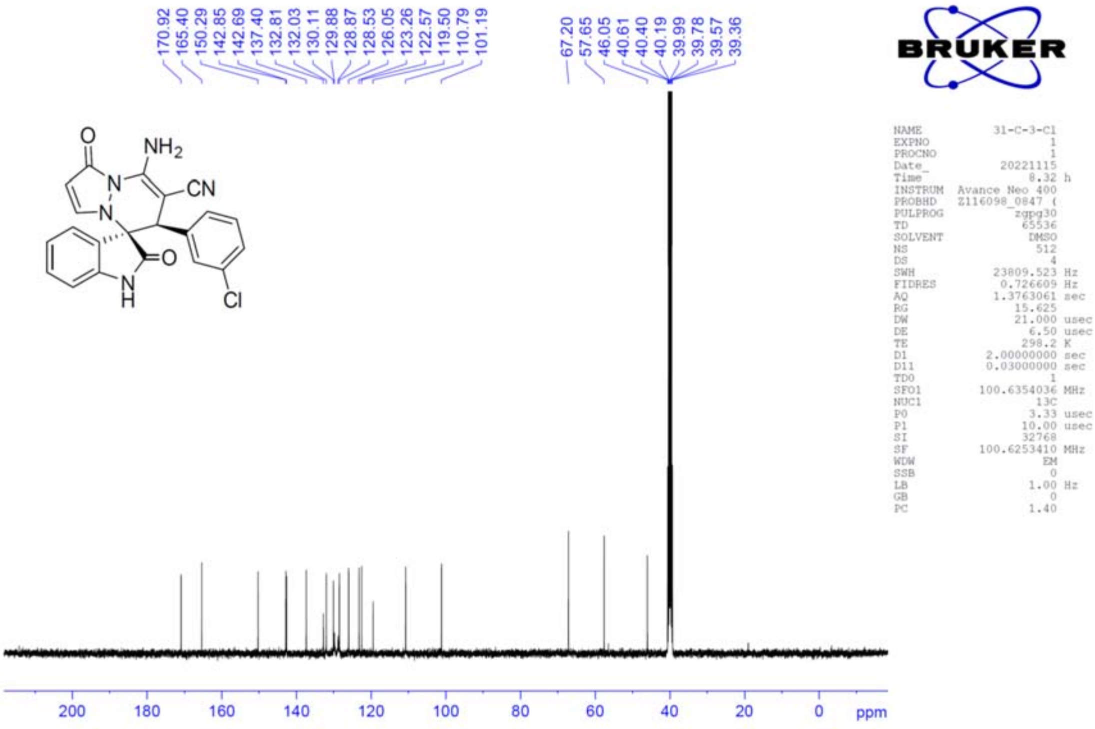

^{1}H and ^{13}C NMR Spectra for Compound **3m (3′m 是主产物)**

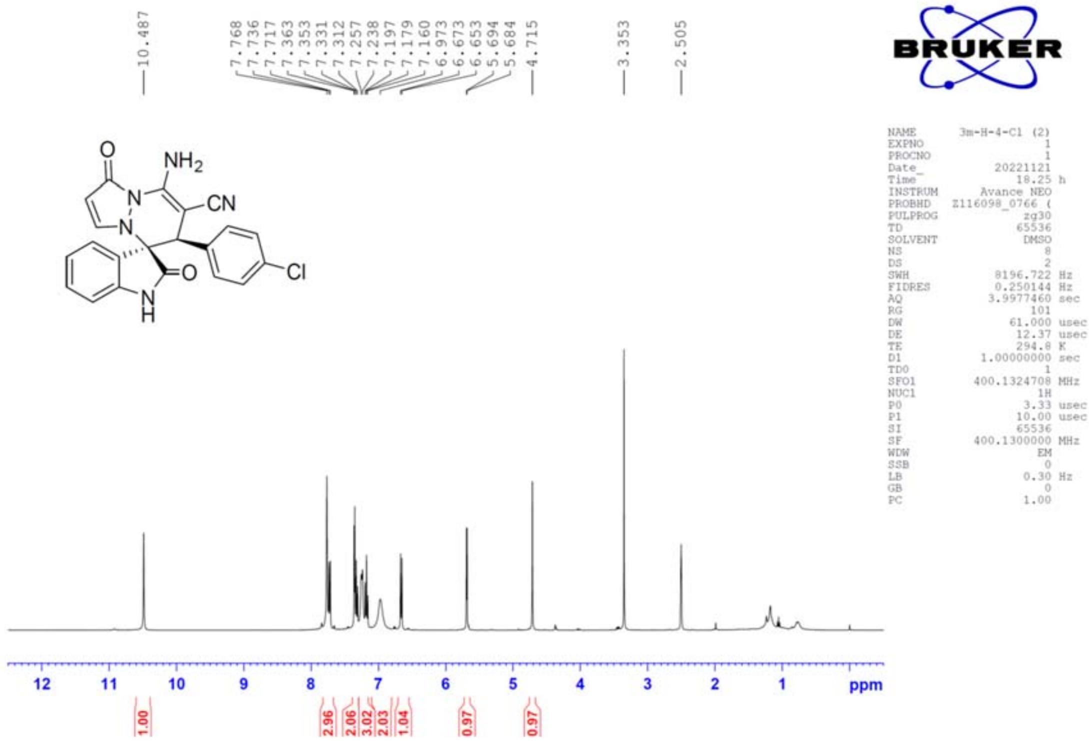

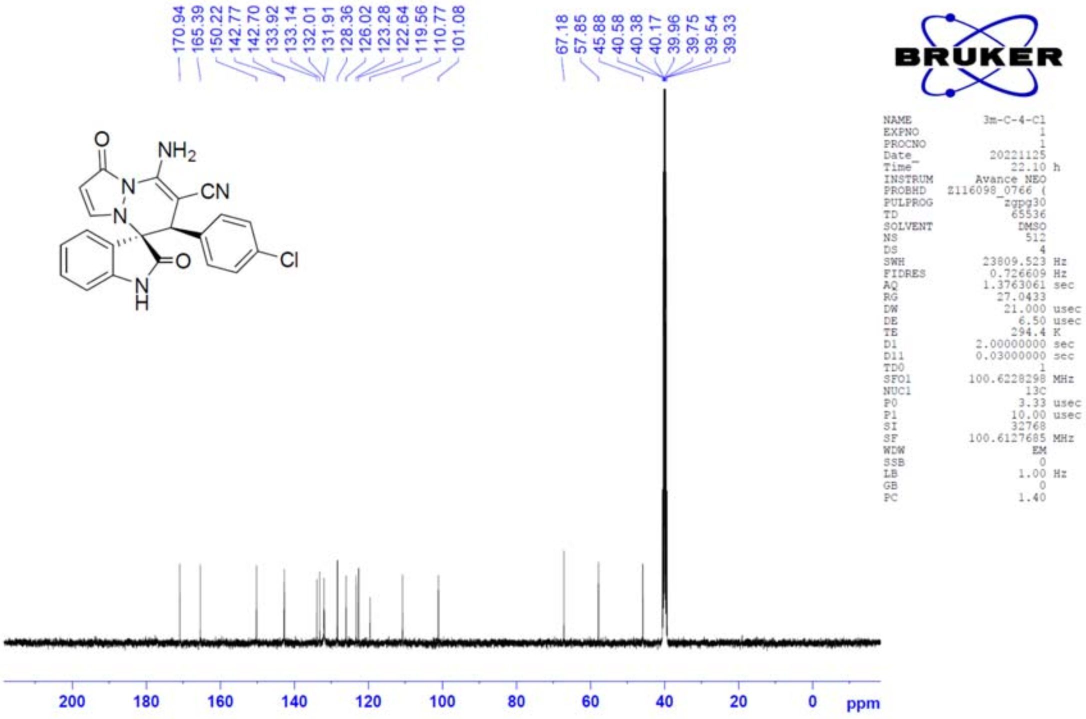

^{1}H and ^{13}C NMR Spectra for Compound **3’n**

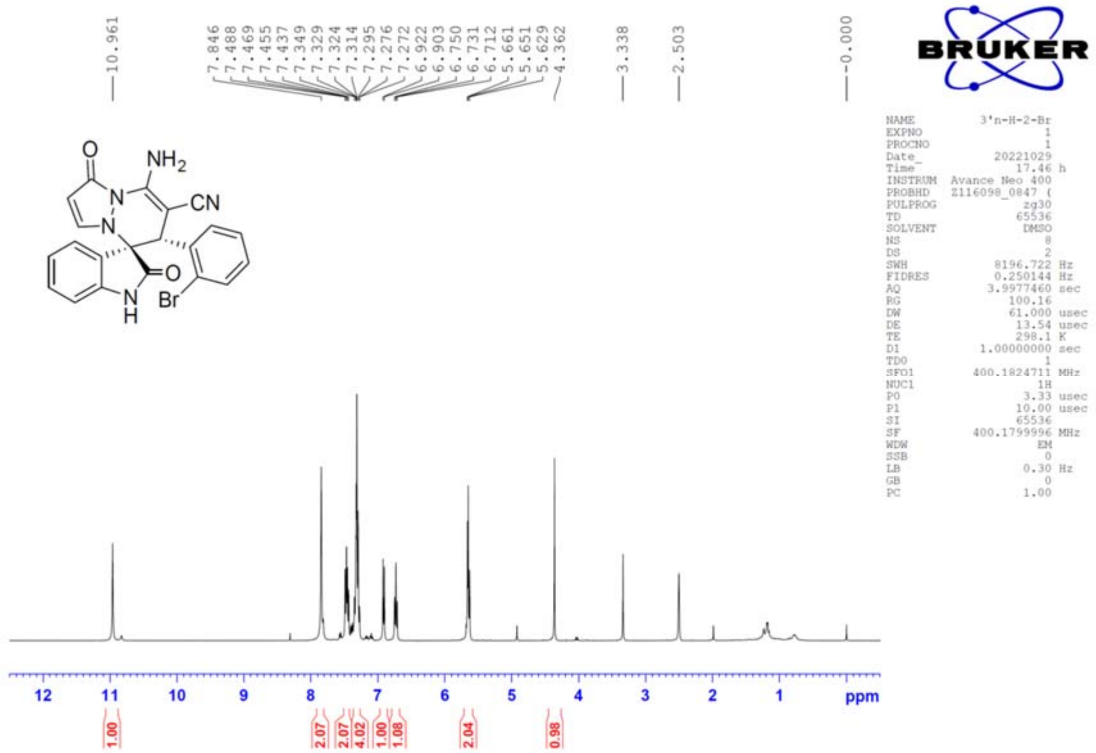

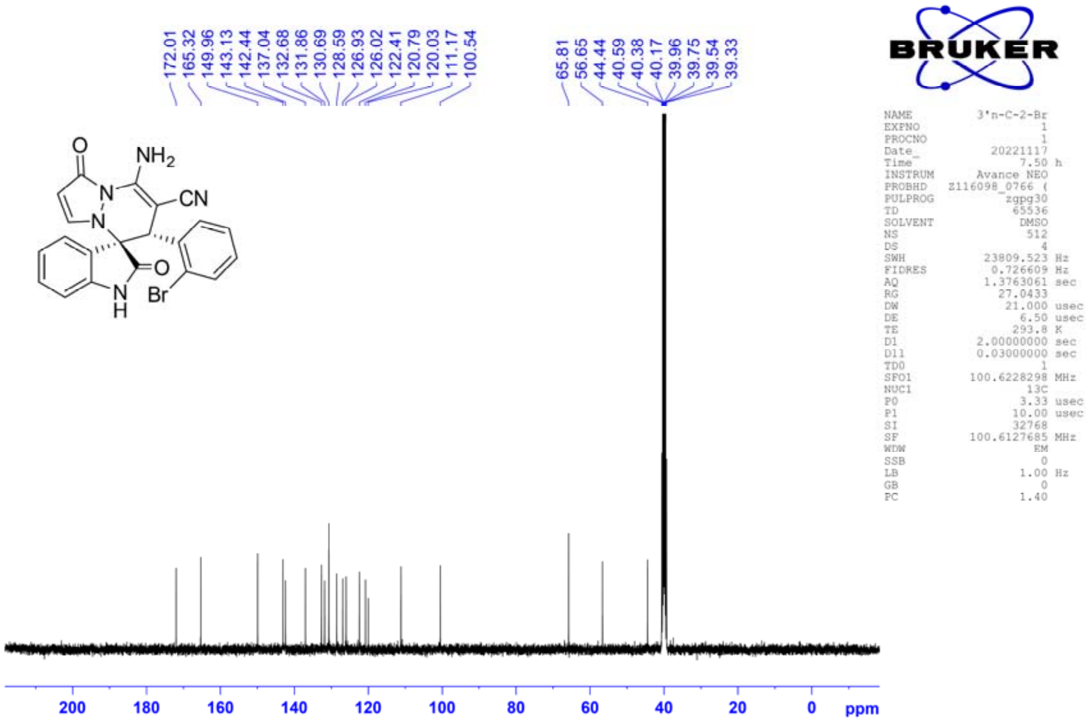

^{1}H and ^{13}C NMR Spectra for Compound **3o**

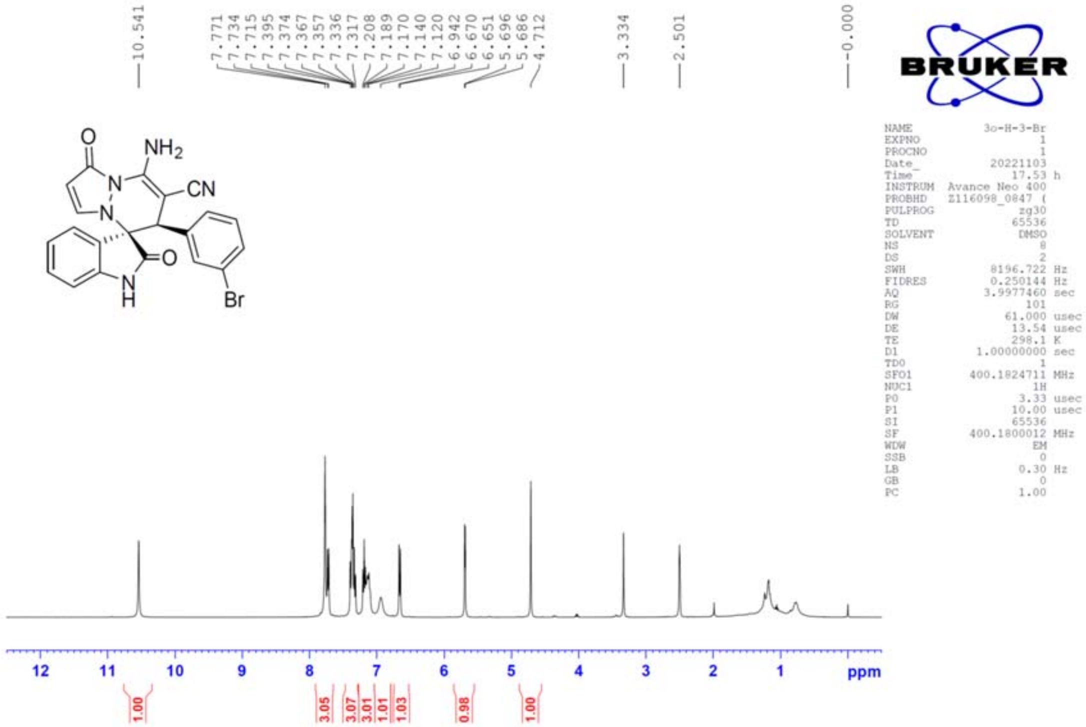

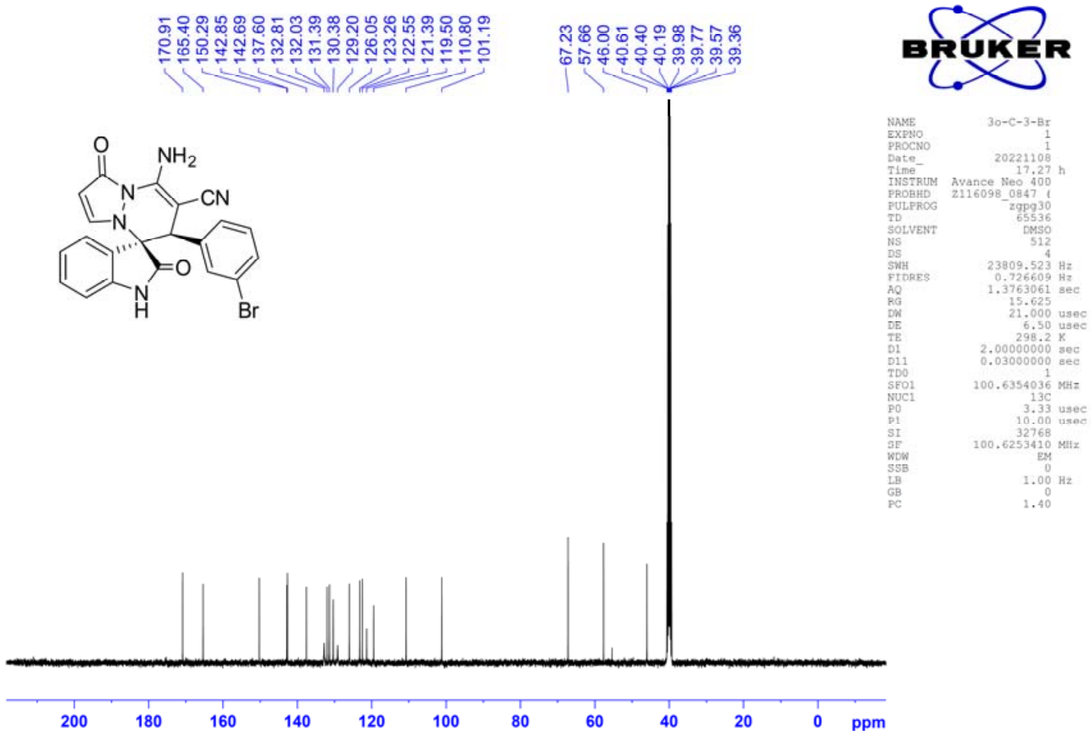

^{1}H and ^{13}C NMR Spectra for Compound **3’p**

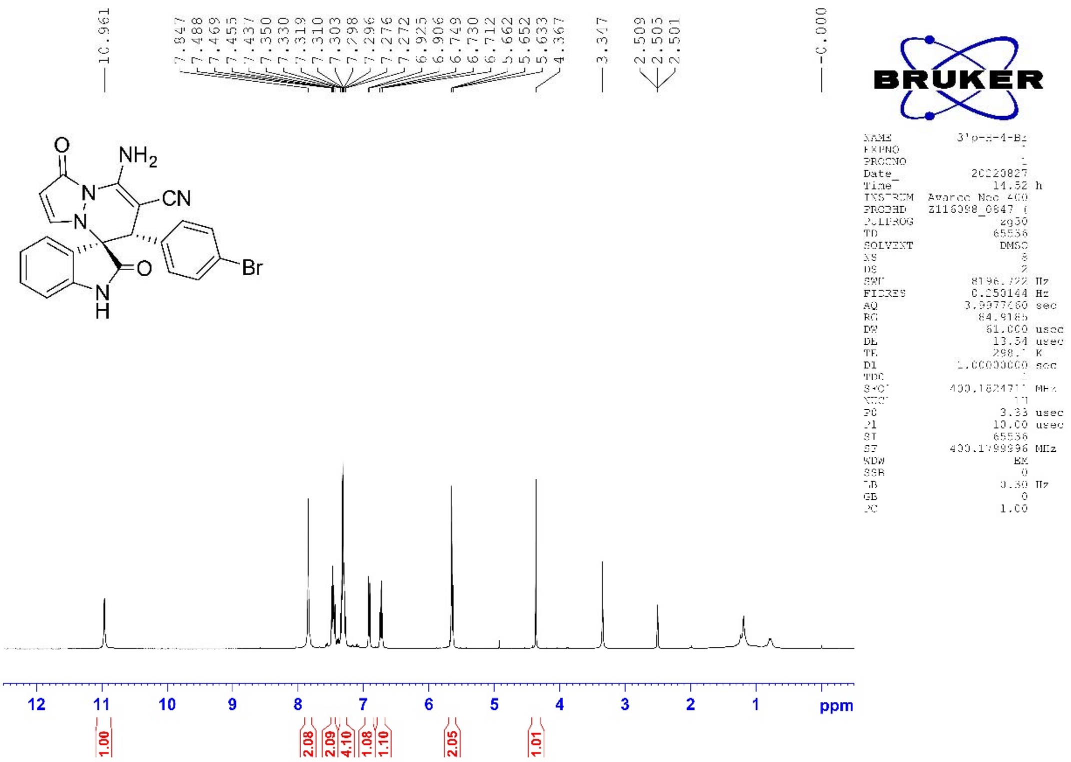

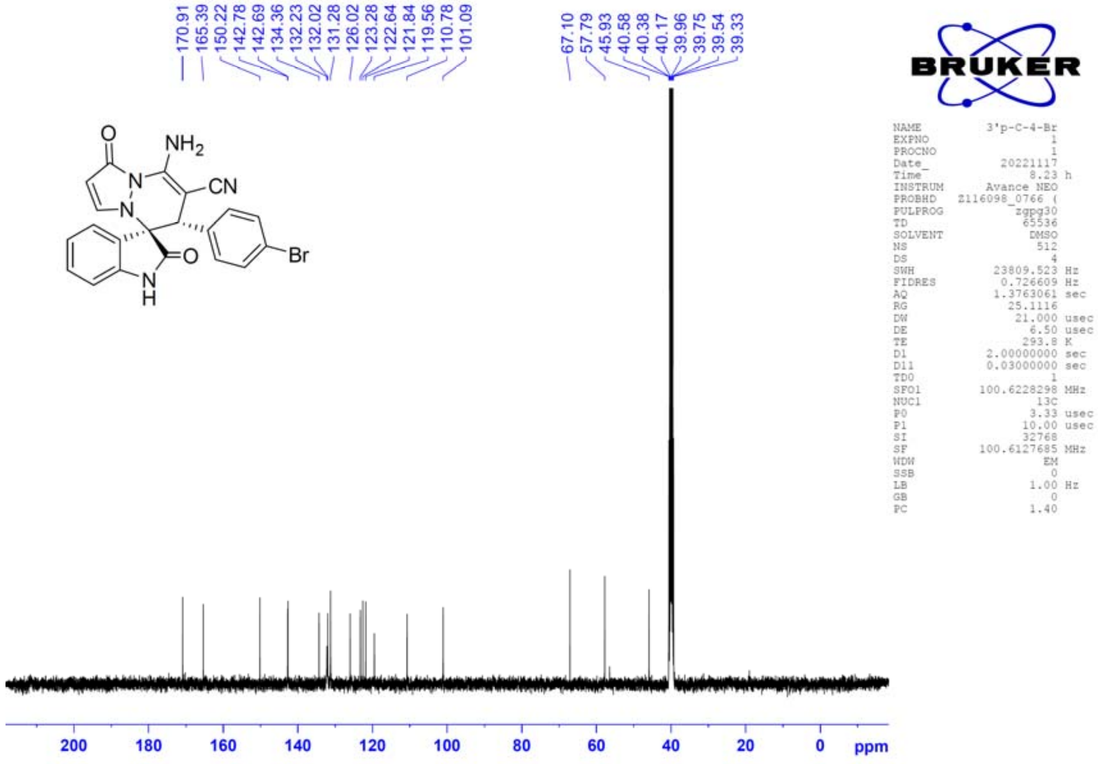

^{1}H and ^{13}C NMR Spectra for Compound **3q+3’q**

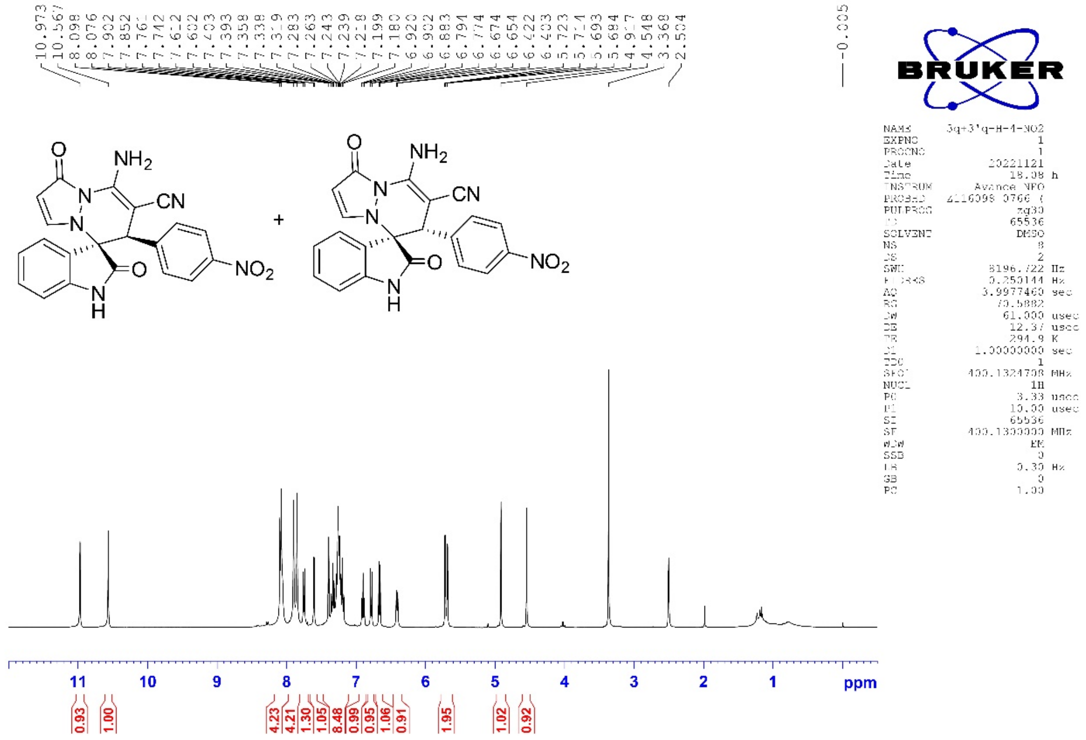

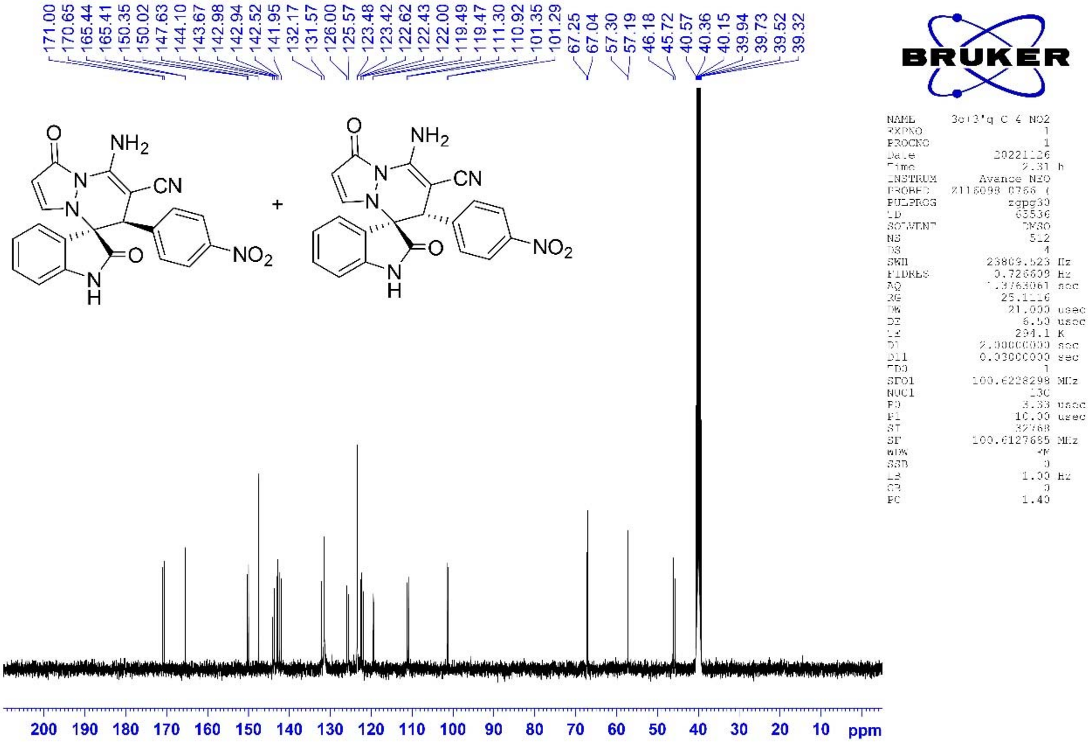

^{1}H and ^{13}C NMR Spectra for Compound **3r** (recrystallized from ethanol)

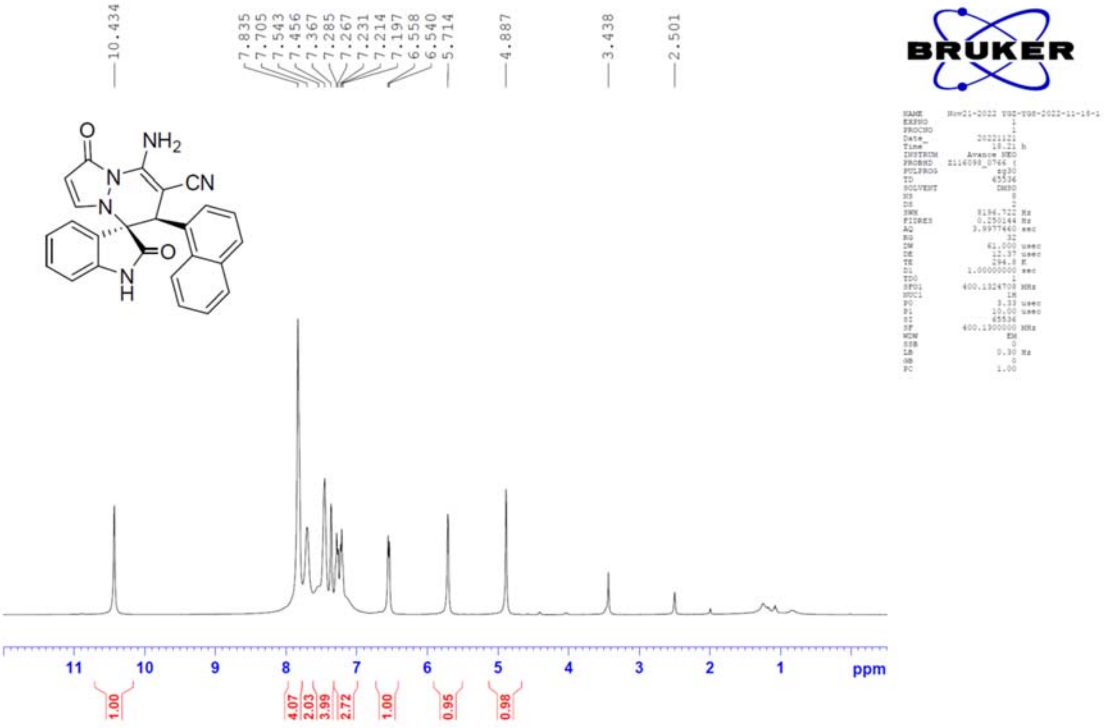

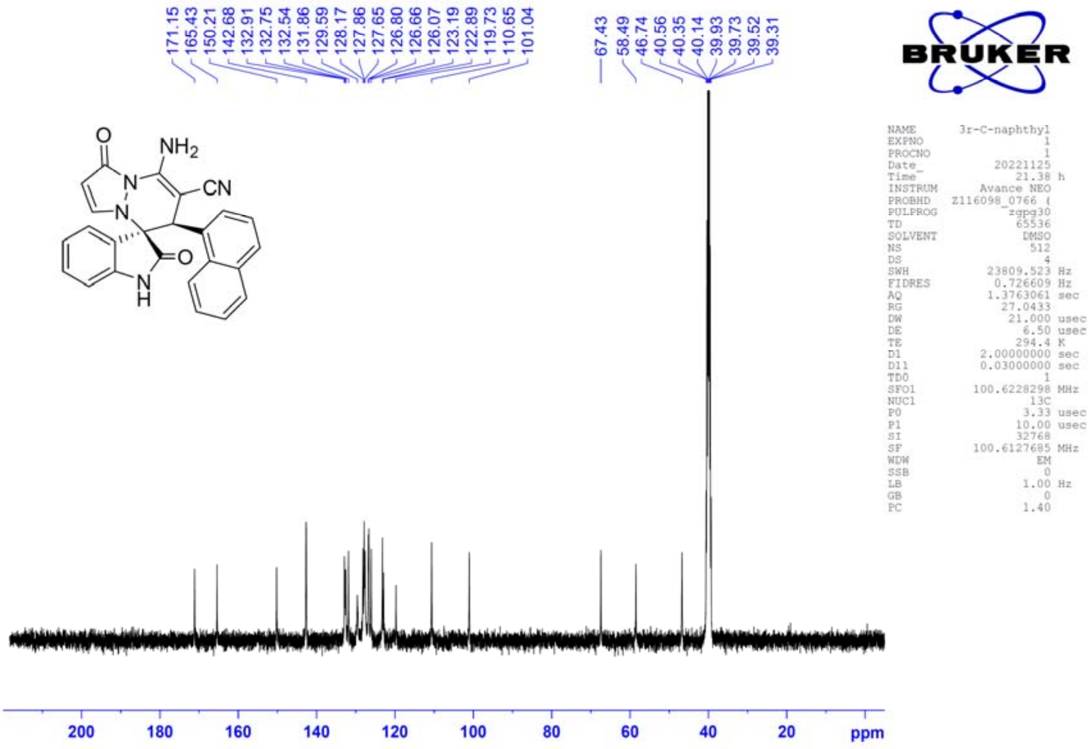

^{1}H and ^{13}C NMR Spectra for Compound **3s** (recrystallized from ethanol)

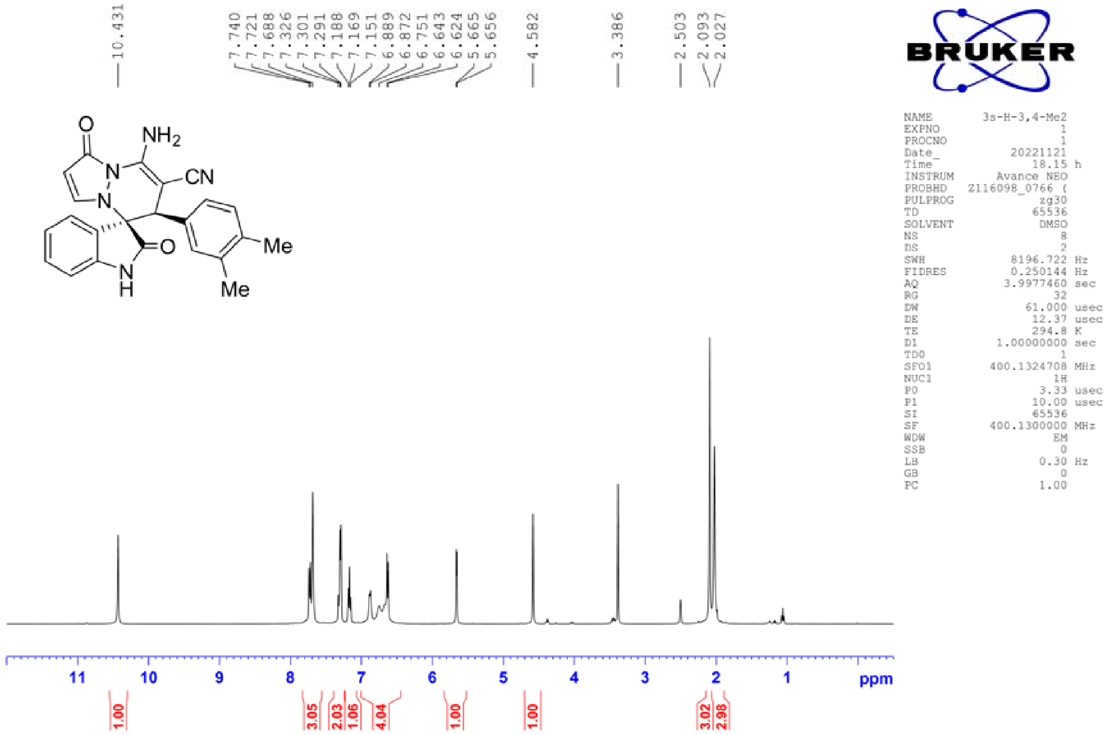

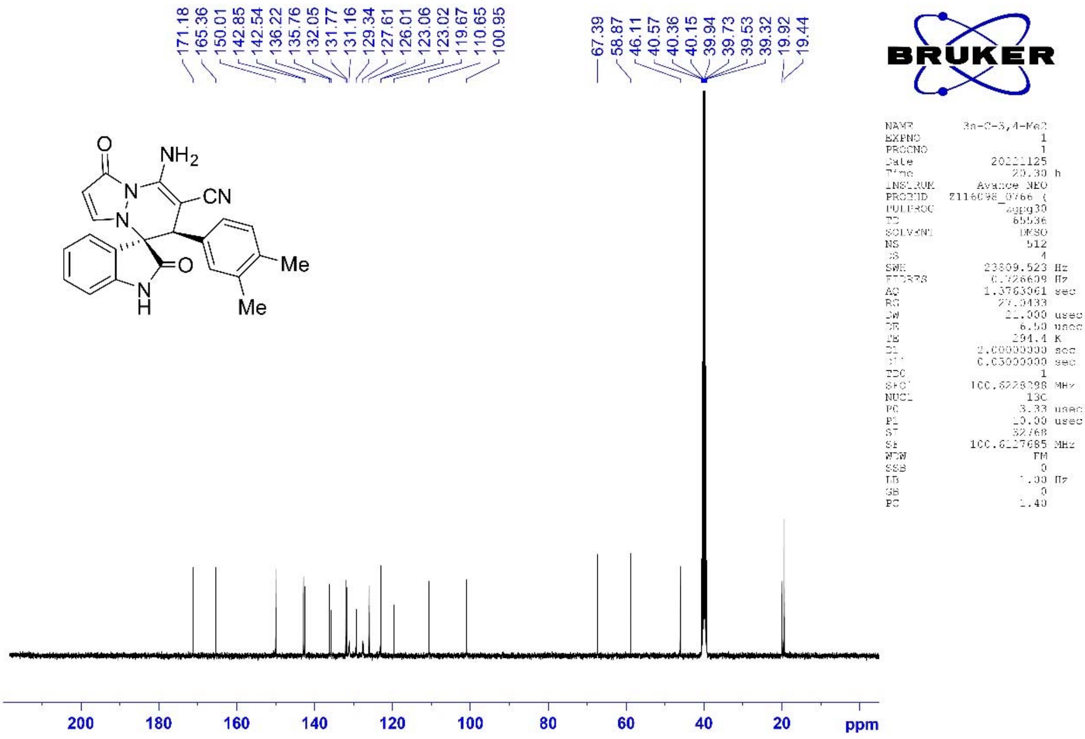

^{1}H and ^{13}C NMR Spectra for Compound **3’t**

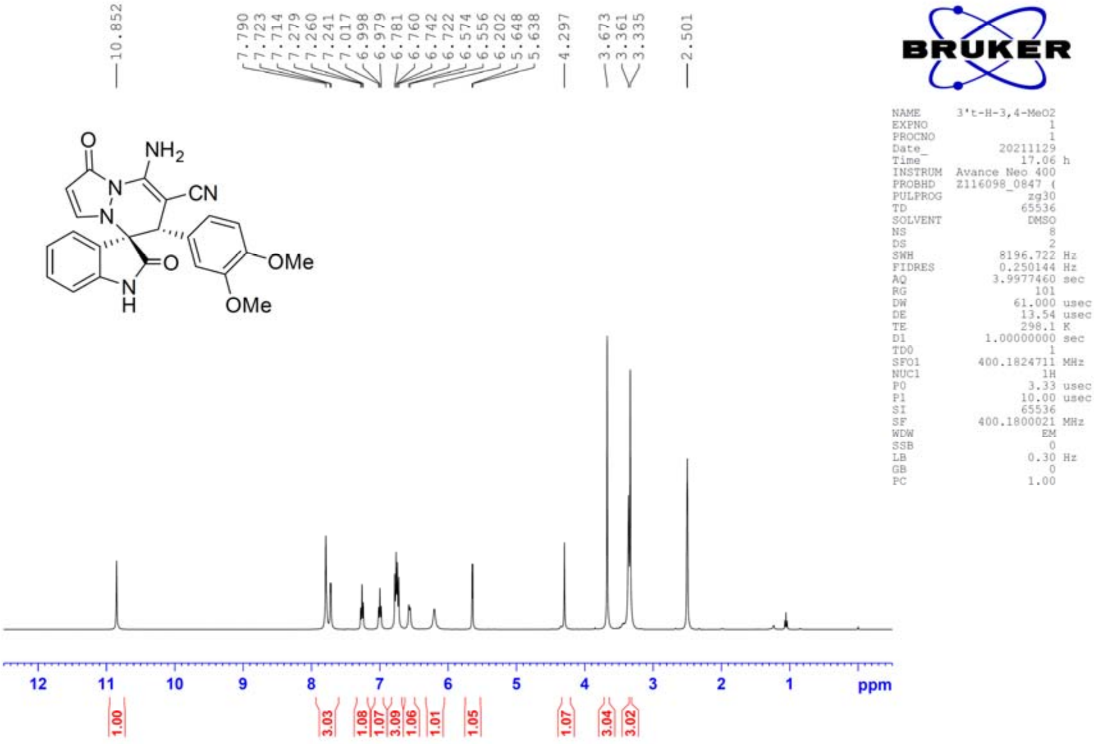

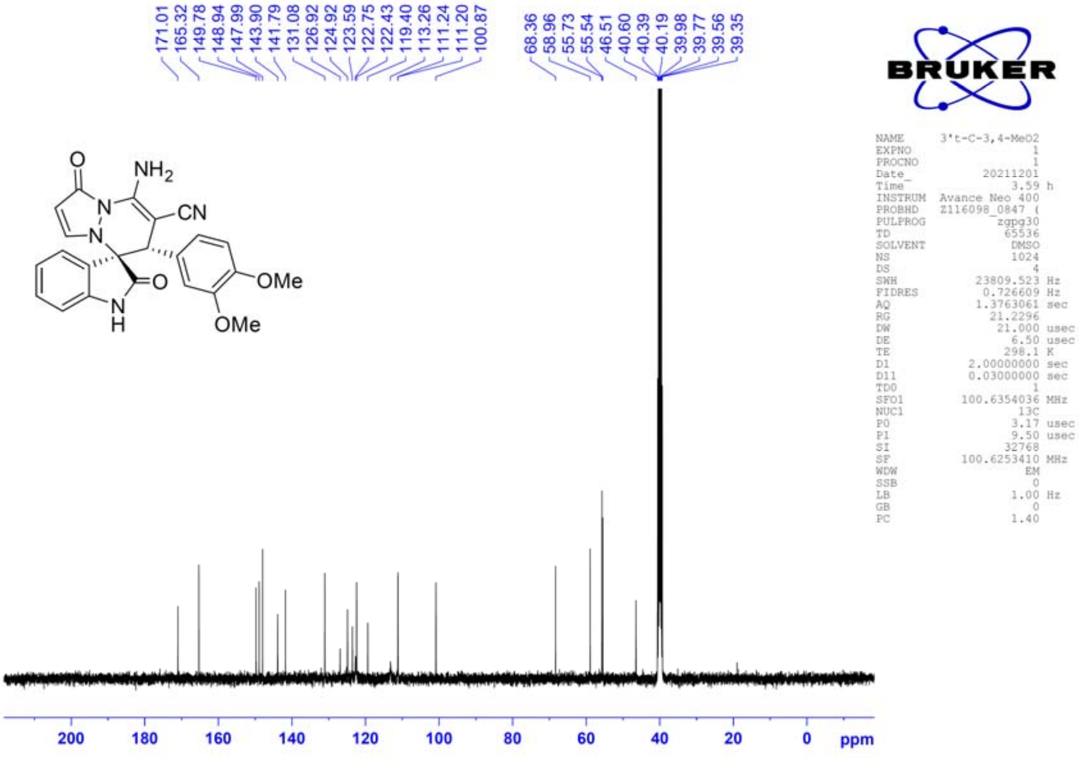

^{1}H and ^{13}C NMR Spectra for Compound **3u+3'u**

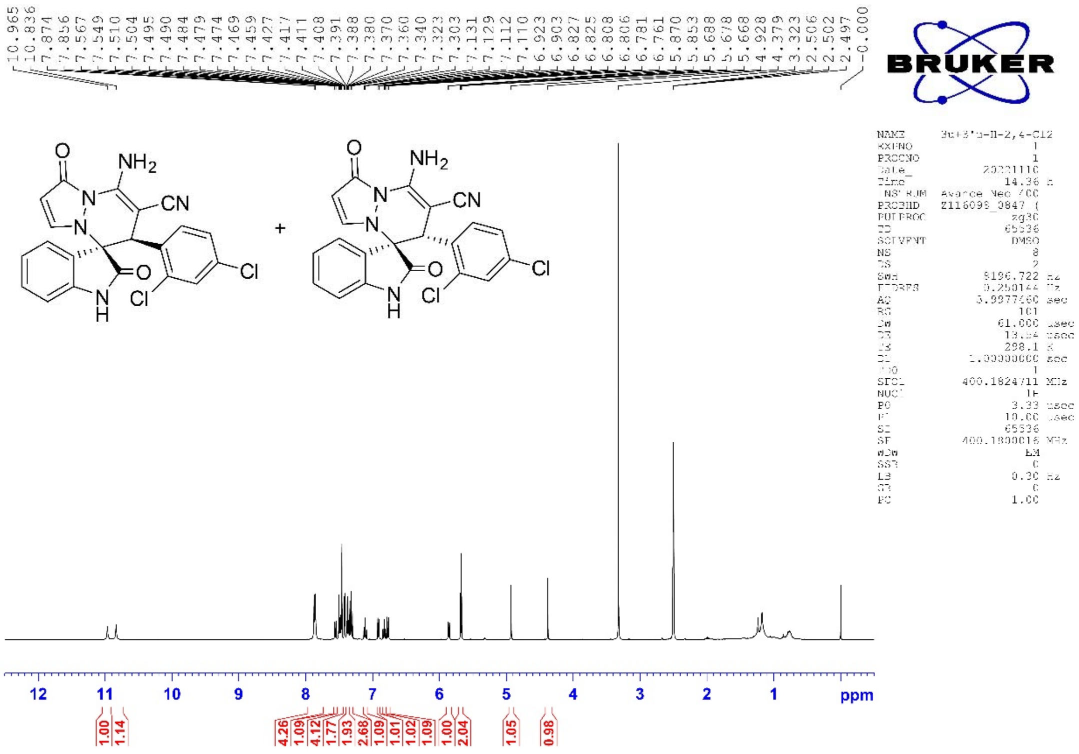

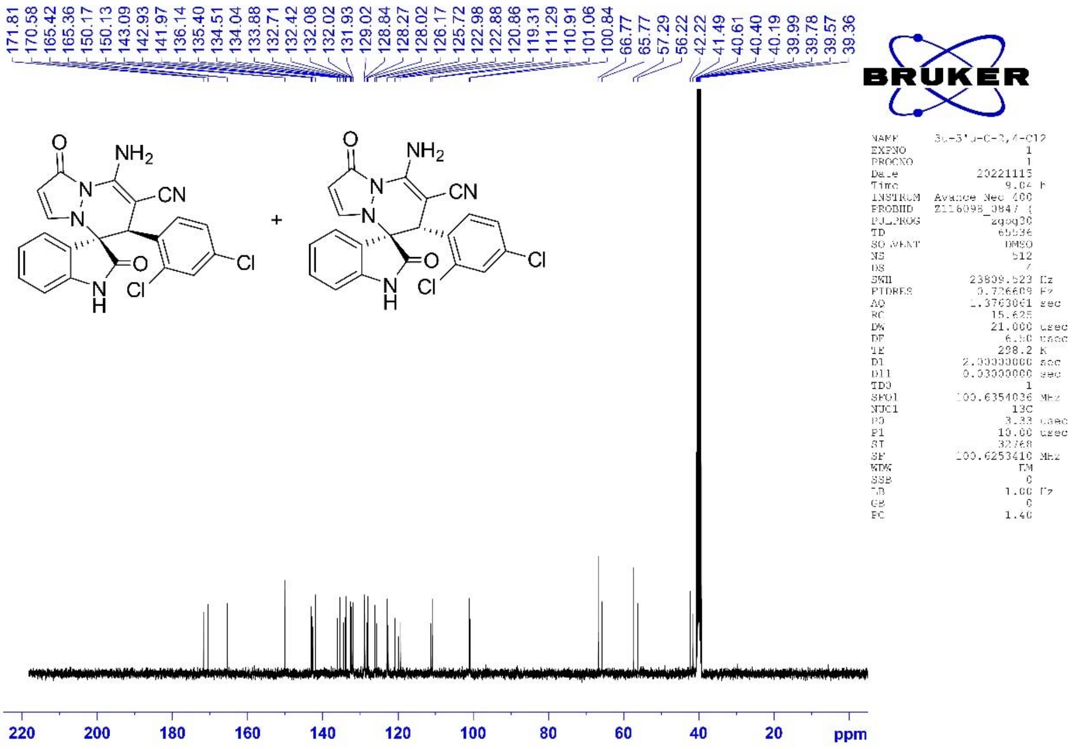

^1H and ^{13}C NMR Spectra for Compound **3v** (recrystallized from ethanol)碳谱差 2 个峰

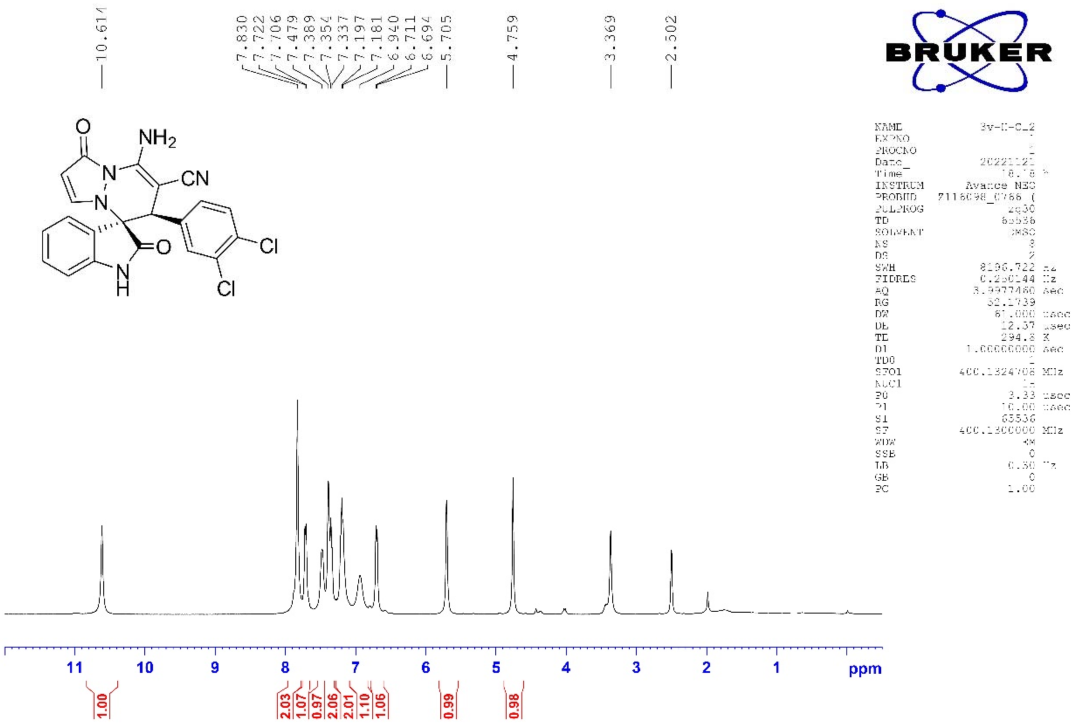

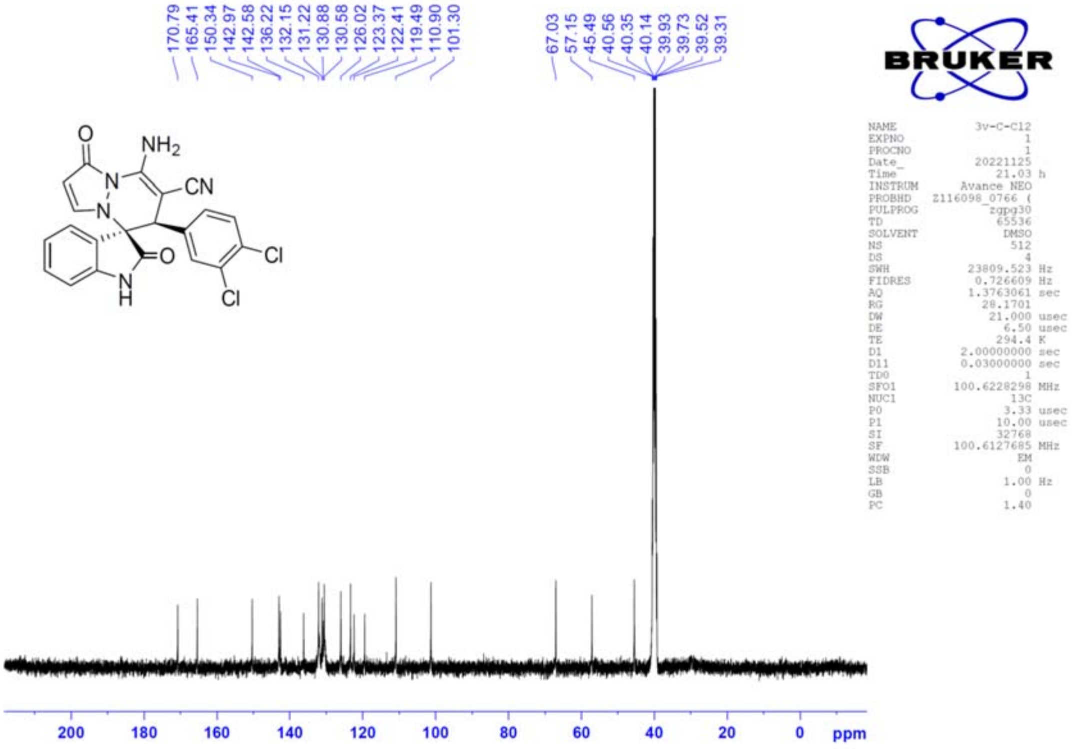

^1H and ^{13}C NMR Spectra for Compound **3w/3'w**

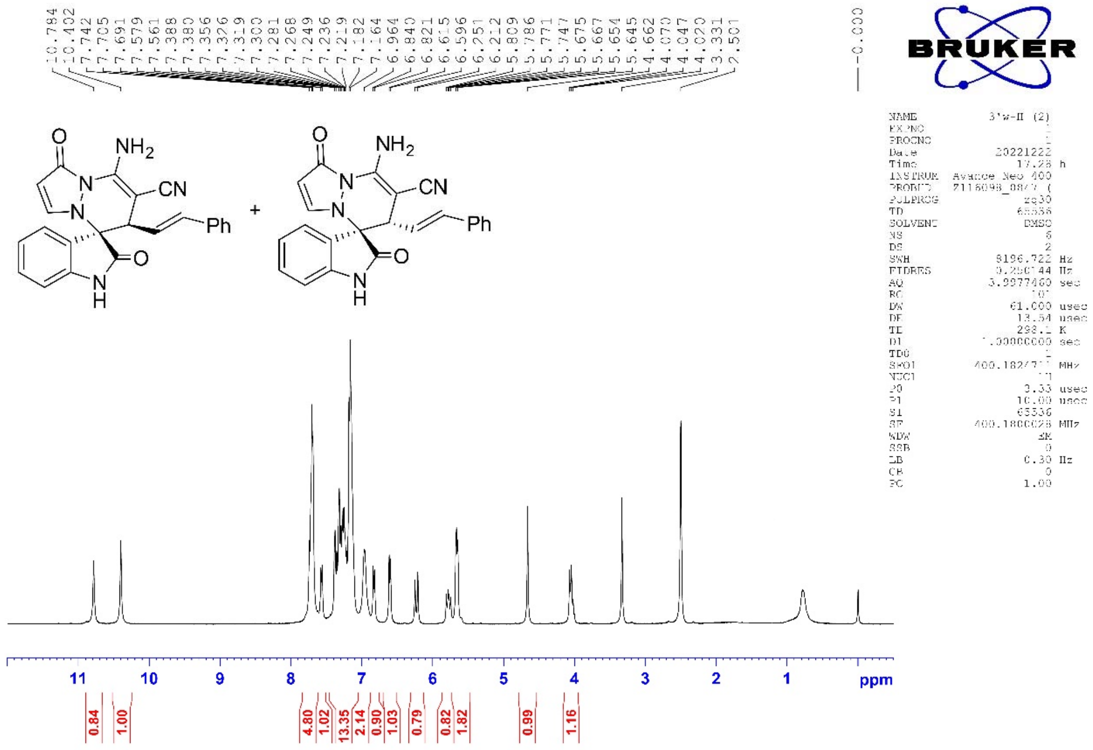

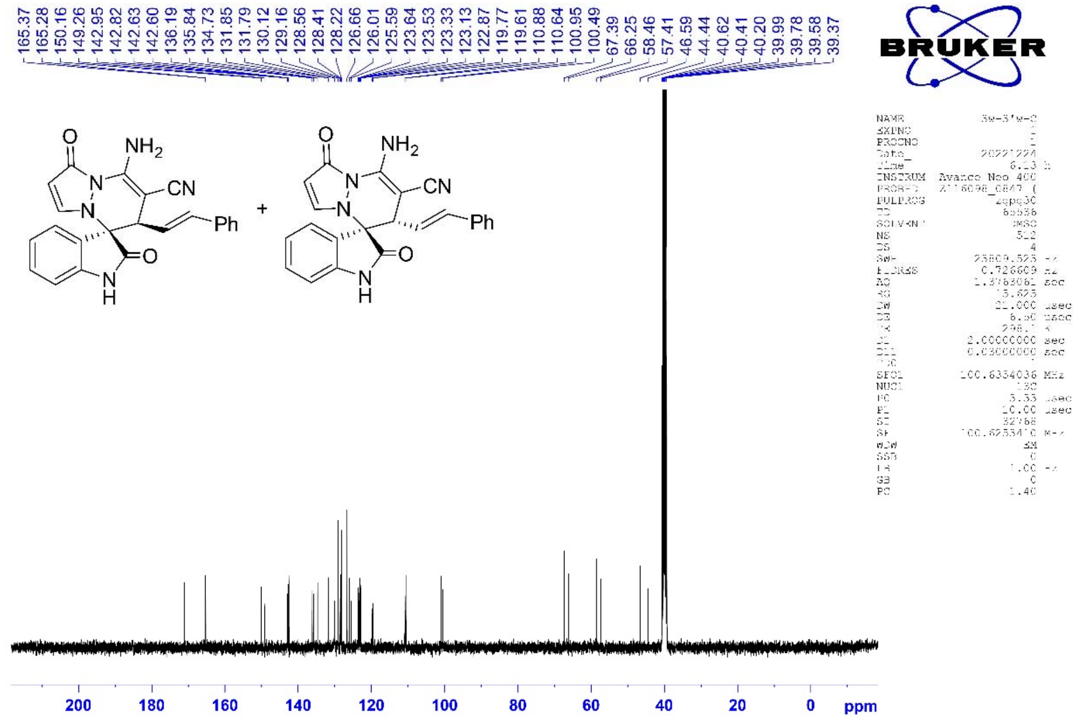

^{1}H and ^{13}C NMR Spectra for Compound **4a** (recrystallized from ethanol)

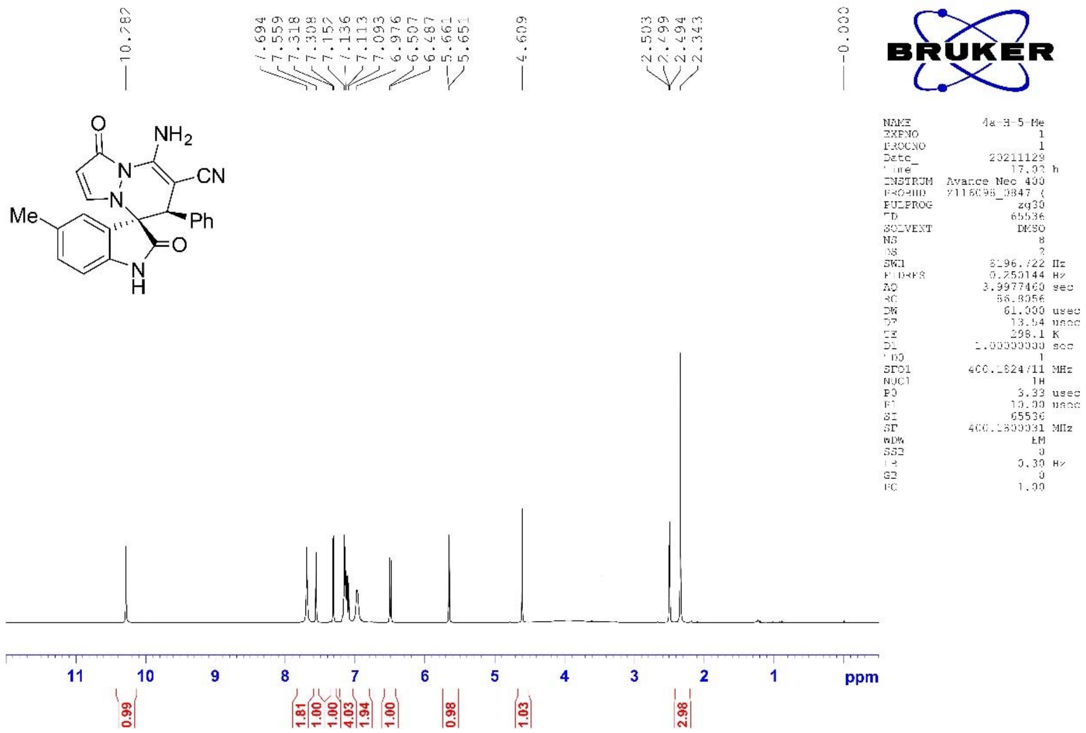

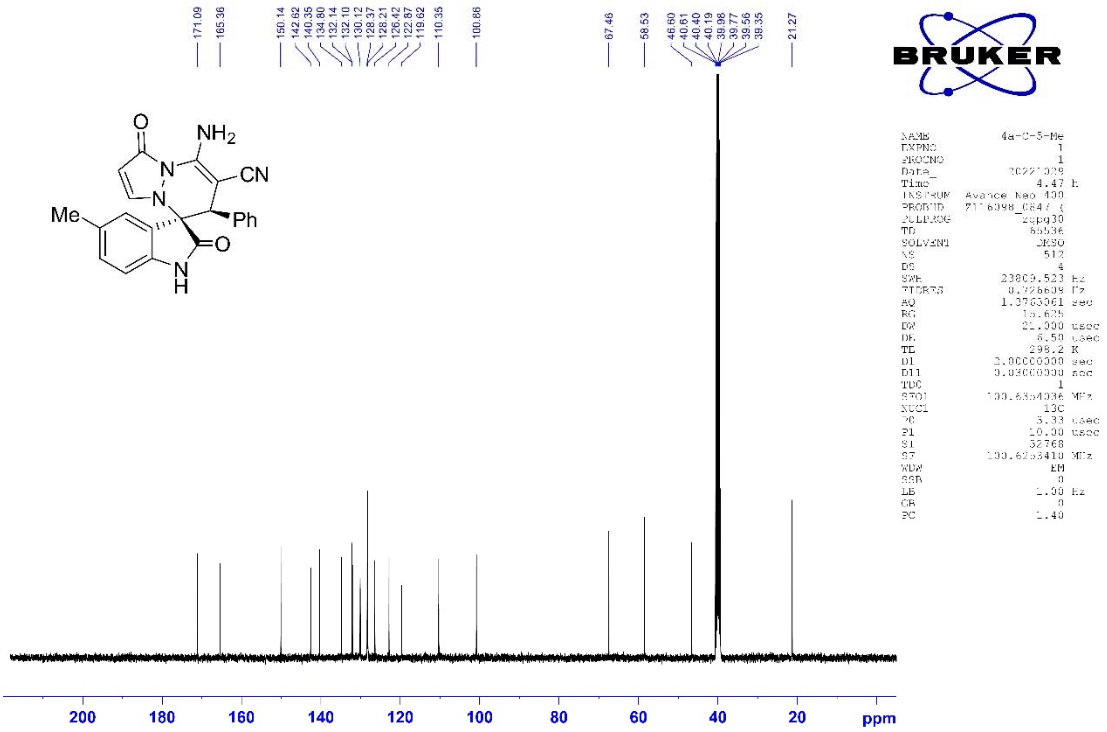

^1H and ^{13}C NMR Spectra for Compound **4b/4’b** (recrystallized from ethanol)

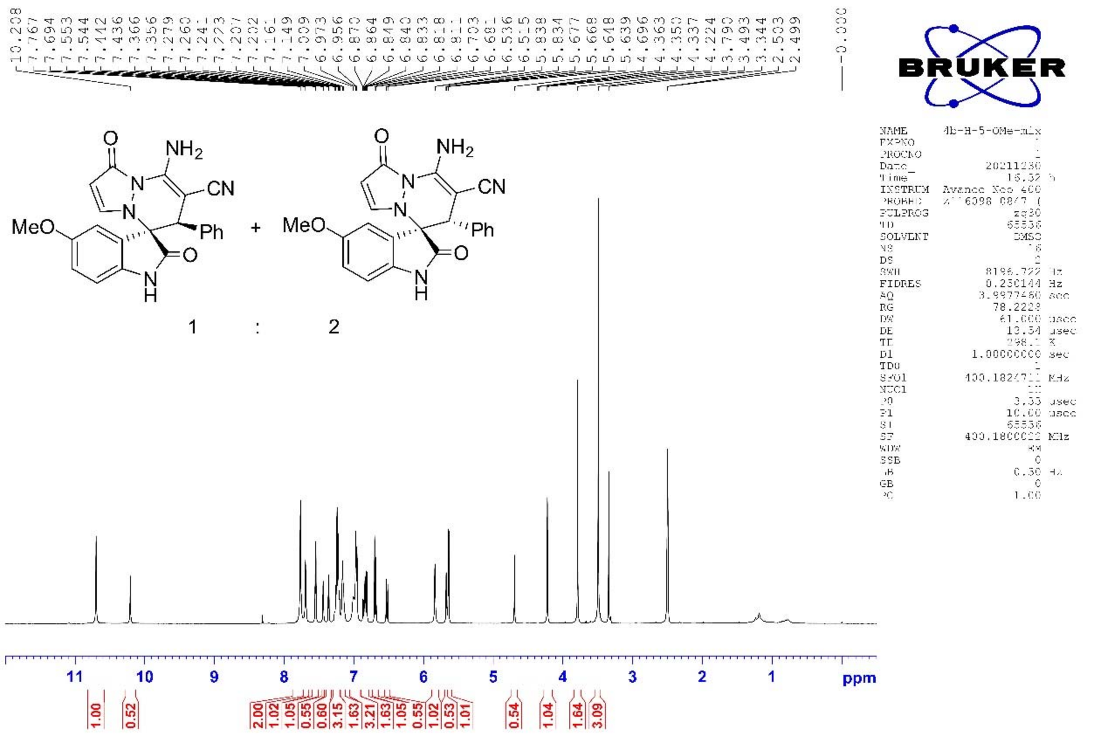

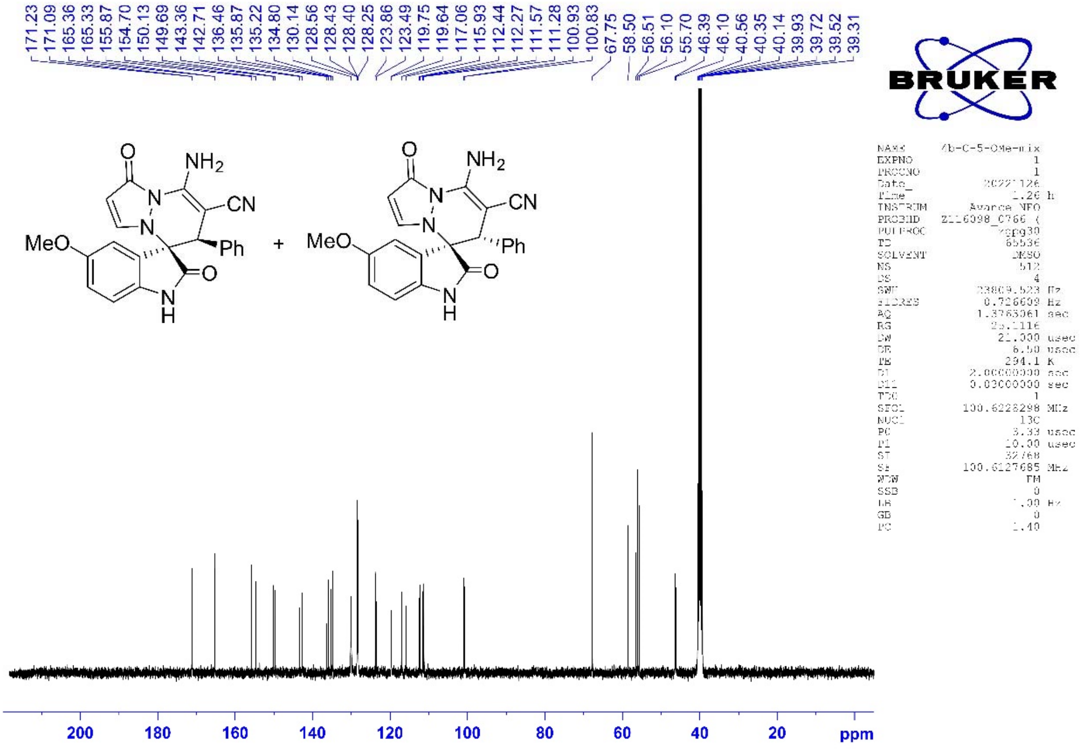

^{1}H and ^{13}C NMR Spectra for Compound **4c** (recrystallized from ethanol)

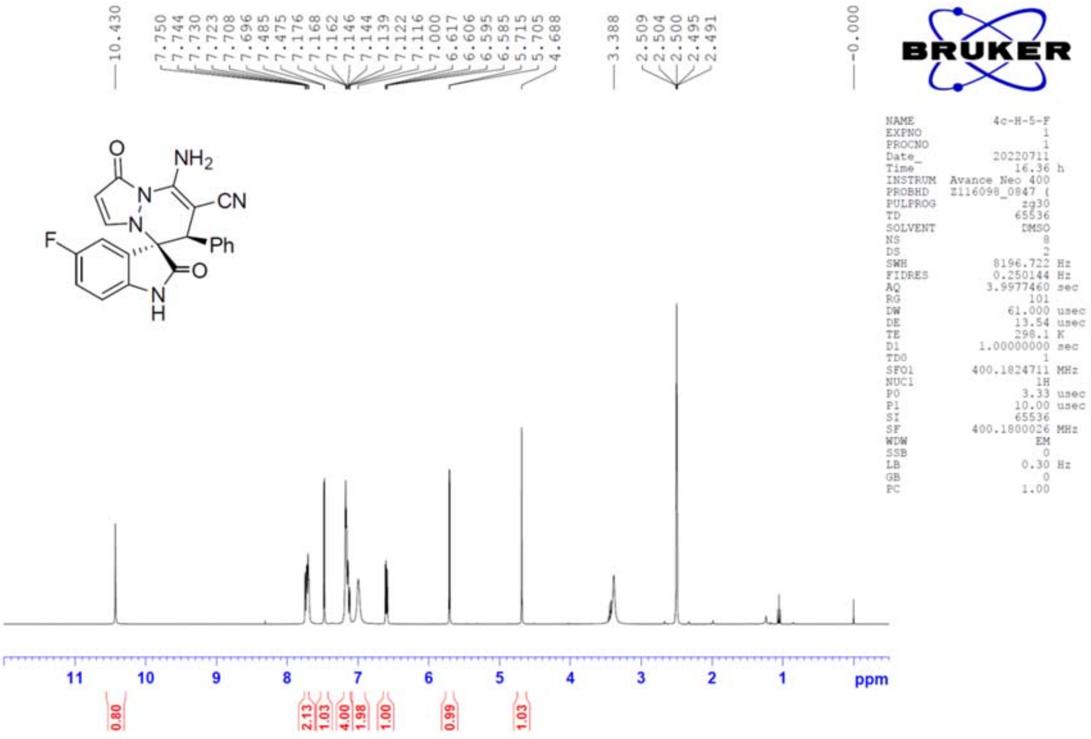

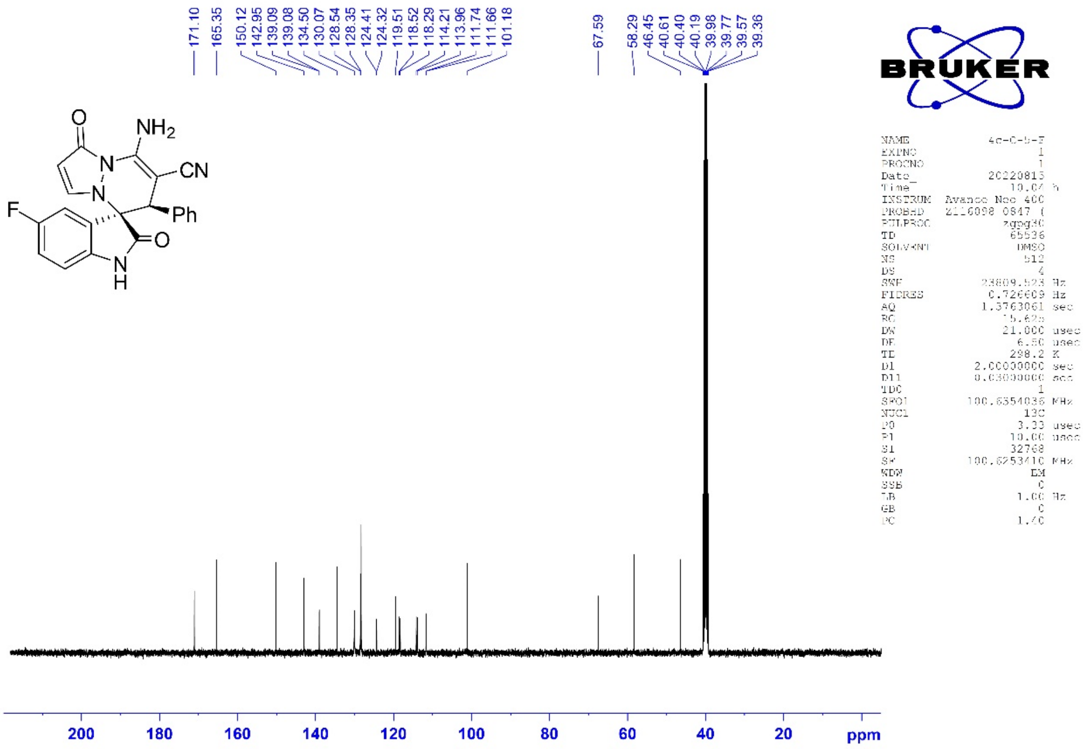
171.10
165.35
150.12
142.95
139.09
139.08
134.50
130.07
128.54
128.35
124.41
124.32
119.51
118.52
118.29
114.21
113.96
111.74
111.66
101.18
67.59
58.29
46.45
40.61
40.40
40.19
39.98
39.77
39.57
39.36
O
NH2
N
CN
N
F
Ph
O
N
H
200
180
160
140
120
100
80
60
40
20
ppm

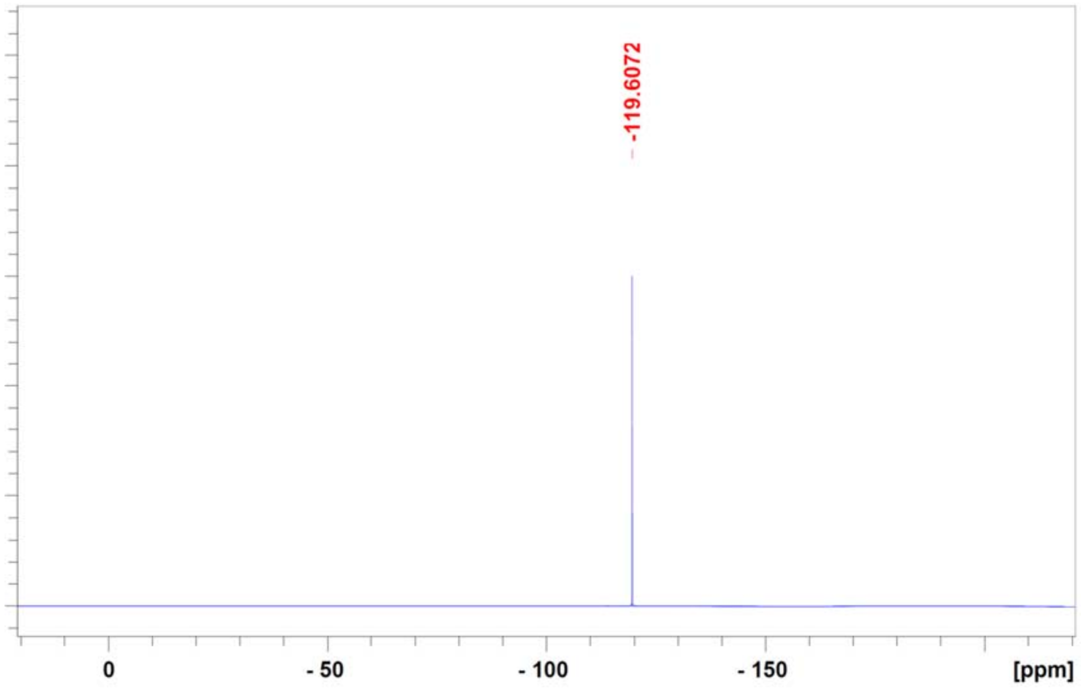
-119.6072
0
- 50
- 100
- 150
[ppm]

^{1}H and ^{13}C NMR Spectra for Compound **4d** (recrystallized from ethanol)

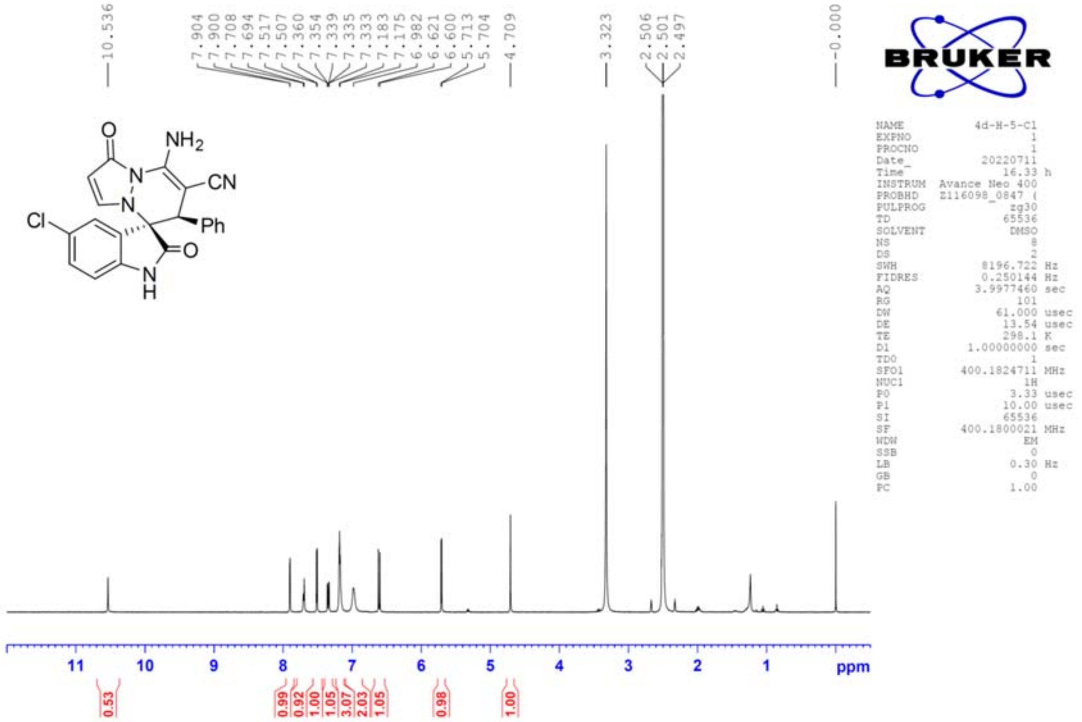

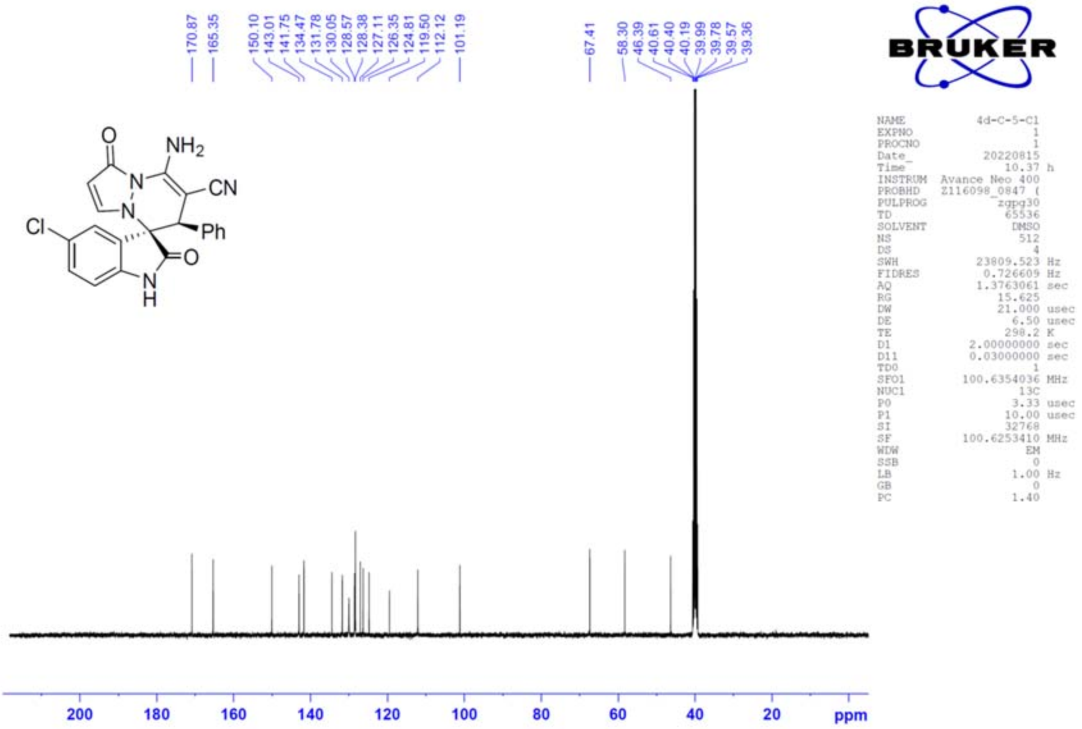

^{1}H and ^{13}C NMR Spectra for Compound **4e** (recrystallized from ethanol)

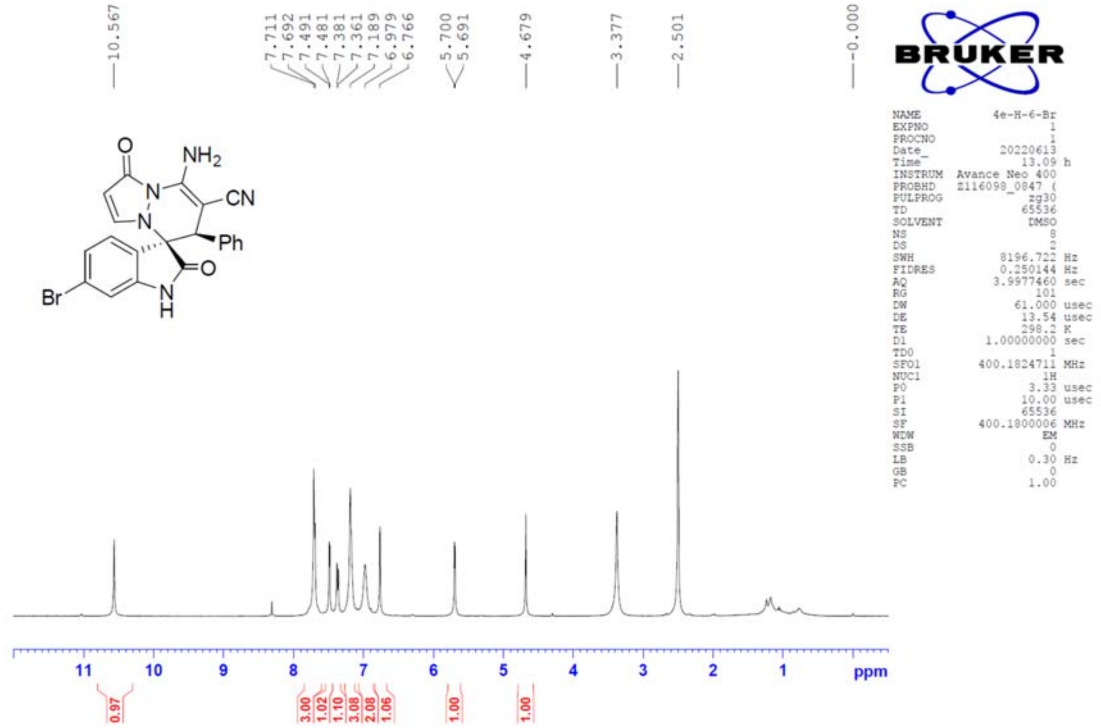

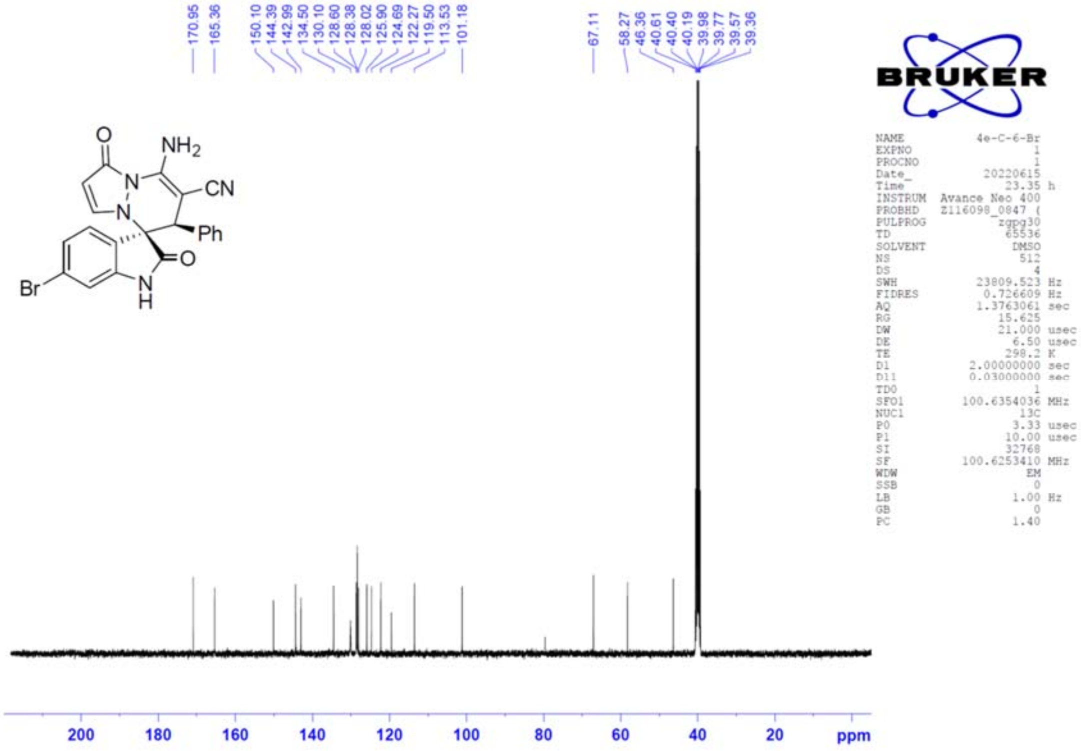

^{1}H and ^{13}C NMR Spectra for Compound **4f** (recrystallized from ethanol)

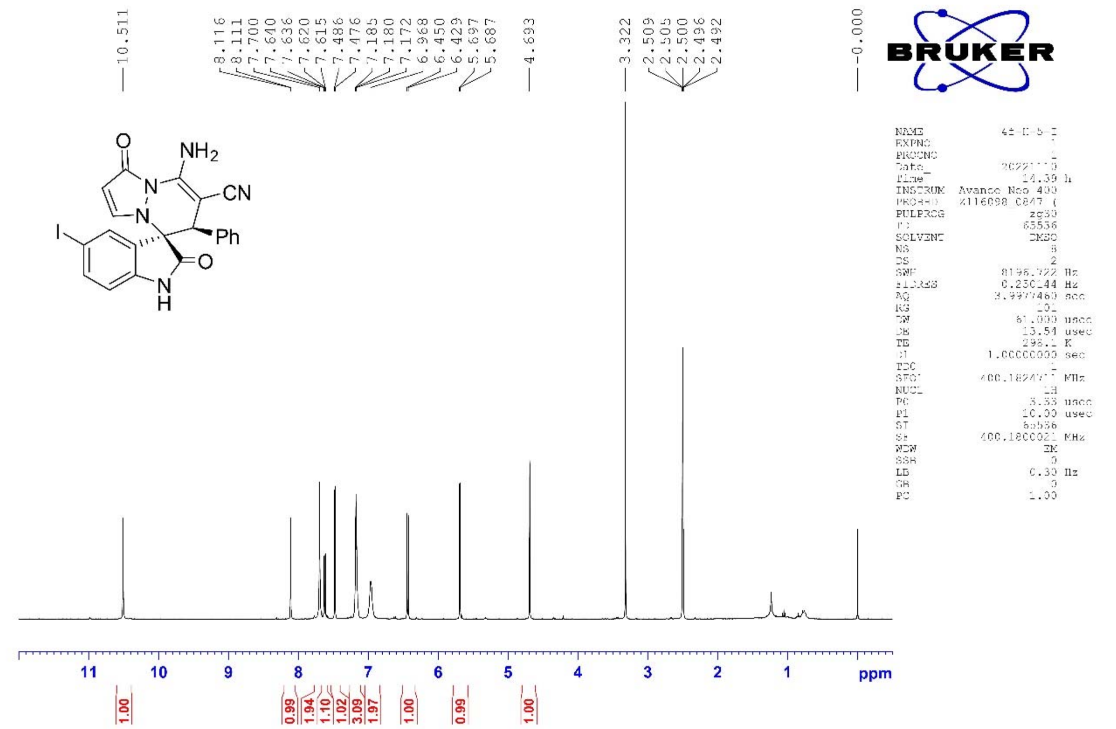

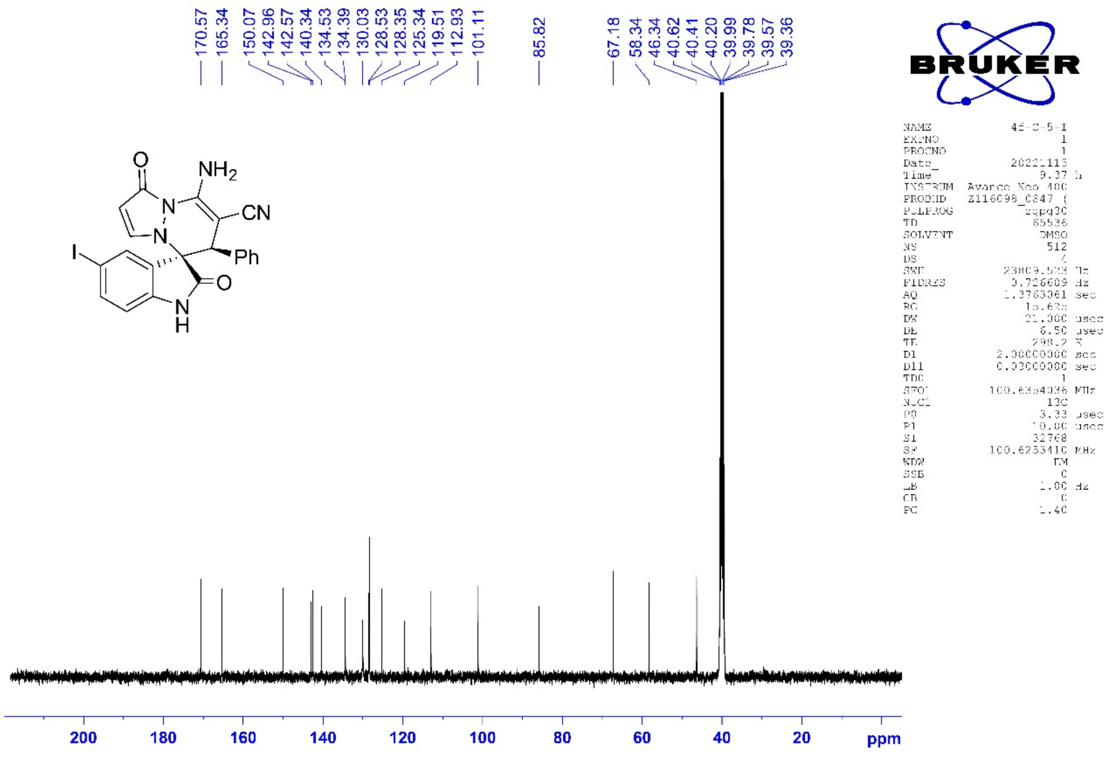

^{1}H and ^{13}C NMR Spectra for Compound **4g** (recrystallized from ethanol)

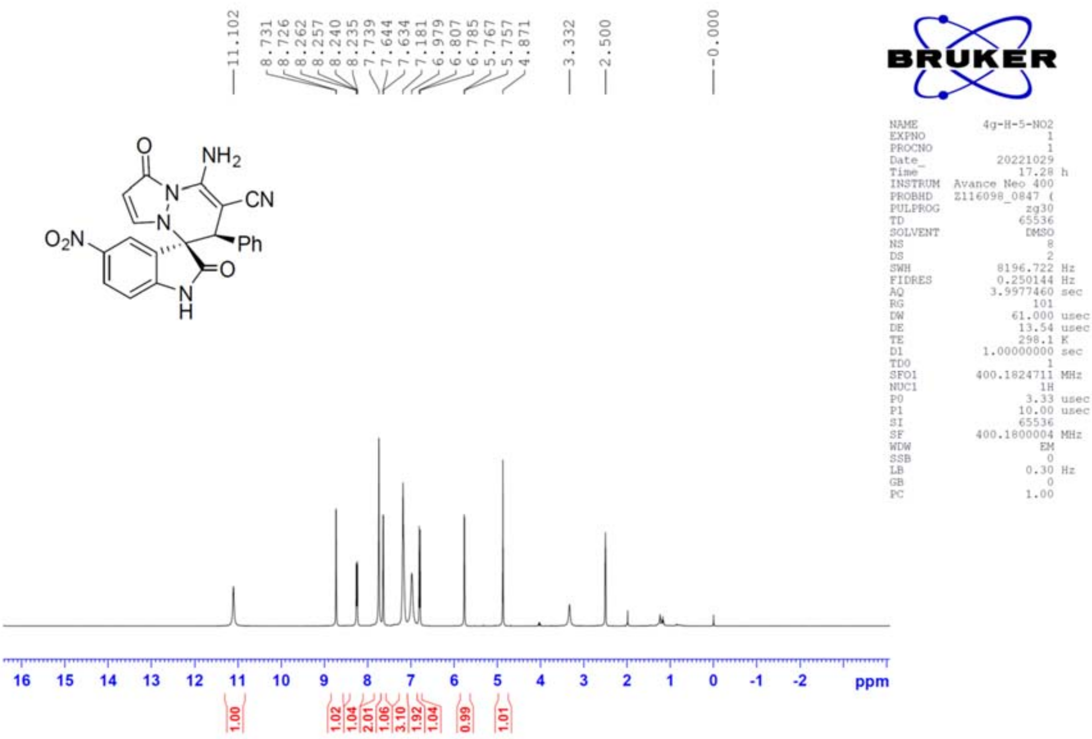

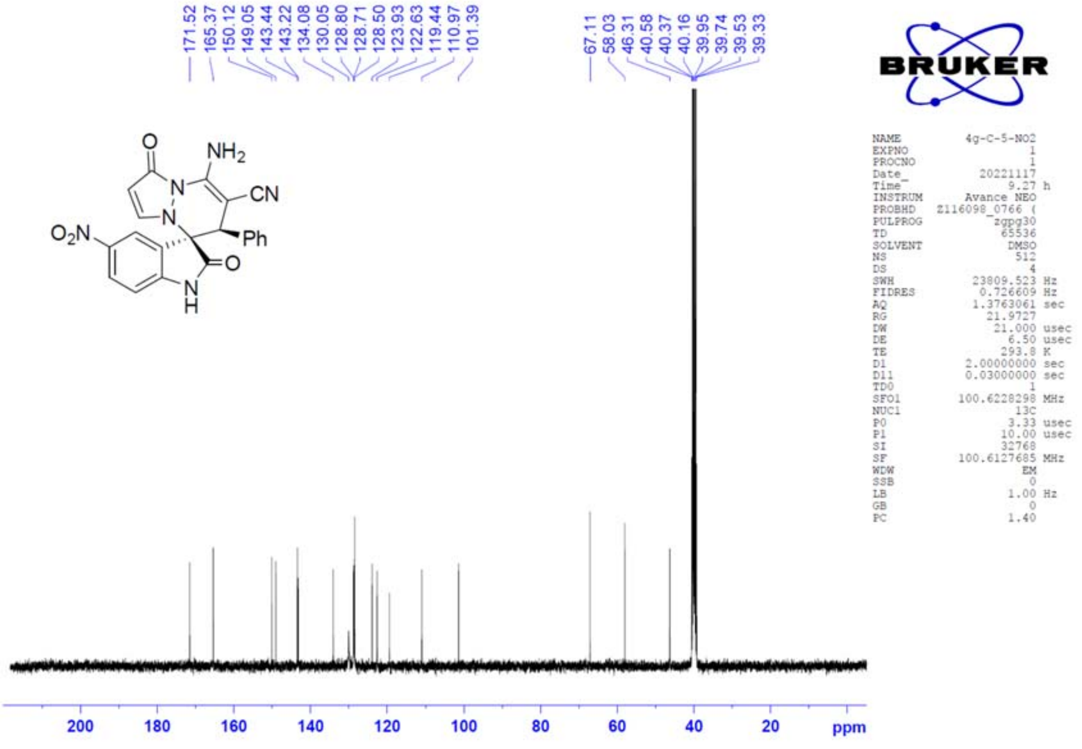

^{1}H and ^{13}C NMR Spectra for Compound **4'h/4h** (recrystallized from ethanol)需要重新核对

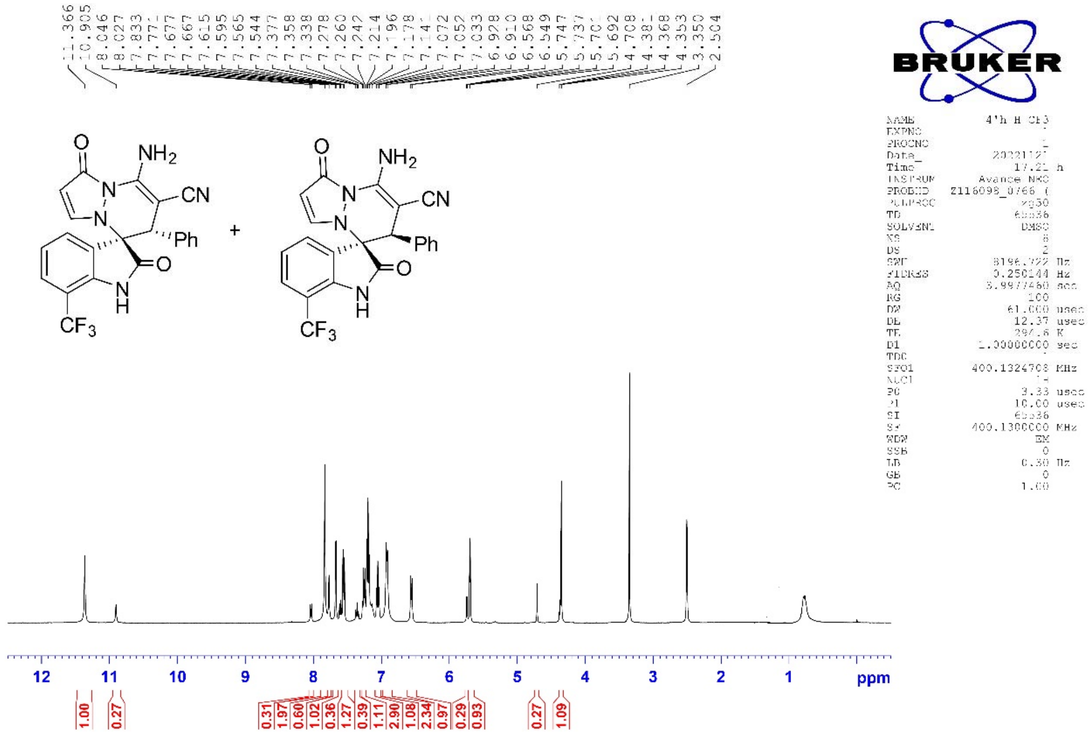

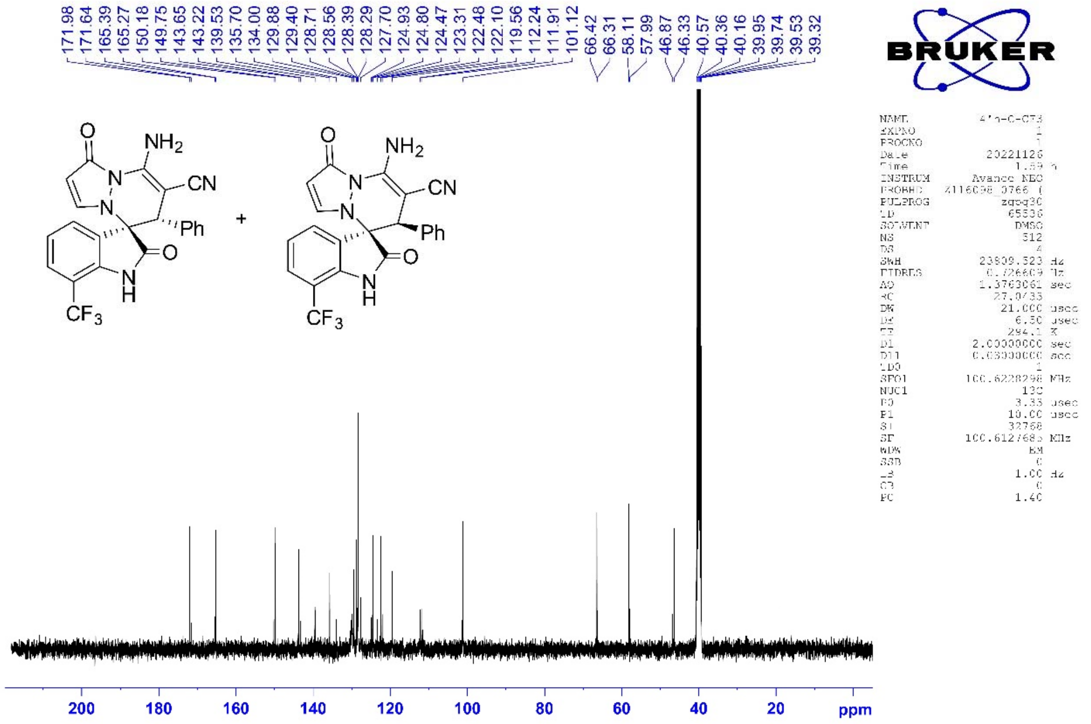

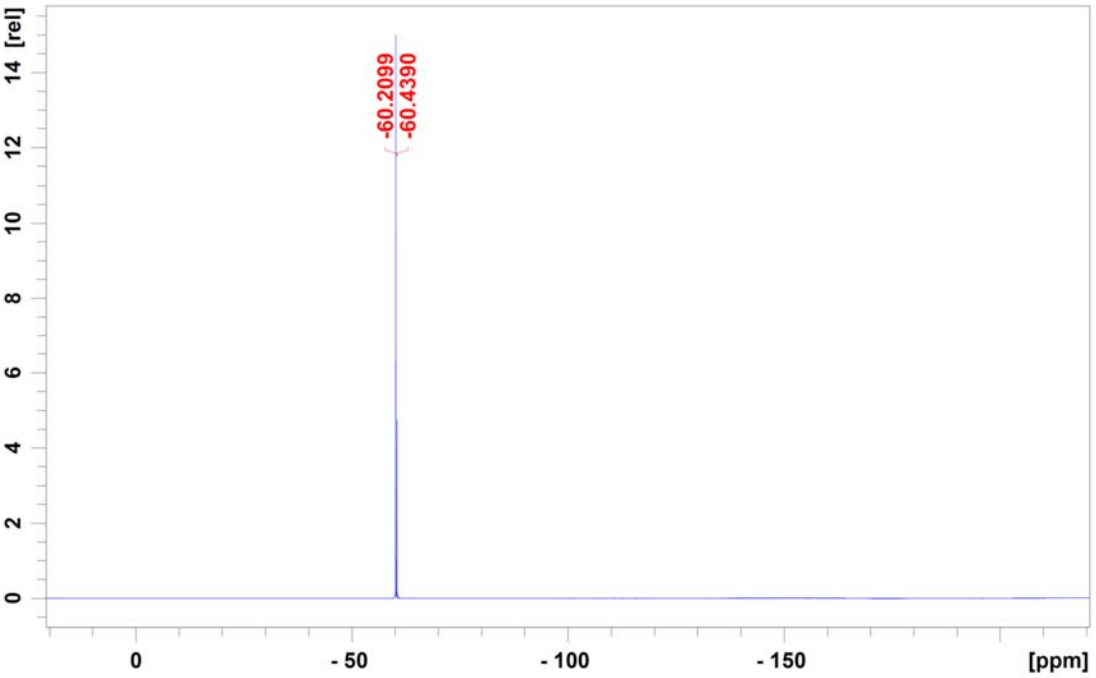

^{1}H and ^{13}C NMR Spectra for Compound **5a** (recrystallized from ethanol)

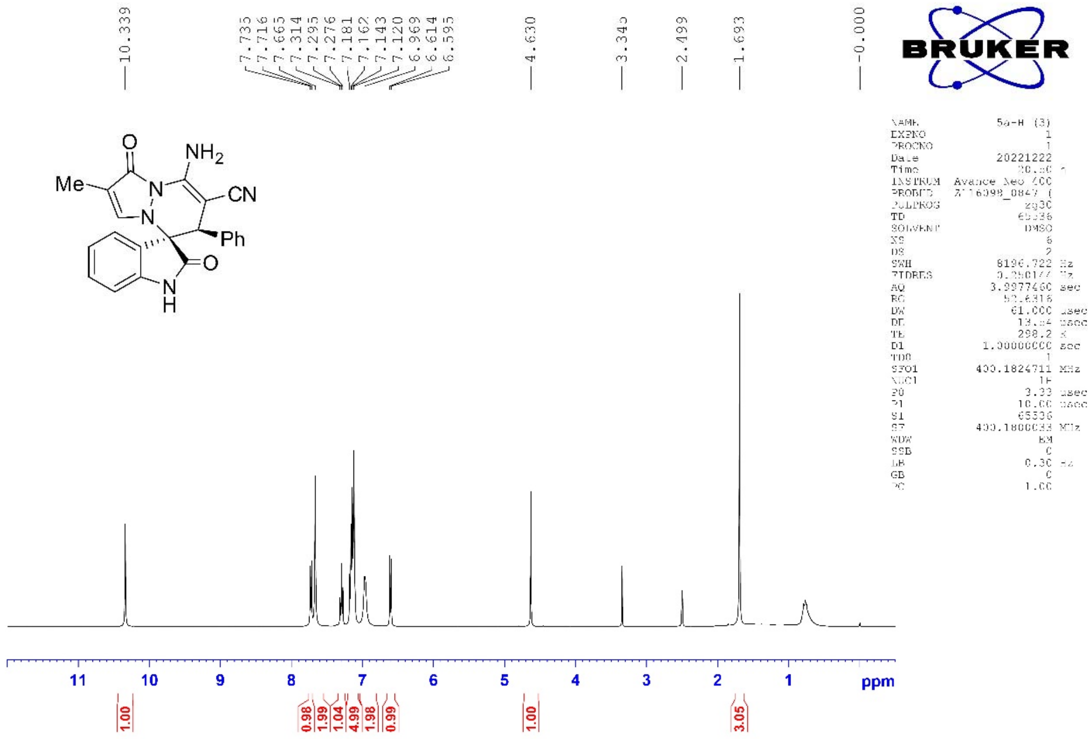

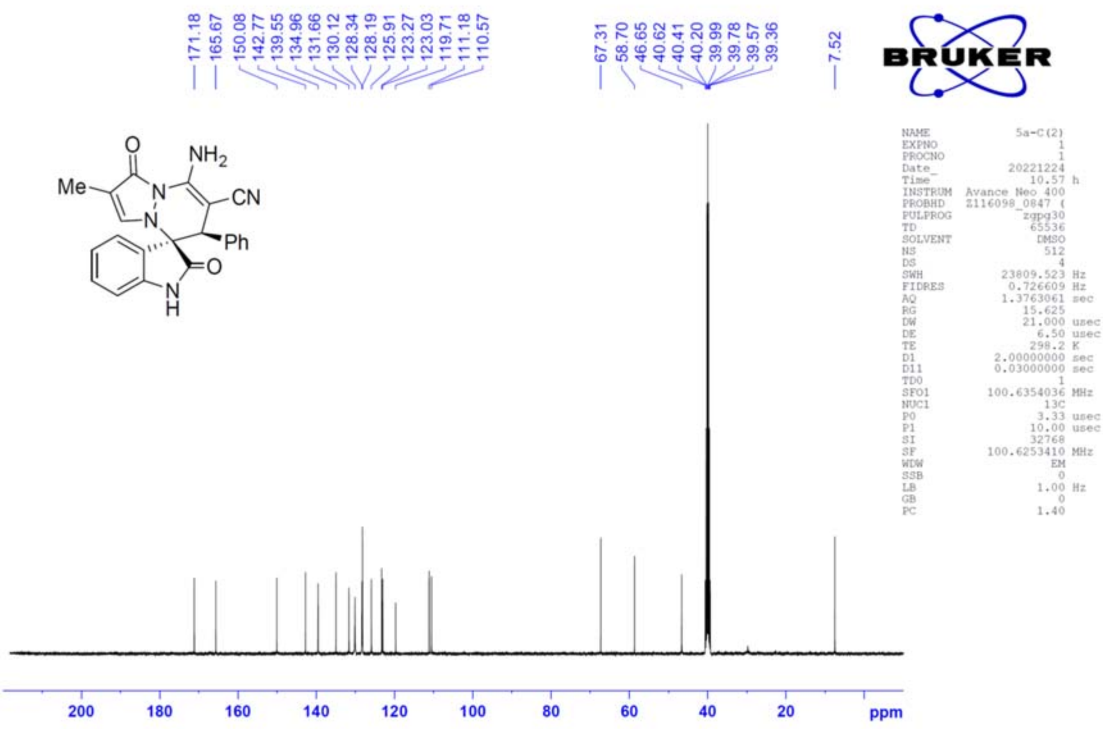

^{1}H and ^{13}C NMR Spectra for Compound **5′b+5b** (recrystallized from ethanol)

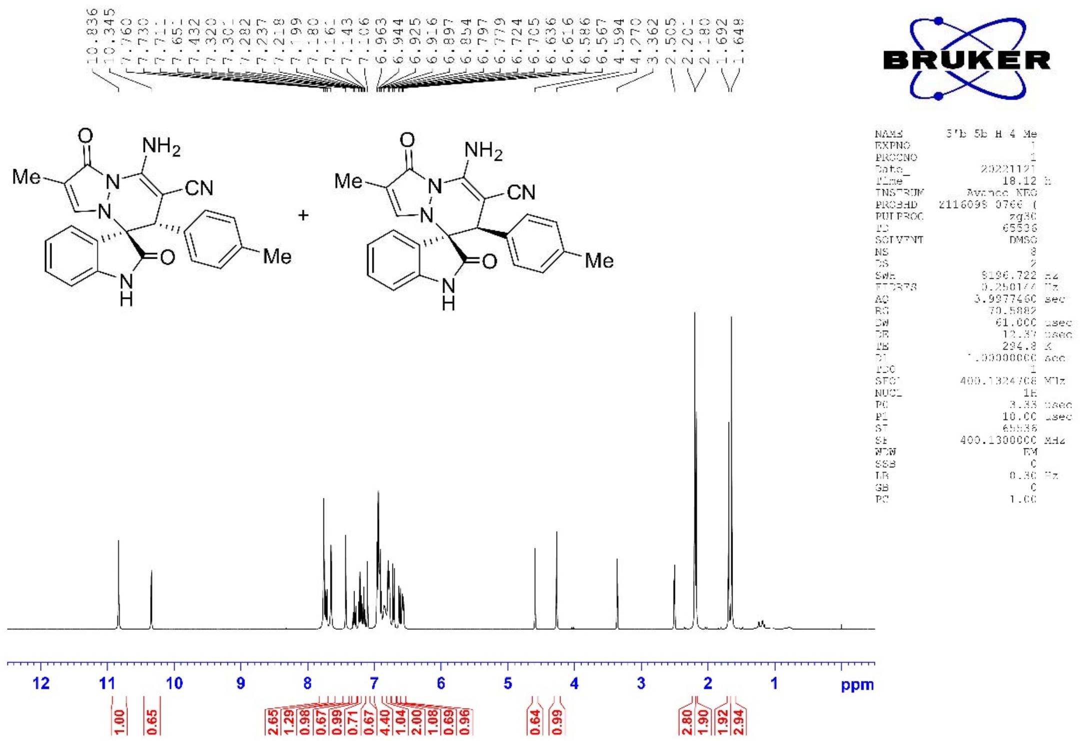

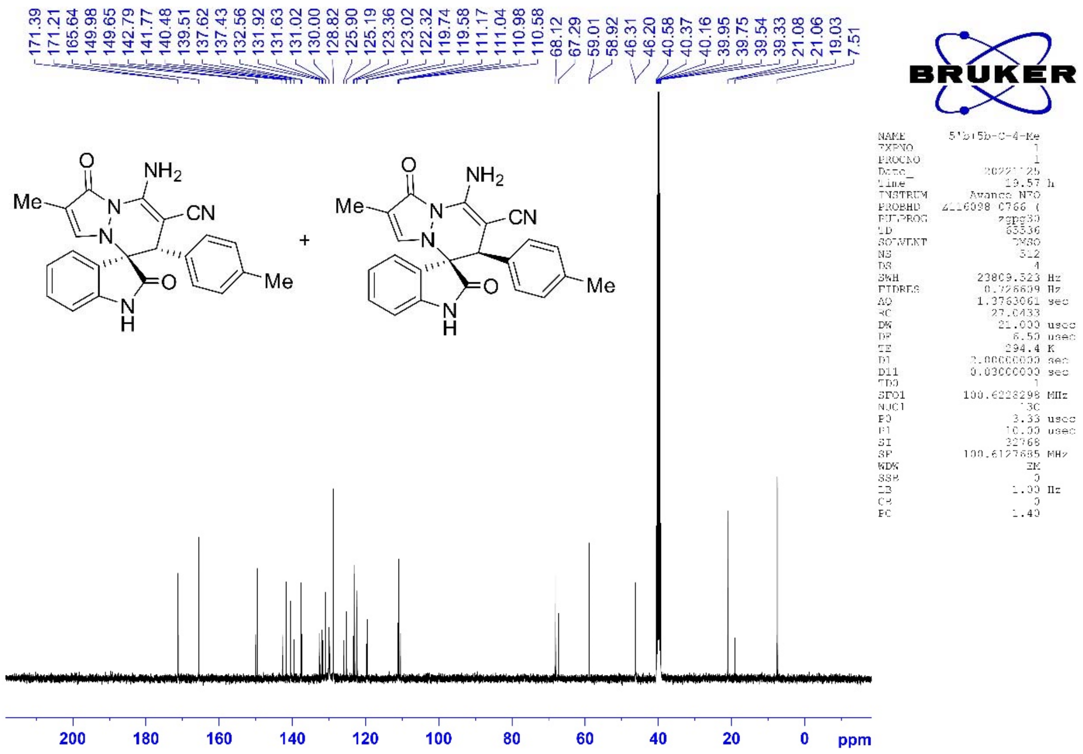

^{1}H and ^{13}C NMR Spectra for Compound **5c**+**5’c**(recrystallized from ethanol)

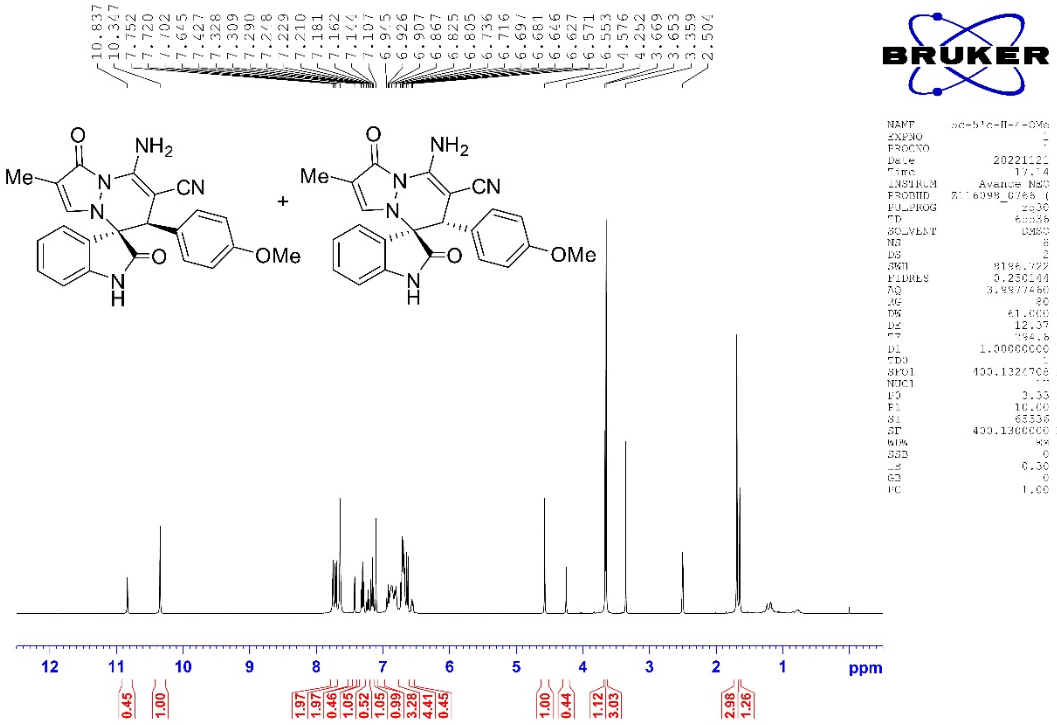

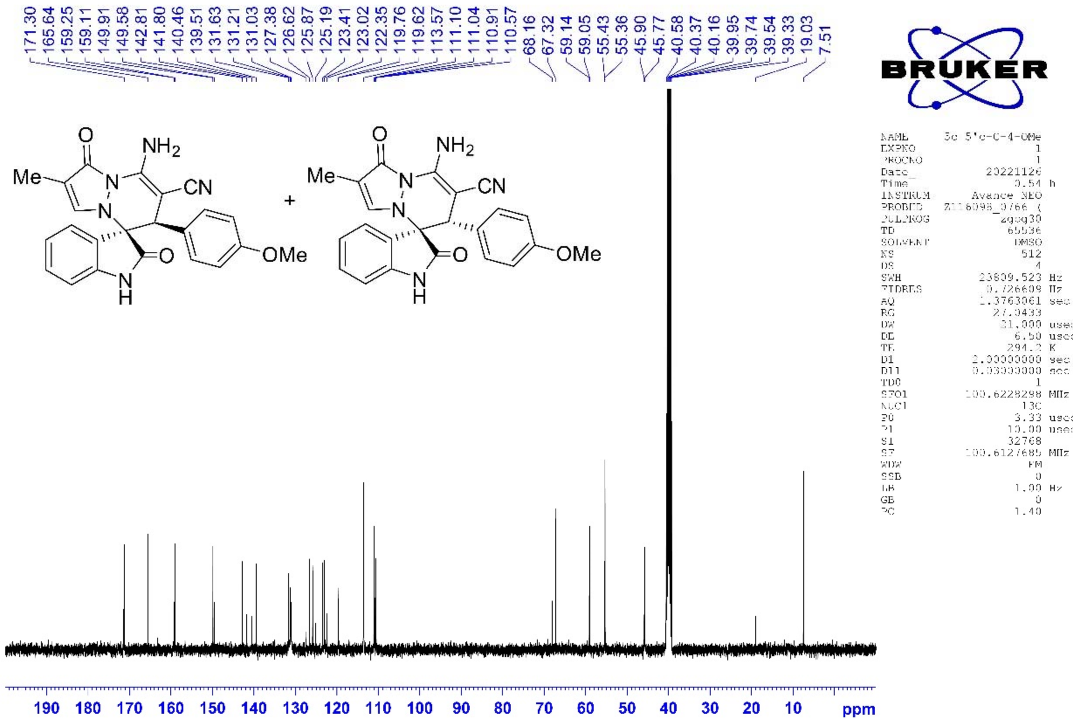

^1H, ^{13}C NMR and ^{19}F Spectra for Compound **5d** (recrystallized from ethanol)

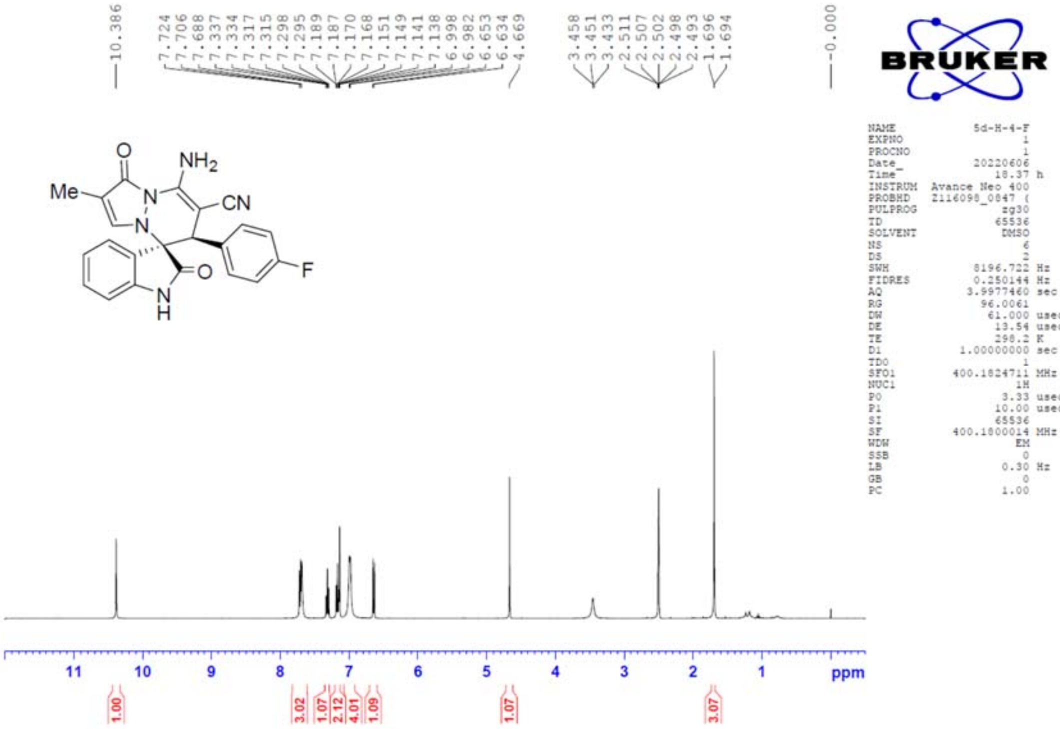

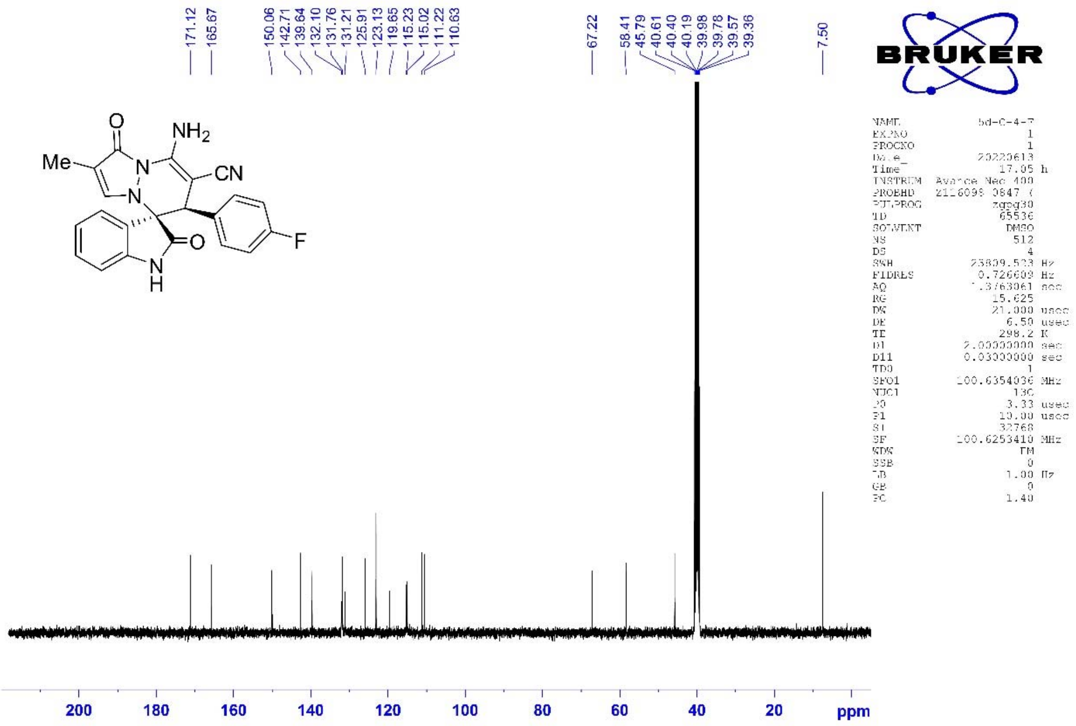
171.12
165.67
150.06
142.71
139.64
132.10
131.76
131.21
125.91
123.13
119.65
115.23
115.02
111.22
110.63
67.22
58.41
45.79
40.61
40.40
40.19
39.98
39.78
39.57
39.36
7.50
O
NH2
Me
N
N
CN
F
O
NH
H
BRUKER
NAME 5d-C-4-F
EXPNO 1
PROCNO 1
Date_ 20220613
Time 17.05 h
INSTRUM Avance Neo 400
PROBHD Z116098 0847 (
PULPROG zgpg30
TD 65536
SOLVENT DMSO
NS 512
DS 4
SWH 23809.523 Hz
FIDRES 0.726609 Hz
AQ 1.3763061 sec
RG 15.625
DW 21.000 usec
DE 6.50 usec
TE 298.2 K
D1 2.00000000 sec
D11 0.03000000 sec
TD0 1
SFO1 100.6354036 MHz
NUC1 13C
P0 3.33 usec
P1 10.00 usec
SI 32768
SF 100.6253410 MHz
WDW EM
SSB 0
LB 1.00 Hz
GB 0
PC 1.40
200
180
160
140
120
100
80
60
40
20
ppm

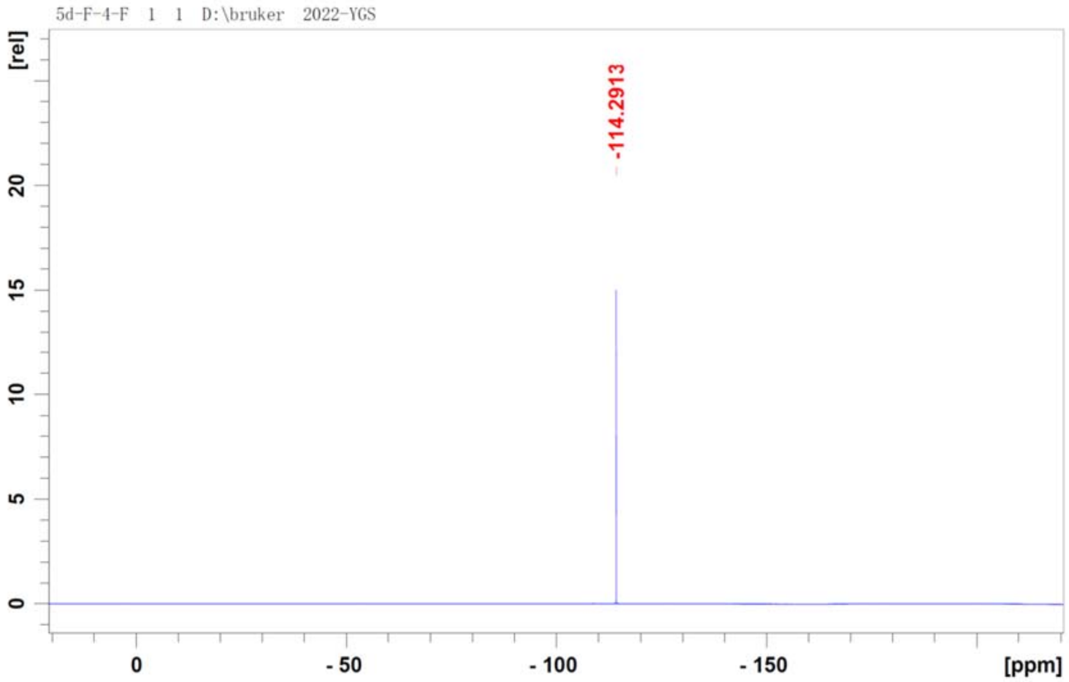
5d-F-4-F 1 1 D:\bruker 2022-YGS
[rel]
-114.2913
20
15
10
5
0
- 50
- 100
- 150
[ppm]

^{1}H and ^{13}C NMR Spectra for Compound **5e** (recrystallized from ethanol)

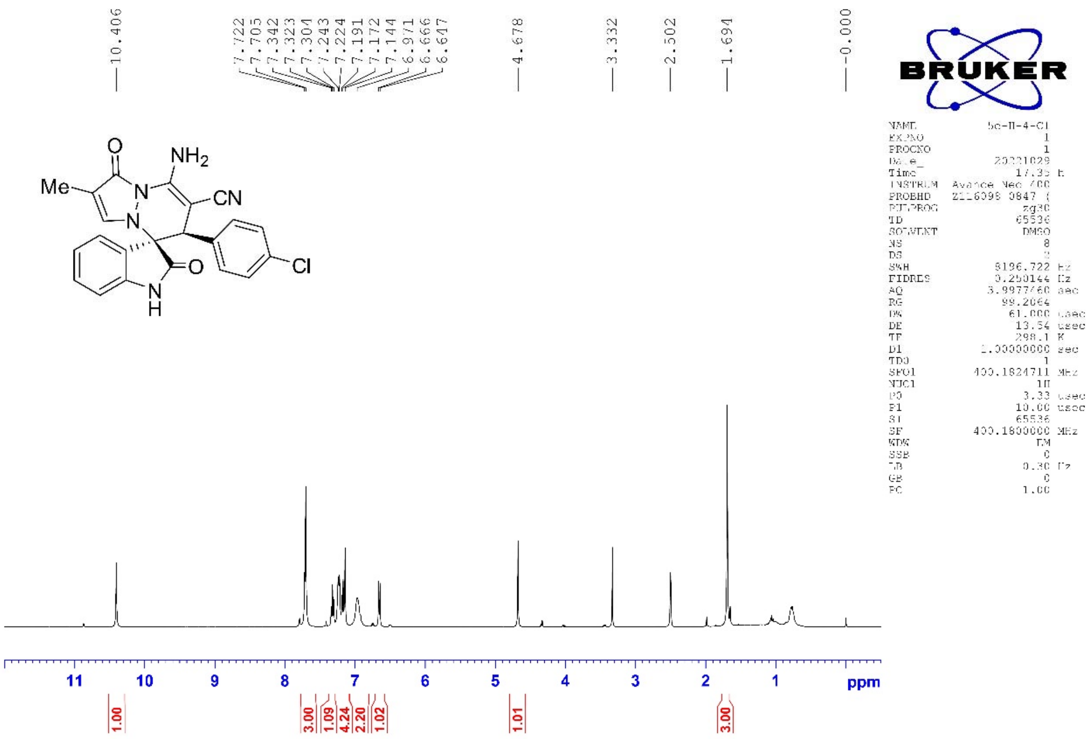

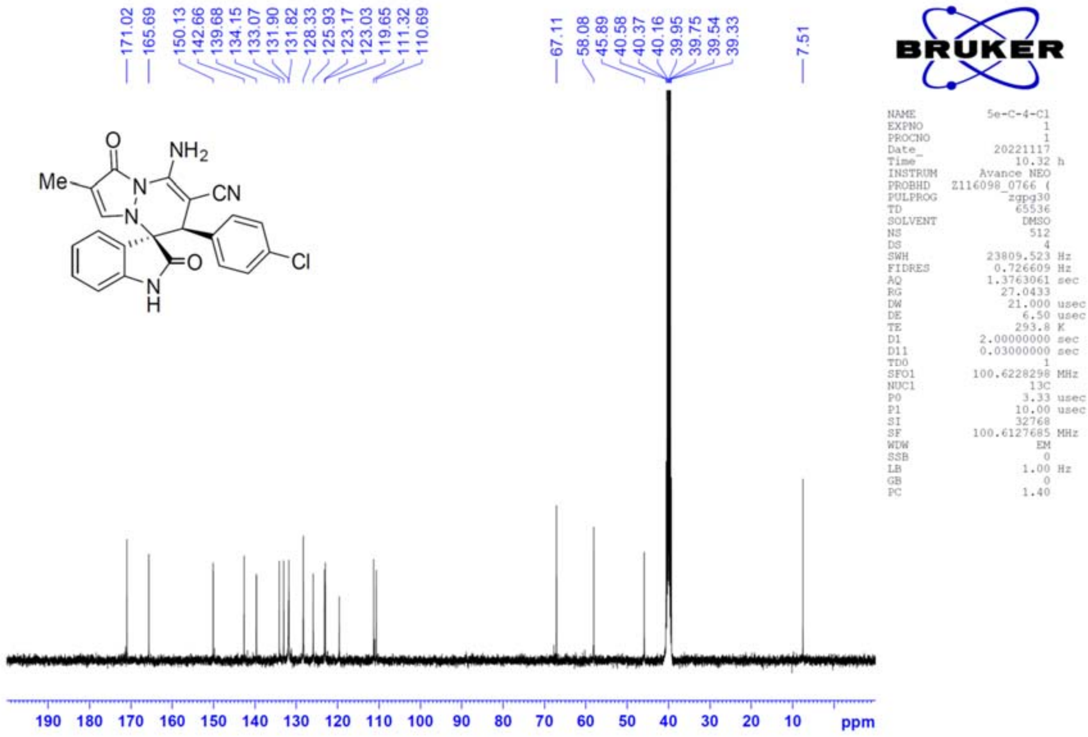

^{1}H and ^{13}C NMR Spectra for Compound **5f** (containing a few of **5’f**; recrystallized from ethanol)

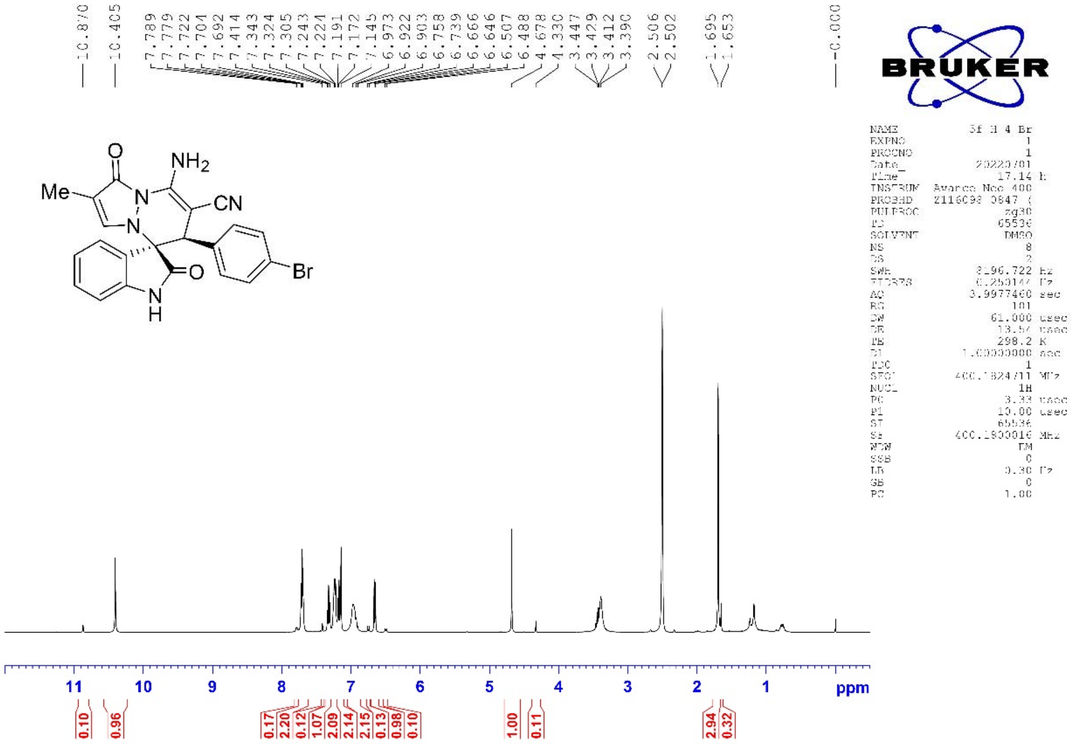

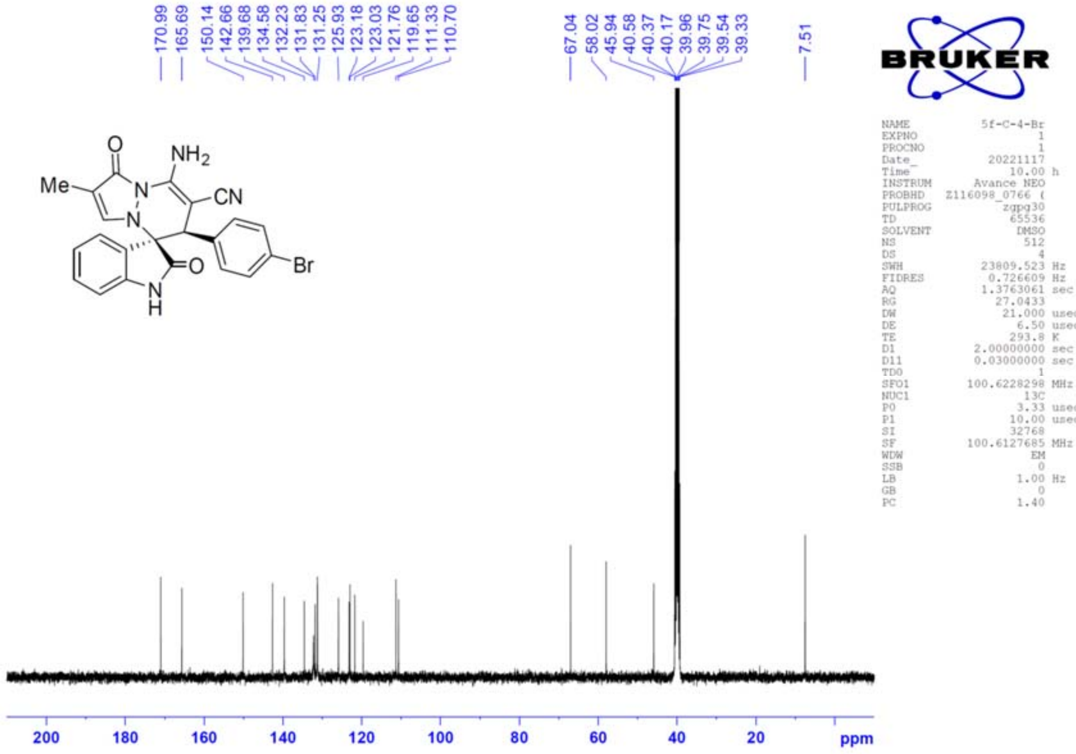

^{1}H and ^{13}C NMR Spectra for Compound **5′g+5g**

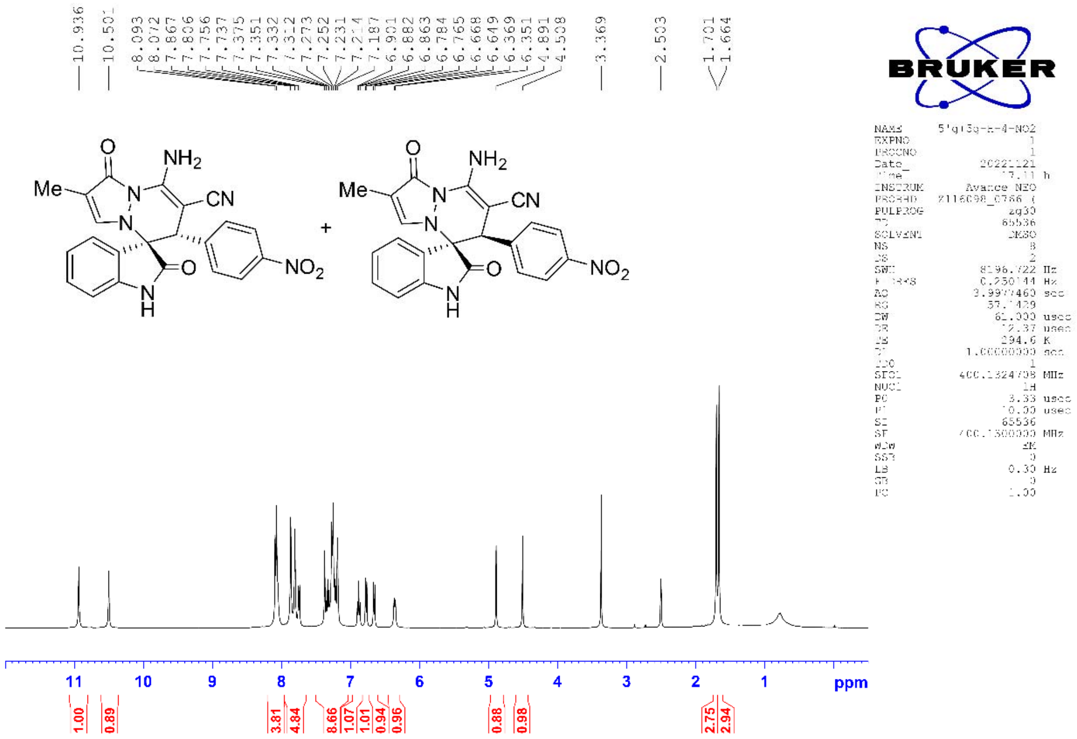

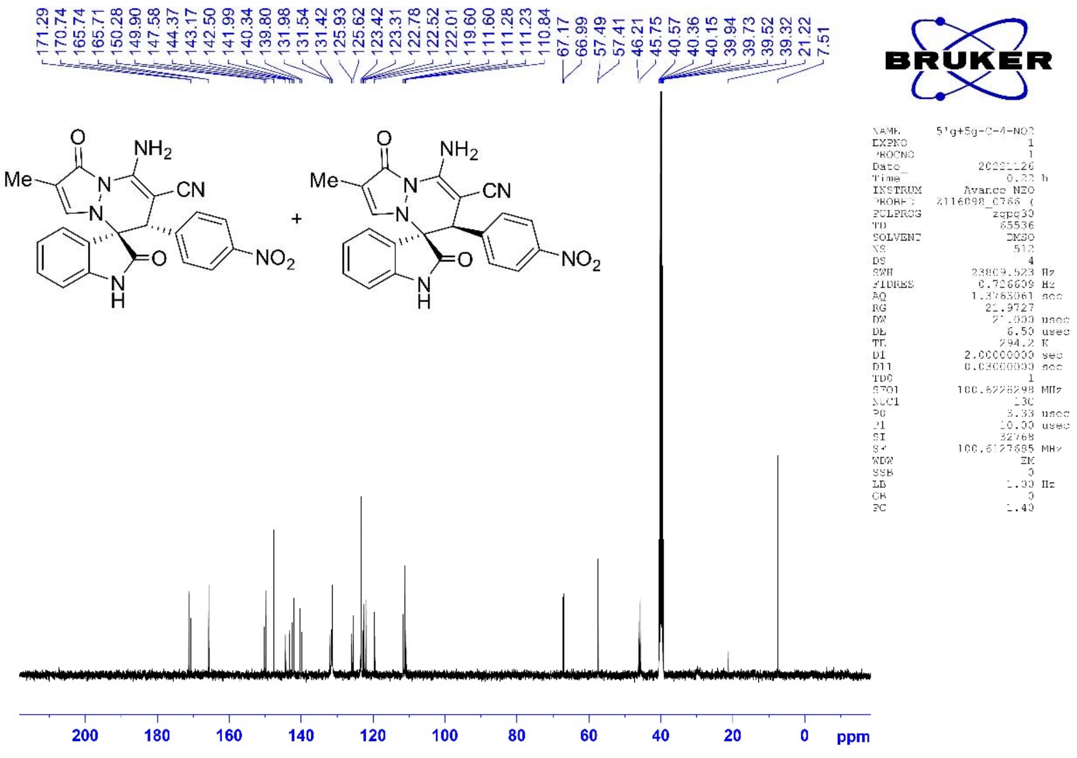

^{1}H and ^{13}C NMR Spectra for Compound **6**

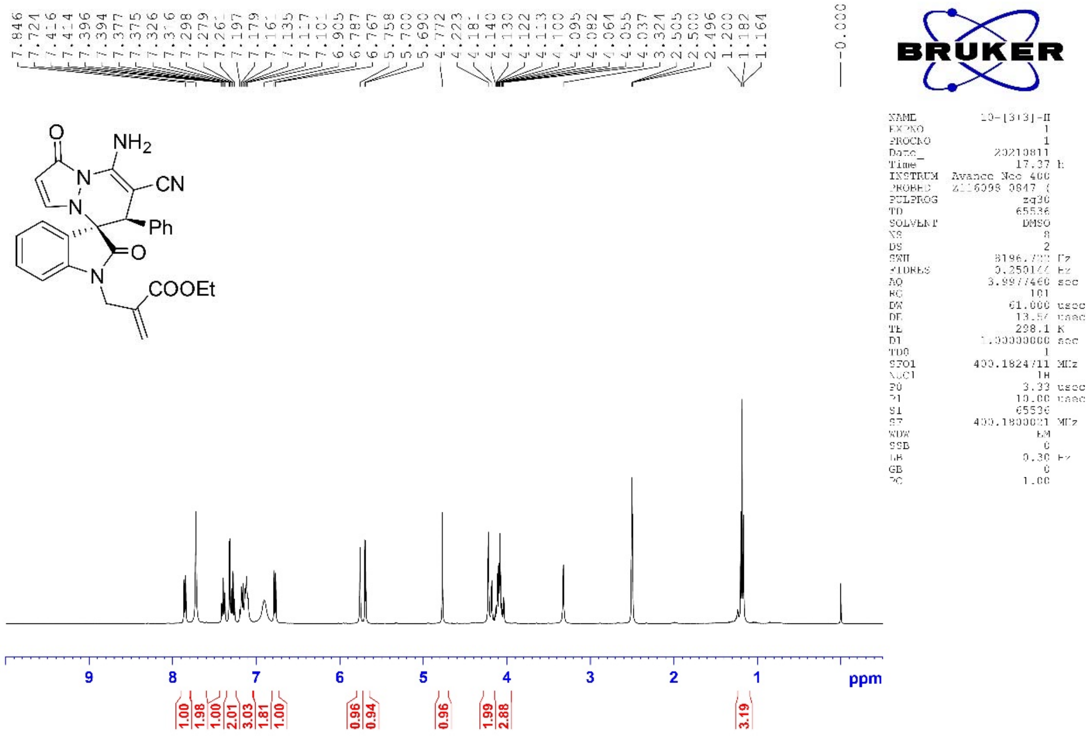

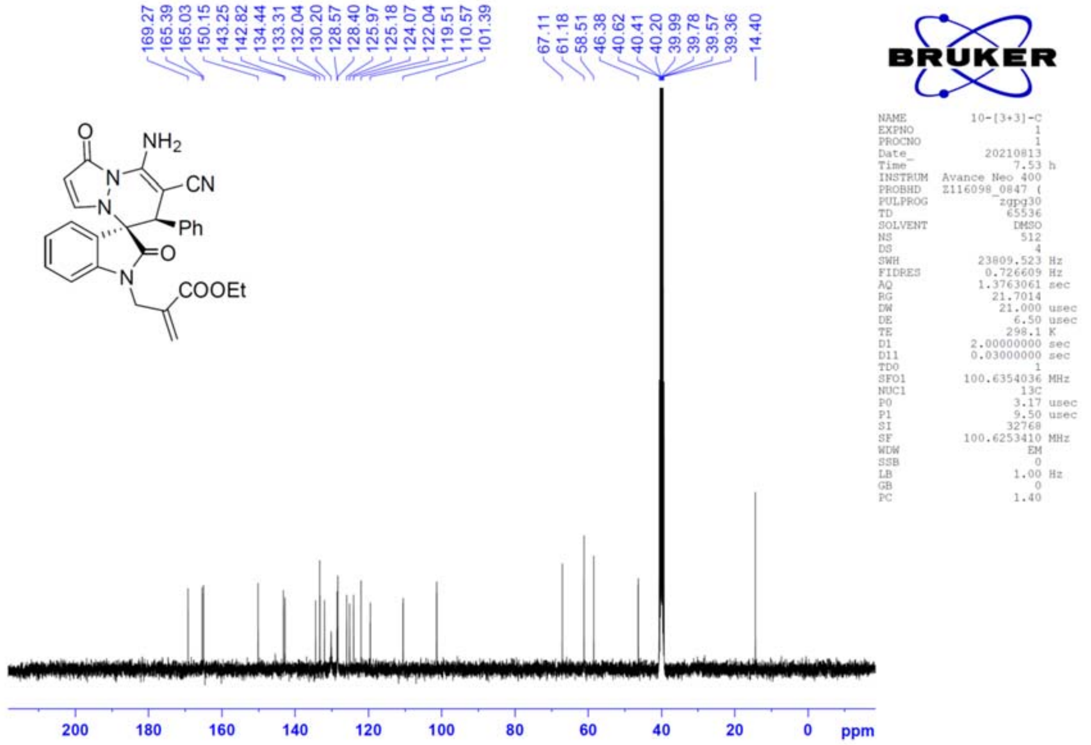

^{1}H and ^{13}C NMR Spectra for Compound **7**

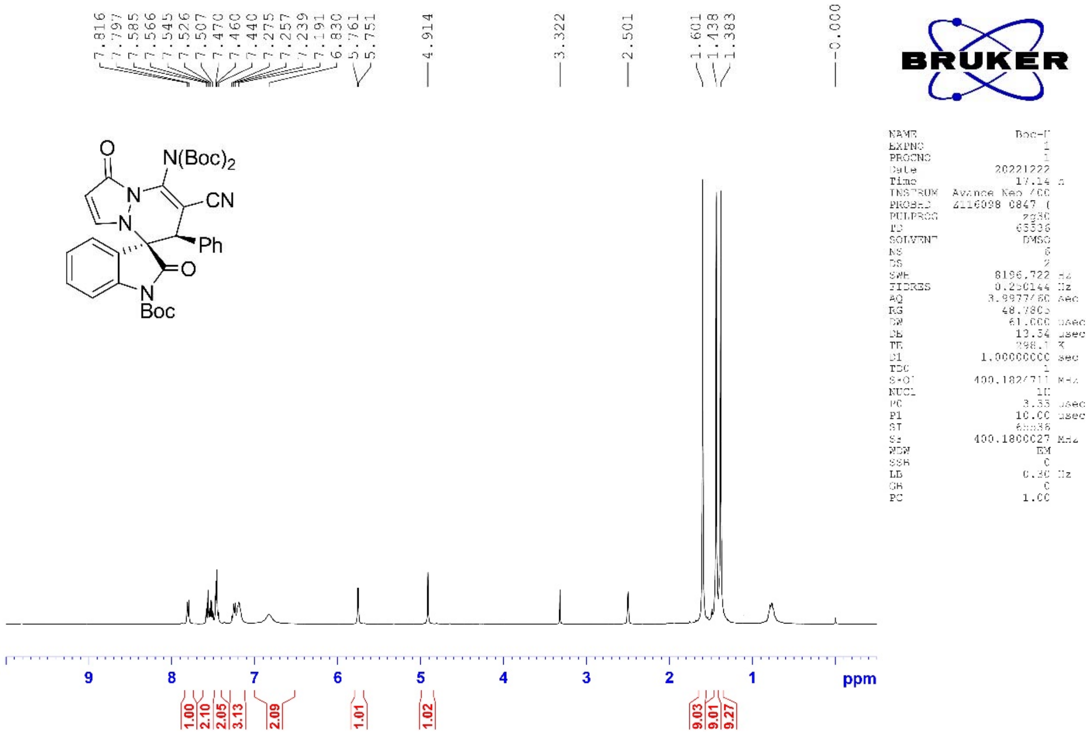

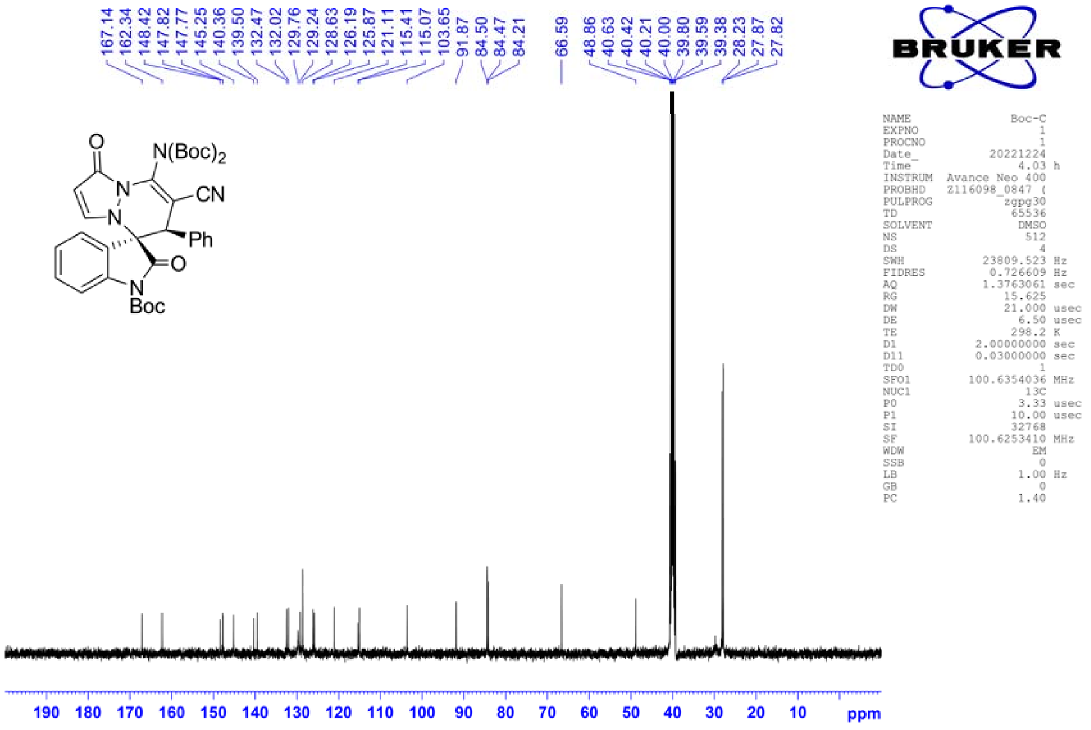

^{1}H and ^{13}C NMR Spectra for Compound **8**

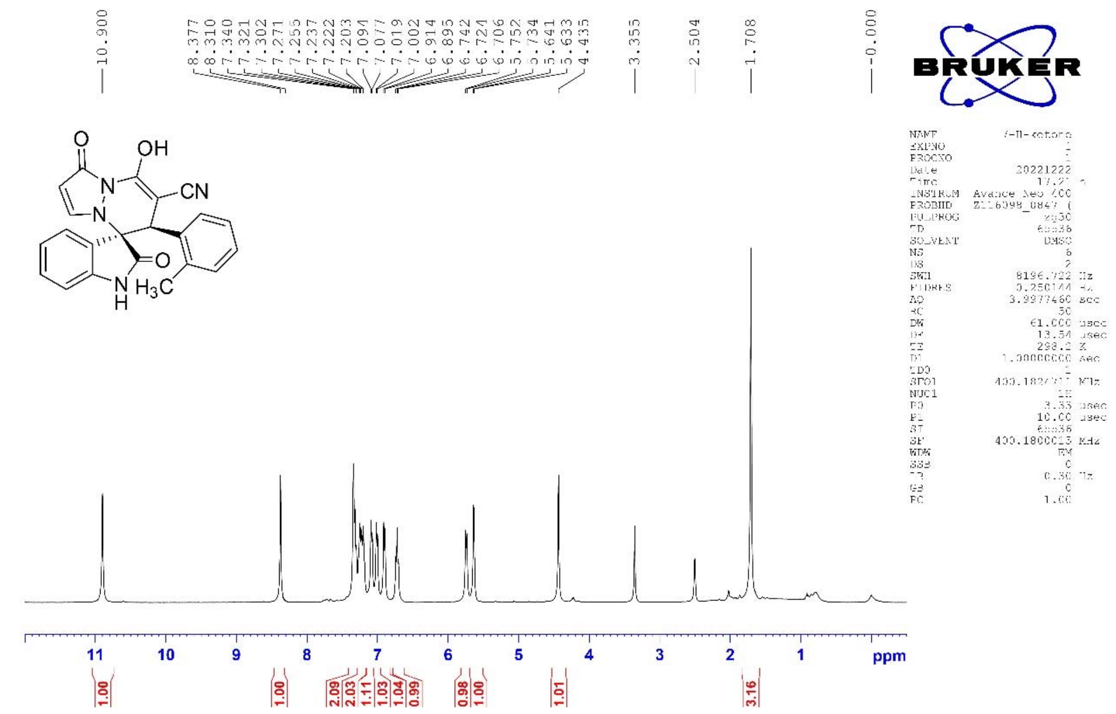

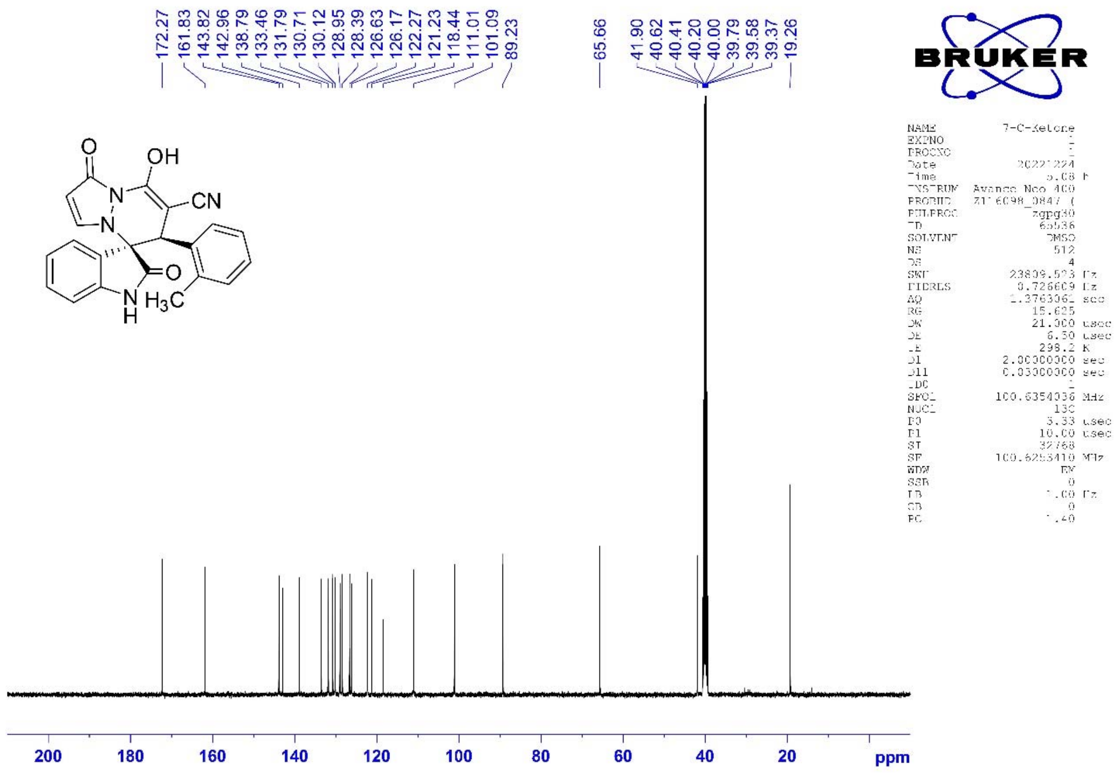

^{1}H and ^{13}C NMR Spectra for Compound **9**

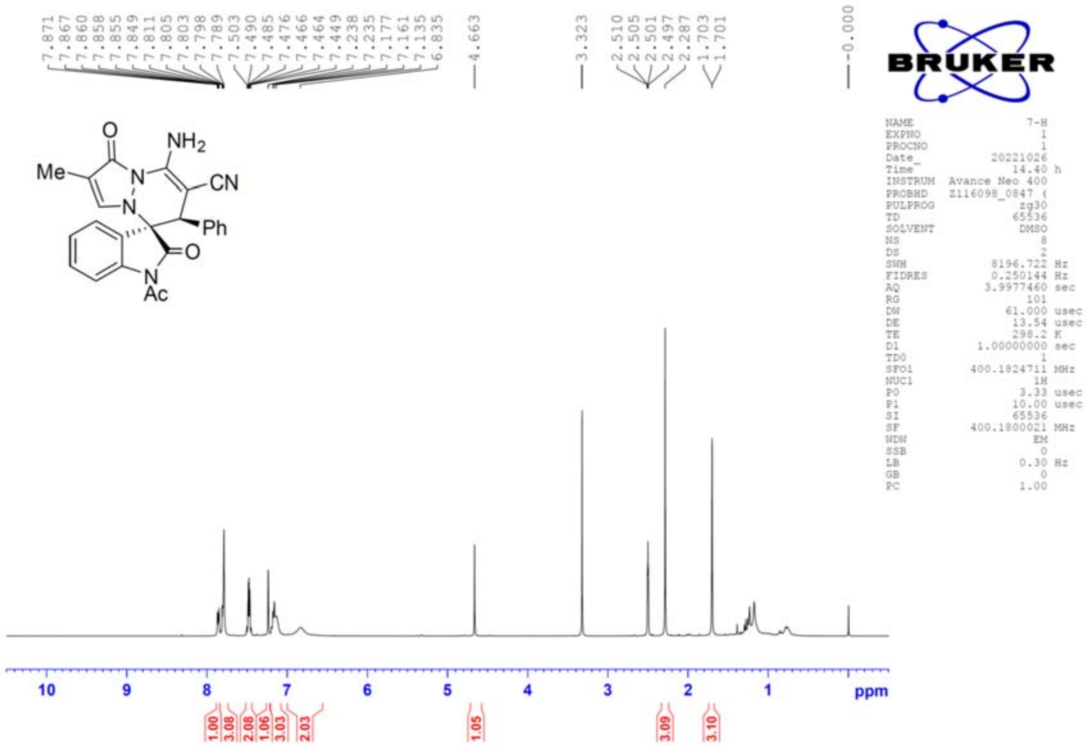

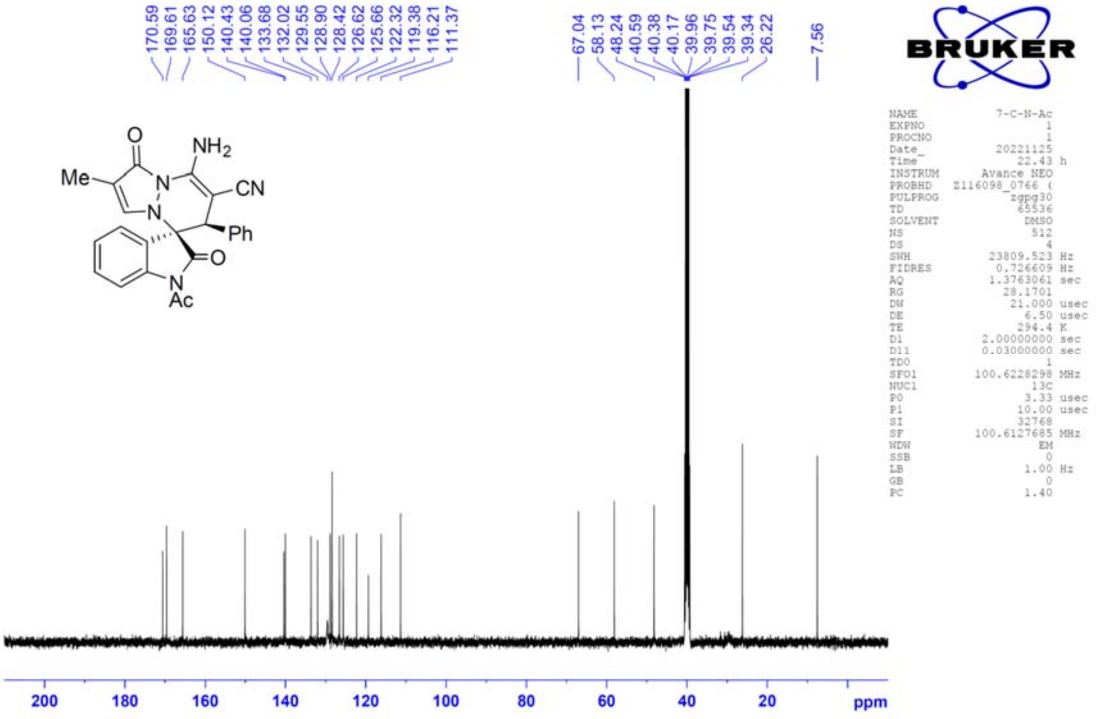

Mass spectrometry data for compound **3a**

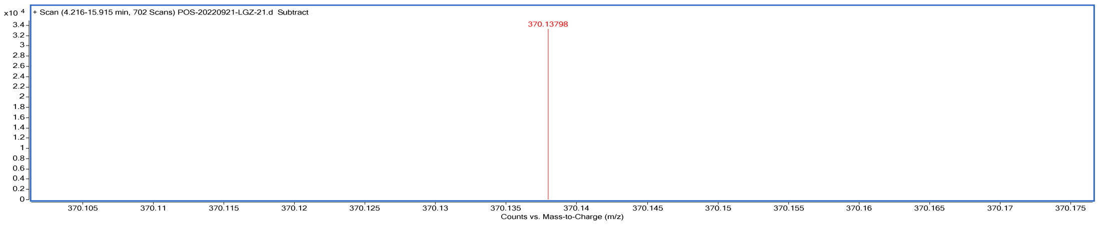

Mass spectrometry data for compound **3’b**

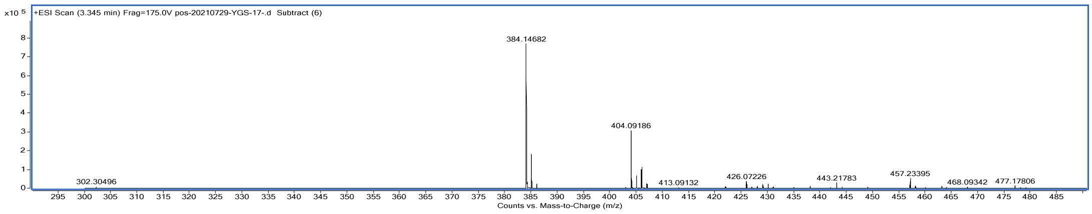

Mass spectrometry data for compound **3’c**

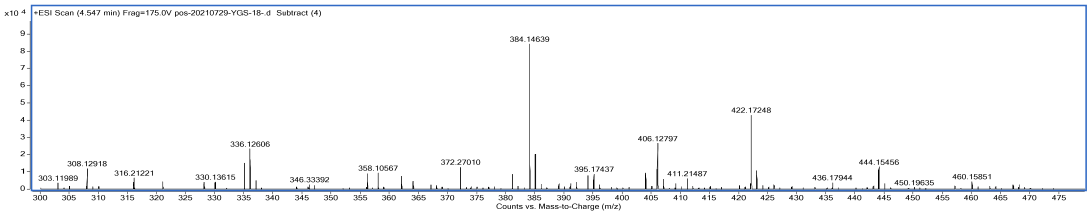

Mass spectrometry data for compound **3d**

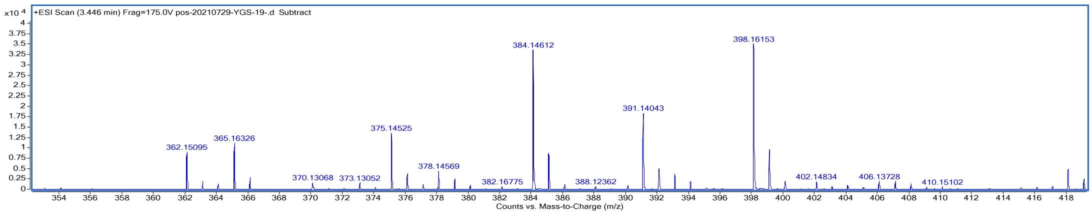

Mass spectrometry data for compound **3e**

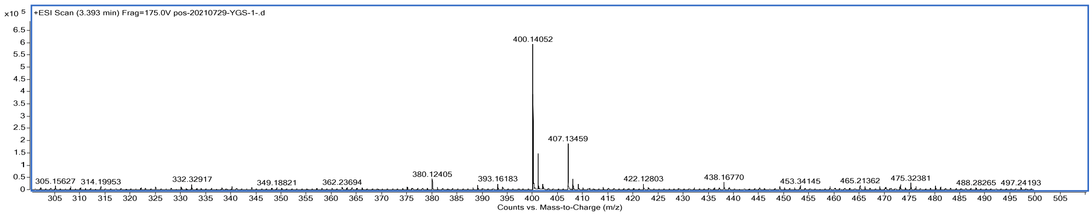

Mass spectrometry data for compound **3f**

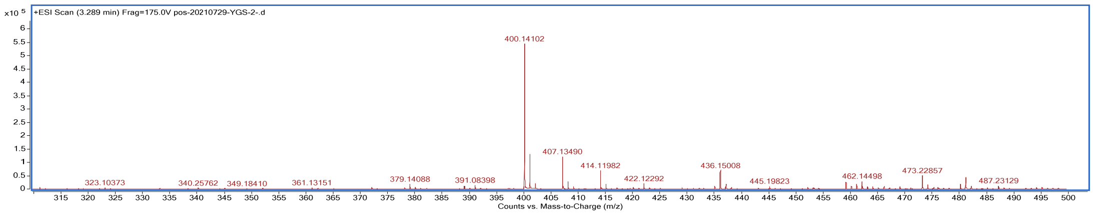

Mass spectrometry data for compound **3g**

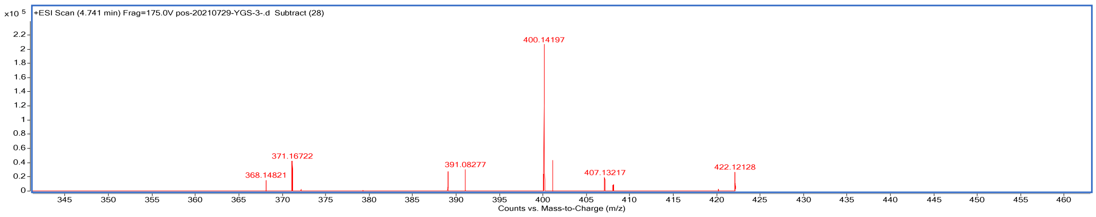

Mass spectrometry data for compound **3h**

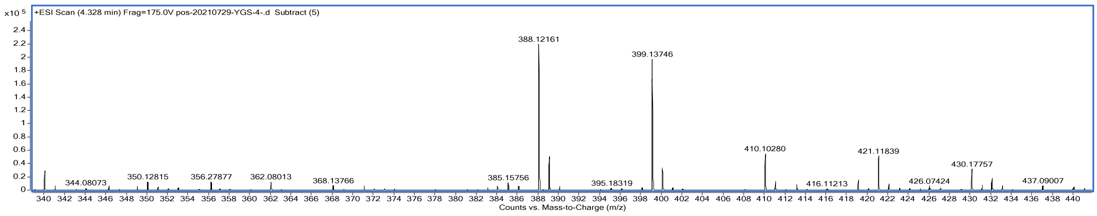

Mass spectrometry data for compound **3i**

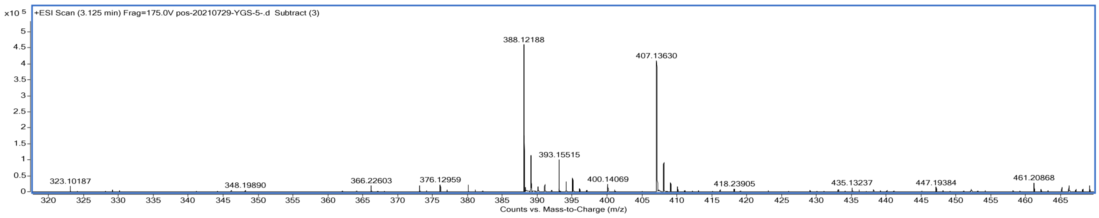

Mass spectrometry data for compound **3j**

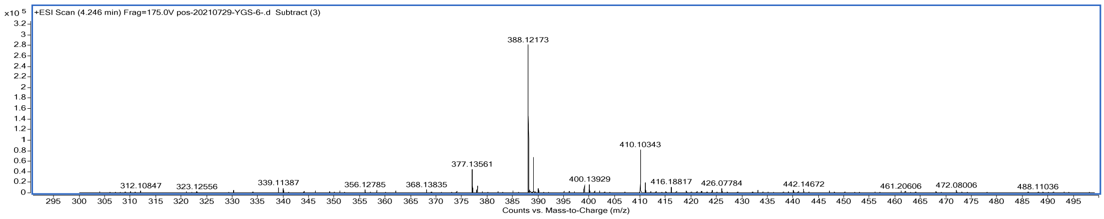

Mass spectrometry data for compound **3′k**

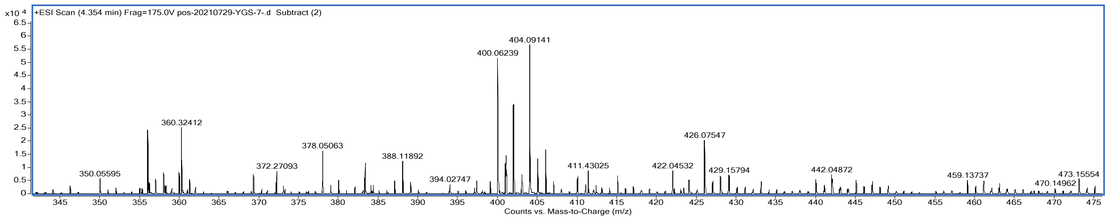

Mass spectrometry data for compound **3l**

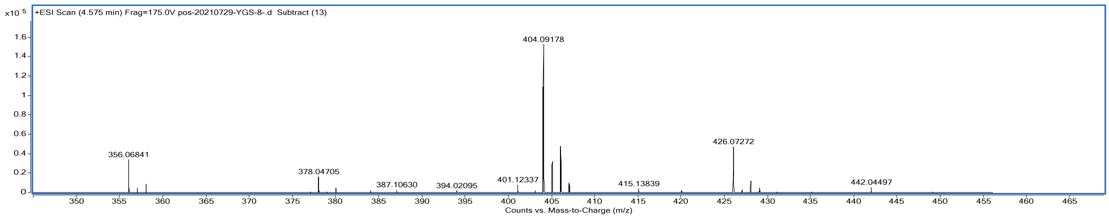

Mass spectrometry data for compound **3m**

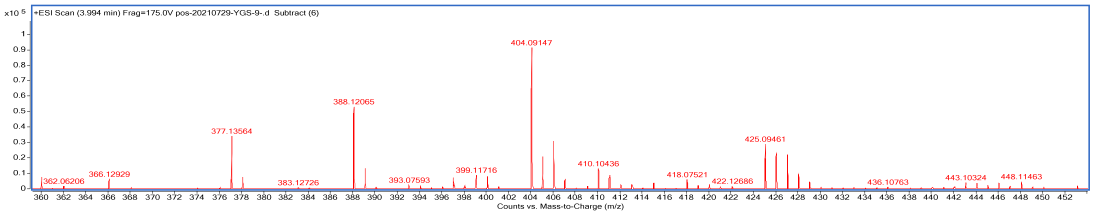

Mass spectrometry data for compound **3’n**

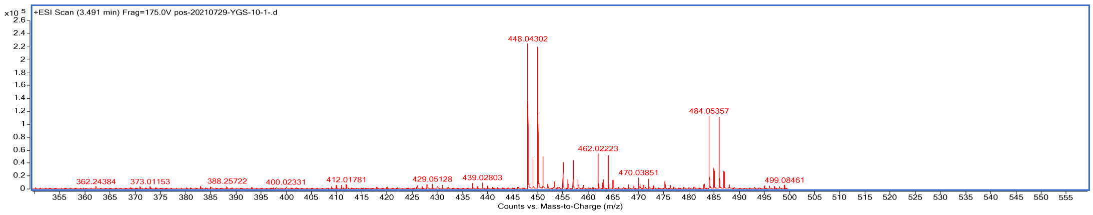

Mass spectrometry data for compound **3o**

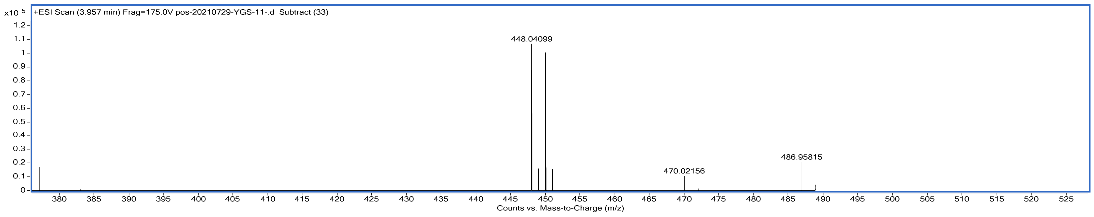

Mass spectrometry data for compound **3’p**

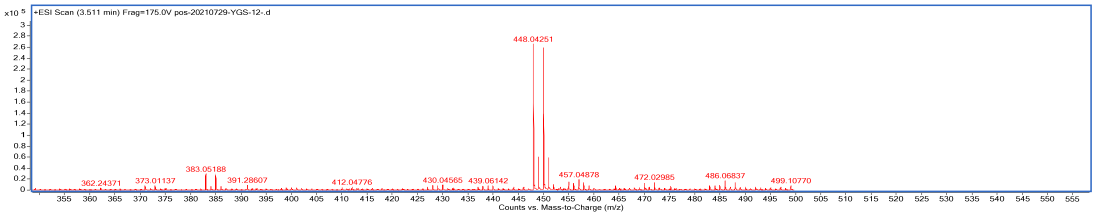

Mass spectrometry data for compounds **3q** and **3’q**

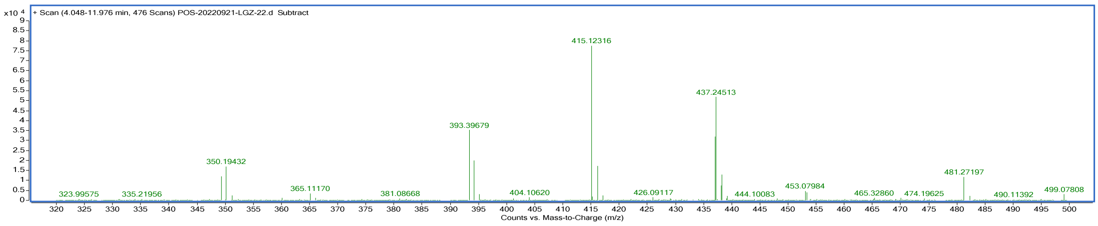

Mass spectrometry data for compound **3s**

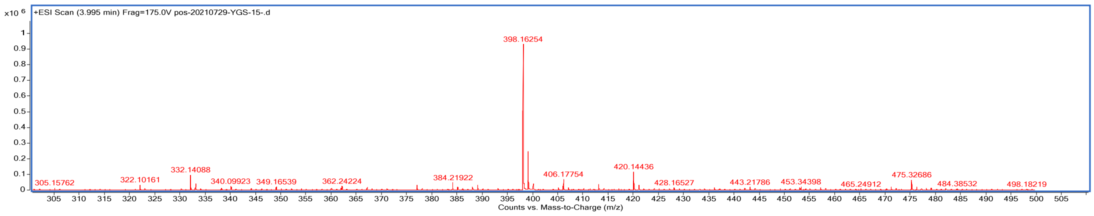

Mass spectrometry data for compound **3’t**

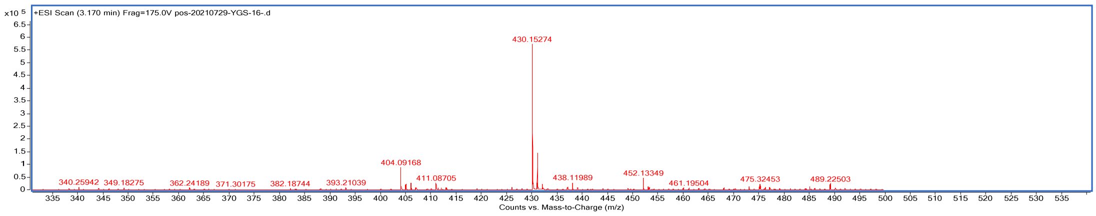

Mass spectrometry data for compounds **3u** and **3′u**

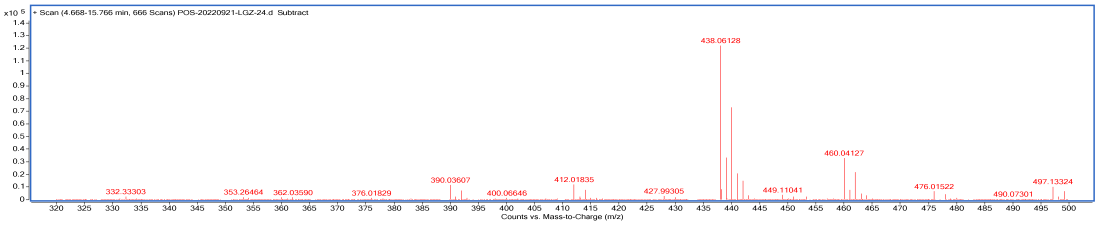

Mass spectrometry data for compounds **3v** and **3′v**

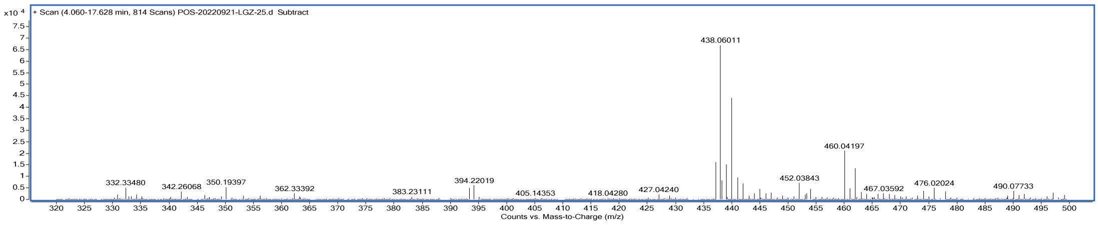

Mass spectrometry data for compounds **3w** and **3′w**

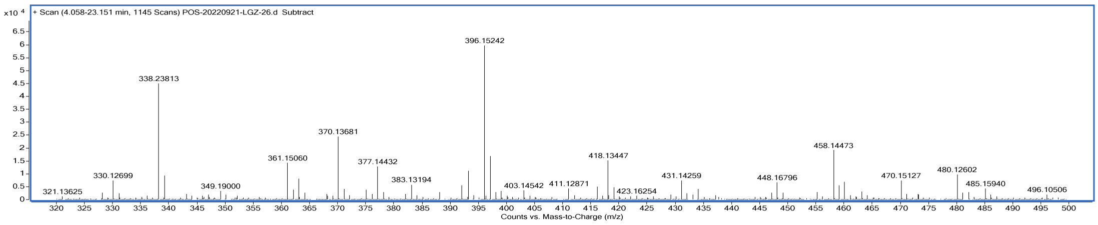

Mass spectrometry data for compound **4a**

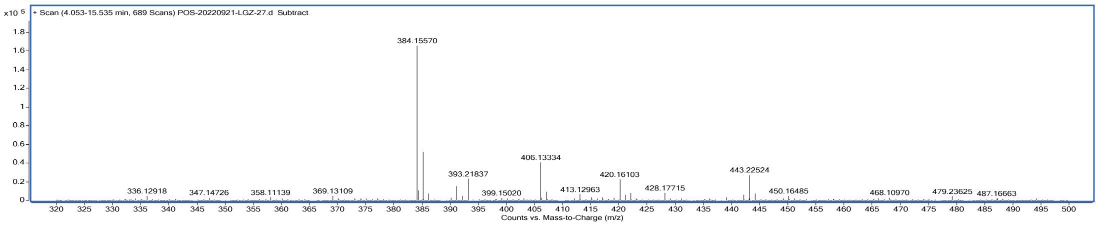

Mass spectrometry data for compounds **4b** and **4′b**

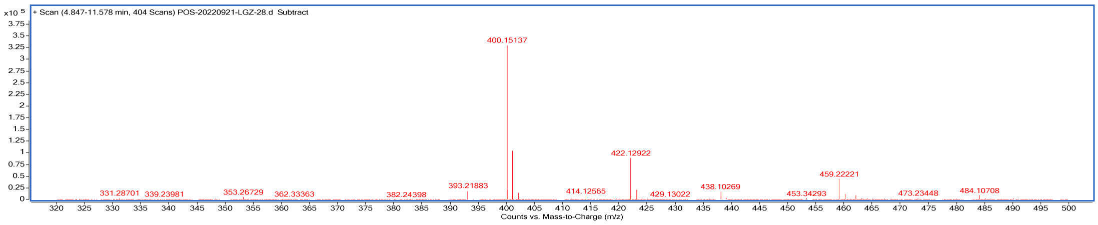

Mass spectrometry data for compound **4c**

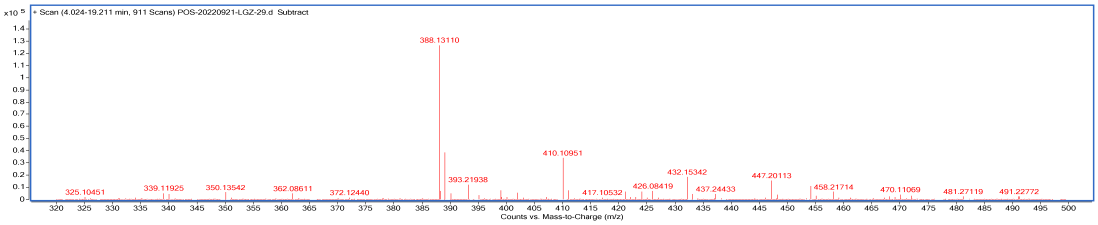

Mass spectrometry data for compound **4d**

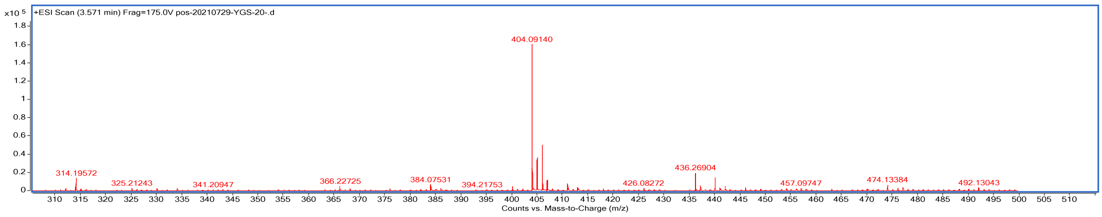

Mass spectrometry data for compound **4e**

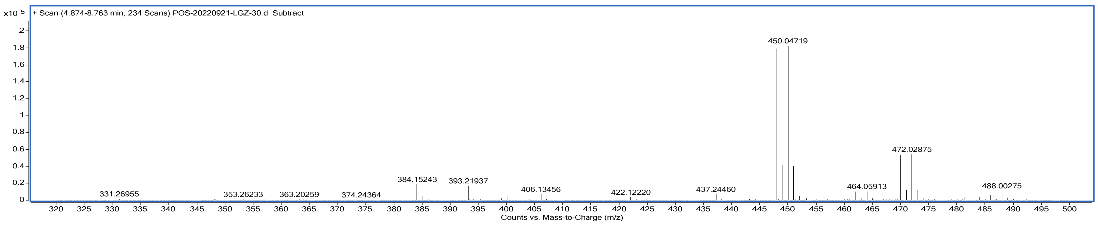

Mass spectrometry data for compound **4f**

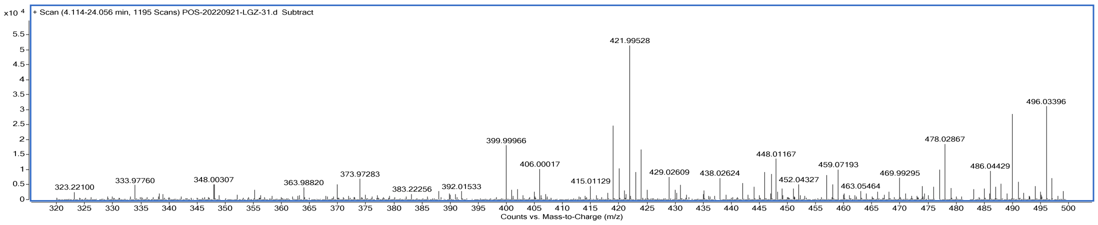

Mass spectrometry data for compound **4g**

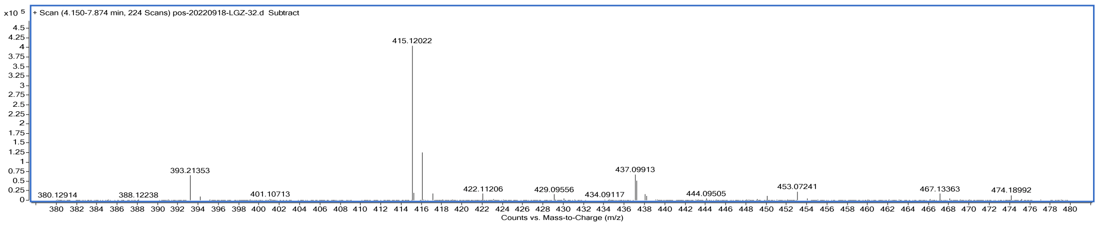

Mass spectrometry data for compounds **4h** and **4′h**

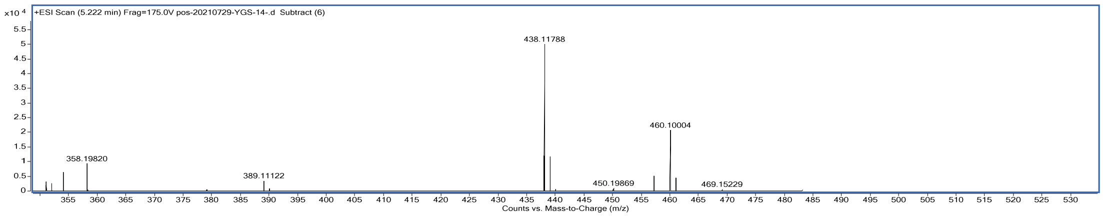

Mass spectrometry data for compound **5a**

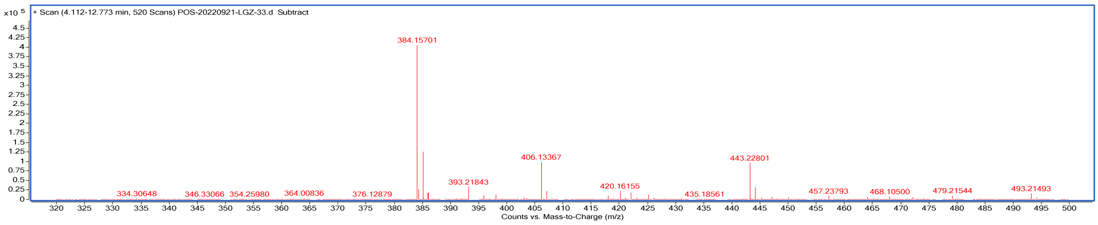

Mass spectrometry data for compounds **5b** and **5′b**

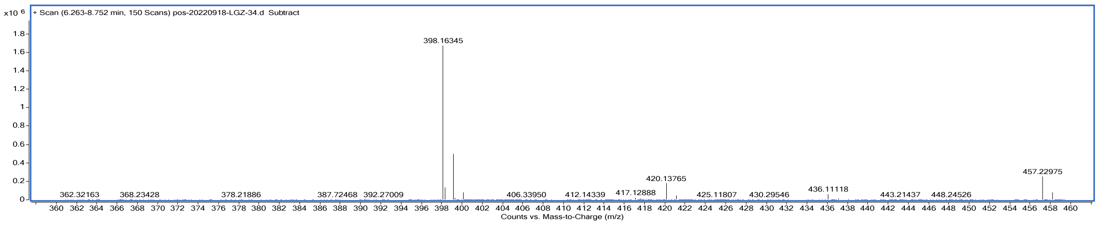

Mass spectrometry data for compounds **5c** and **5′c**

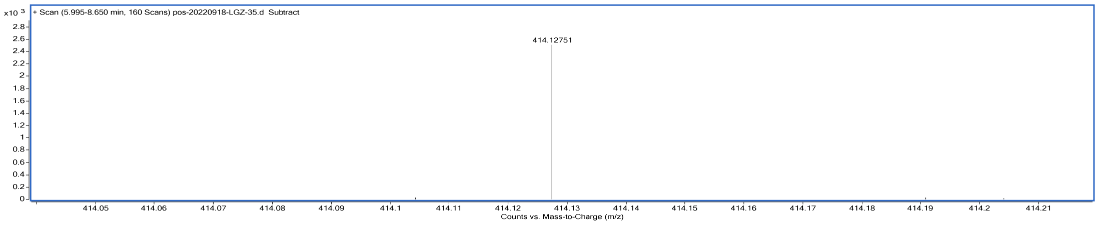

Mass spectrometry data for compound **5d**

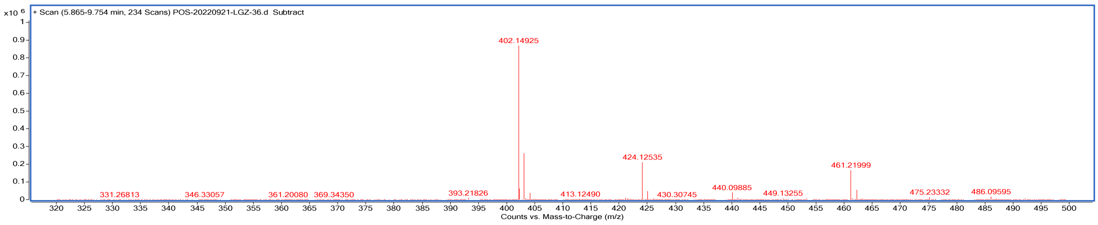

Mass spectrometry data for compound **5e**

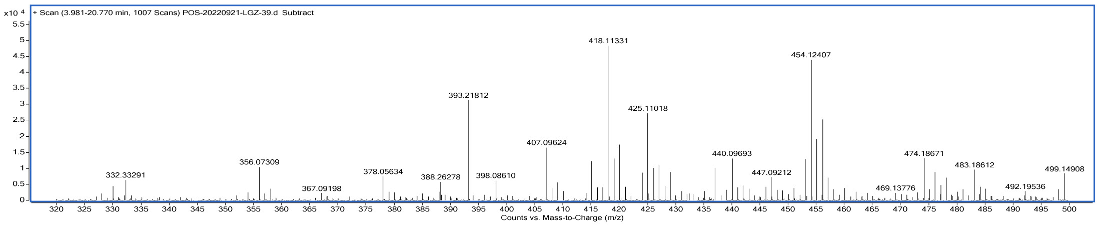

Mass spectrometry data for compound **5f**

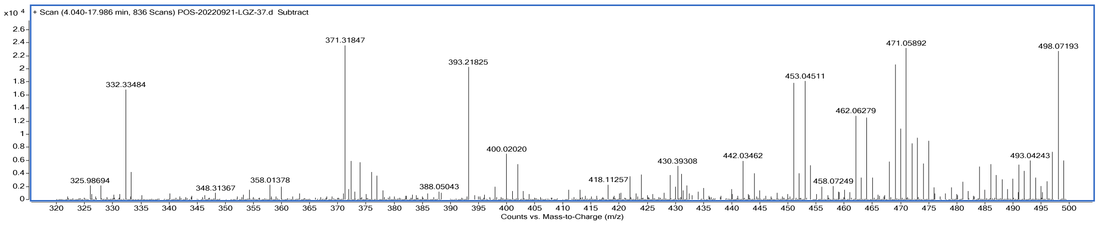

Mass spectrometry data for compounds **5g** and **5′g**

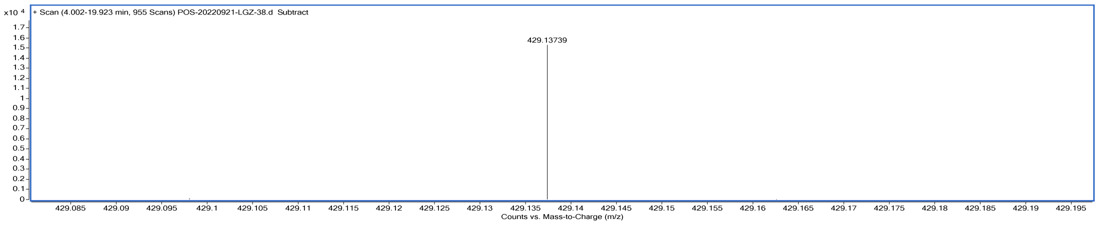

Mass spectrometry data for compound **6**

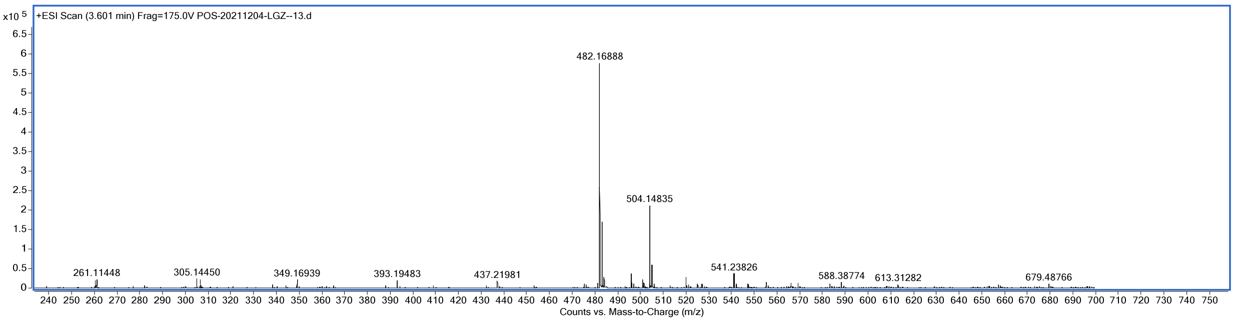

Mass spectrometry data for compound **7**

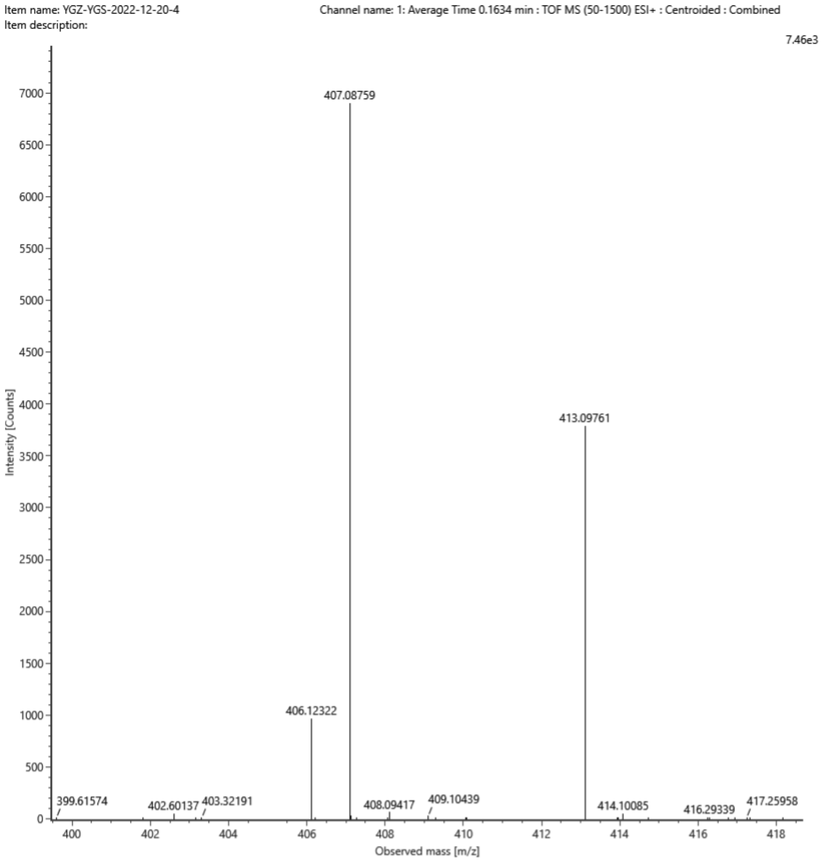

Mass spectrometry data for compound **8**

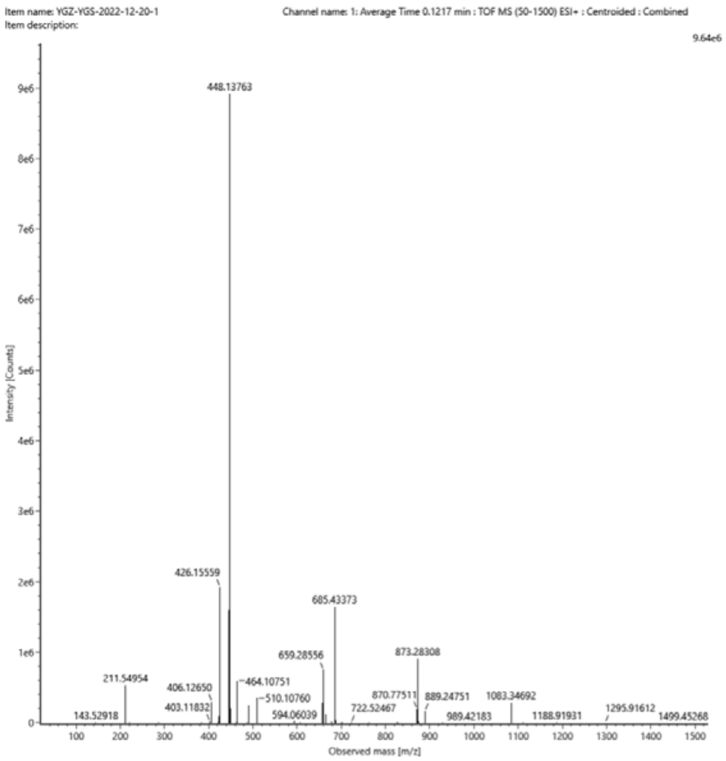

Mass spectrometry data for compound **9**

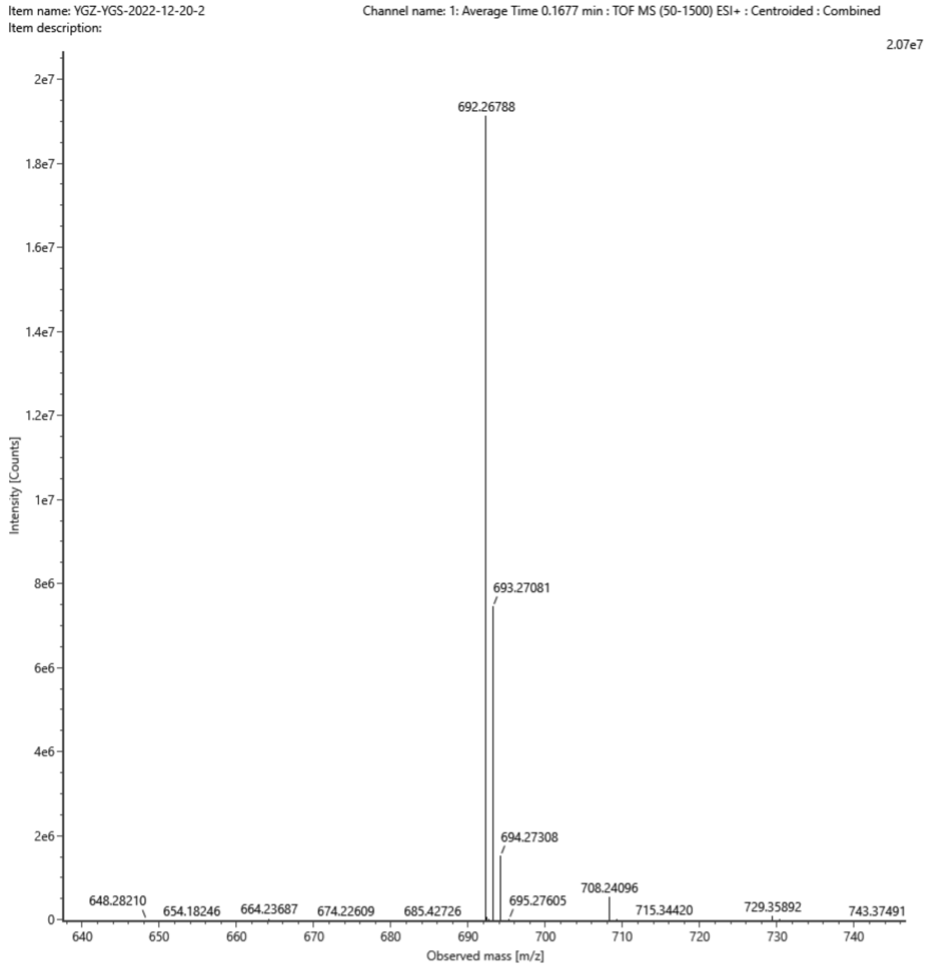

Data of X-ray crystal structure for **4a** (CCDC: 2151254).

Datablock 1 - ellipsoid plot

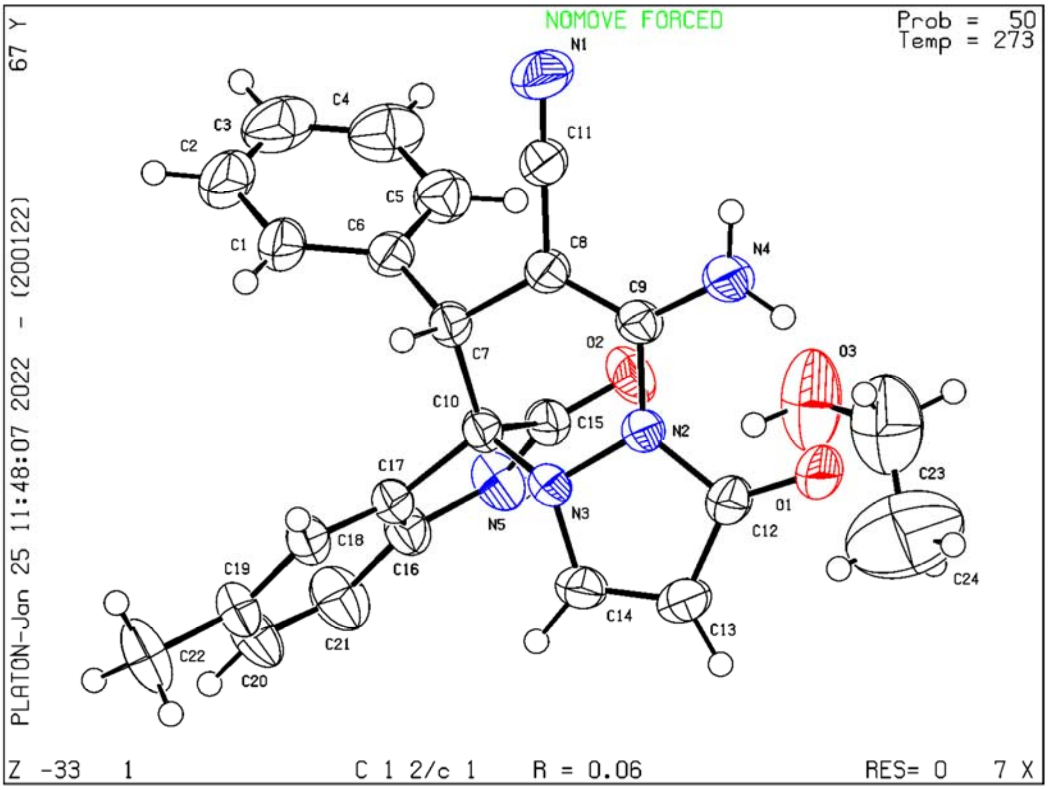

checkCIF/PLATON report

You have not supplied any structure factors. As a result the full set of tests cannot be run.

THIS REPORT IS FOR GUIDANCE ONLY. IF USED AS PART OF A REVIEW PROCEDURE FOR PUBLICATION, IT SHOULD NOT REPLACE THE EXPERTISE OF AN EXPERIENCED CRYSTALLOGRAPHIC REFEREE.

No syntax errors found. CIF dictionary Interpreting this report

Datablock: 1

Bond precision:	C-C = 0.0034 A		Wavelength=0.71073
Cell:	a=24.4316(13)	b=9.4975(5)	c=19.8479(10)
	alpha=90	beta=103.4014(17)	gamma=90
Temperature:	273 K		

	Calculated	Reported
Volume	4480.1(4)	4480.1(4)
Space group	C 2/c	C 1 2/c 1
Hall group	-C 2yc	-C 2yc
Moiety formula	C22 H17 N5 O2, C2 H6 O	C22 H17 N5 O2, C2 H6 O
Sum formula	C24 H23 N5 O3	C24 H23 N5 O3
Mr	429.47	429.47
Dx,g cm-3	1.273	1.273
Z	8	8
Mu (mm-1)	0.087	0.087
F000	1808.0	1808.0
F000'	1808.74	
h,k,lmax	31,12,25	31,12,25
Nref	5150	5129
Tmin,Tmax	0.973,0.989	0.681,0.746
Tmin'	0.969	

Correction method= # Reported T Limits: Tmin=0.681 Tmax=0.746
AbsCorr = MULTI-SCAN

Data completeness= 0.996 Theta(max)= 27.529

R(reflections)= 0.0593(3540) wR2(reflections)= 0.1720(5129)

S = 1.025 Npar= 292

```
The following ALERTS were generated. Each ALERT has the format
       test-name_ALERT_alert-type_alert-level.
Click on the hyperlinks for more details of the test.
```

```
Alert level C
PLAT094_ALERT_2_C Ratio of Maximum / Minimum Residual Density ....       2.10 Report
PLAT260_ALERT_2_C Large Average Ueq of Residue Including        O3      0.129 Check
PLAT360_ALERT_2_C Short  C(sp3)-C(sp3) Bond  C23      - C24      .       1.42 Ang.
```

```
Alert level G
PLAT007_ALERT_5_G Number of Unrefined Donor-H Atoms .............          4 Report
PLAT199_ALERT_1_G Reported _cell_measurement_temperature ..... (K)       273 Check
PLAT200_ALERT_1_G Reported   _diffrn_ambient_temperature ..... (K)       273 Check
PLAT793_ALERT_4_G Model has Chirality at C7             (Centro SPGR)      R Verify
PLAT793_ALERT_4_G Model has Chirality at C10            (Centro SPGR)      R Verify
```

```
   0 ALERT level A = Most likely a serious problem - resolve or explain
   0 ALERT level B = A potentially serious problem, consider carefully
   3 ALERT level C = Check. Ensure it is not caused by an omission or oversight
   5 ALERT level G = General information/check it is not something unexpected

   2 ALERT type 1 CIF construction/syntax error, inconsistent or missing data
   3 ALERT type 2 Indicator that the structure model may be wrong or deficient
   0 ALERT type 3 Indicator that the structure quality may be low
   2 ALERT type 4 Improvement, methodology, query or suggestion
   1 ALERT type 5 Informative message, check
```

It is advisable to attempt to resolve as many as possible of the alerts in all categories. Often the minor alerts point to easily fixed oversights, errors and omissions in your CIF or refinement strategy, so attention to these fine details can be worthwhile. In order to resolve some of the more serious problems it may be necessary to carry out additional measurements or structure refinements. However, the purpose of your study may justify the reported deviations and the more serious of these should normally be commented upon in the discussion or experimental section of a paper or in the "special_details" fields of the CIF. checkCIF was carefully designed to identify outliers and unusual parameters, but every test has its limitations and alerts that are not important in a particular case may appear. Conversely, the absence of alerts does not guarantee there are no aspects of the results needing attention. It is up to the individual to critically assess their own results and, if necessary, seek expert advice.

Publication of your CIF in IUCr journals

A basic structural check has been run on your CIF. These basic checks will be run on all CIFs submitted for publication in IUCr journals (*Acta Crystallographica*, *Journal of Applied Crystallography*, *Journal of Synchrotron Radiation*); however, if you intend to submit to *Acta Crystallographica Section C* or *E* or *IUCrData*, you should make sure that full publication checks are run on the final version of your CIF prior to submission.

Publication of your CIF in other journals

Please refer to the *Notes for Authors* of the relevant journal for any special instructions relating to CIF submission.

PLATON version of 20/01/2022; check.def file version of 19/01/2022
